# Saturated Linkers in Two-Dimensional Covalent Organic Frameworks Boost Their Luminescence

**DOI:** 10.1021/jacs.3c03614

**Published:** 2023-06-20

**Authors:** Meijia Yang, Hiroki Hanayama, Long Fang, Matthew A. Addicoat, Yunyu Guo, Robert Graf, Koji Harano, Jun Kikkawa, Enquan Jin, Akimitsu Narita, Klaus Müllen

**Affiliations:** † Max Planck Institute for Polymer Research, Ackermannweg 10, 55128, Mainz, Germany; ∮ State Key Laboratory of Inorganic Synthesis and Preparative Chemistry, College of Chemistry and International Center of Future Science, Jilin University, Changchun 130012, China; ‡ Organic and Carbon Nanomaterials Unit, Okinawa Institute of Science and Technology Graduate University, Kunigami-gun, Okinawa 904-0495, Japan; § Key Laboratory for Polymeric Composite and Functional Materials of Ministry of Education, School of Chemistry, Sun Yat-sen University, Guangzhou 510275, Guangdong, China; Ψ School of Science and Technology, Nottingham Trent University, Clifton Lane, Nottingham NG11 8NS, U.K.; # Center for Basic Research on Materials, National Institute for Materials Science, 1-1 Namiki, Tsukuba, Ibaraki 305-0044, Japan

## Abstract

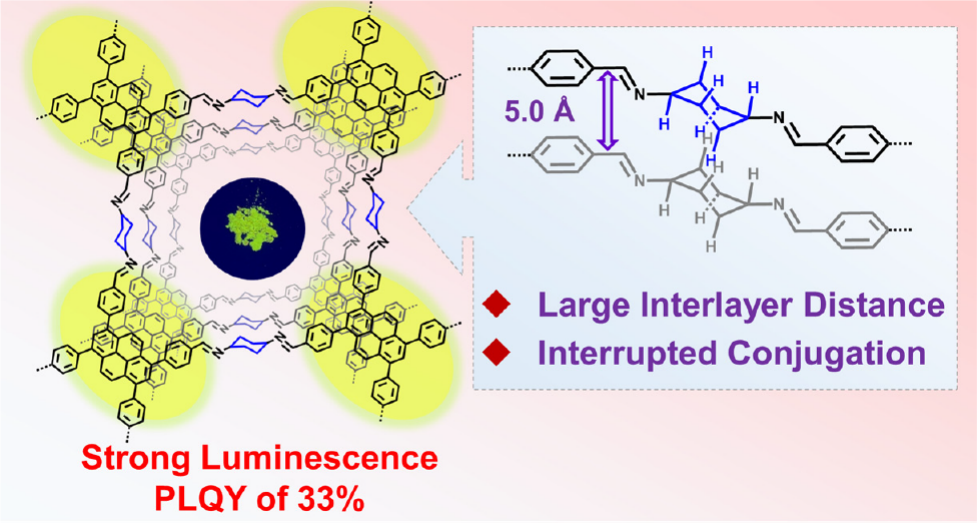

The development of highly luminescent two-dimensional covalent organic frameworks (COFs) for sensing applications remains challenging. To suppress commonly observed photoluminescence quenching of COFs, we propose a strategy involving interrupting the intralayer conjugation and interlayer interactions using cyclohexane as the linker unit. By variation of the building block structures, imine-bonded COFs with various topologies and porosities are obtained. Experimental and theoretical analyses of these COFs disclose high crystallinity and large interlayer distances, demonstrating enhanced emission with record-high photoluminescence quantum yields of up to 57% in the solid state. The resulting cyclohexane-linked COF also exhibits excellent sensing performance for the trace recognition of Fe^3+^ ions, explosive and toxic picric acid, and phenyl glyoxylic acid as metabolites. These findings inspire a facile and general strategy to develop highly emissive imine-bonded COFs for detecting various molecules.

## Introduction

Two-dimensional covalent organic frameworks (2D COFs) incorporate predesigned organic units into crystalline lattices through covalent bonds.^1,2^ Their topology is determined by the geometry and size of the monomeric building blocks, which can also make possible versatile chemical functions.^3^ Due to their (i) ample surface areas, (ii) ordered channels, and (iii) controllable pore sizes, 2D COFs show promise for applications including gas and energy storage,^4,5^ chemical separation^6,7^ and sensing,^8−12^ photodetection,^13^ and photocatalysis.^14−16^

For photoluminescence (PL)-based sensing, 2D COFs can not only provide multiple binding sites but also expand and amplify the response.^17−19^ Compared to many other PL sensors, 2D COFs allow quick removal of analytes and thus require short recovery times.^9−11^ Moreover, the periodic one-dimensional (1D) pore channels of 2D COFs facilitate mass transfer and molecular selection, thus minimizing interference from competing guest species.^12^ Among various polymerization methods, Schiff base formation to generate imine bonds has been most commonly employed in 2D COF synthesis.^20^ Imine-bonded 2D COFs exhibit high crystallinity,^2^ porosity,^3^ and stability against moisture^12^ and provide a library of multifunctional frameworks.^21^ However, obtaining highly luminescent imine-bonded 2D COFs is still challenging due to severe PL quenching, which hinders practical applications. Several plausible mechanisms have been discussed to explain PL quenching in 2D COFs,^22^ including aggregation-caused quenching (ACQ)^23^ and photoinduced charge separation,^24^ but the PL quenching issue in 2D COFs in general has not been resolved.

To prevent ACQ in 2D COFs, aggregation-induced emission (AIE) is a promising approach.^25−27^ For example, when a typical AIE chromophore, tetraphenylethene (TPE), was connected via boronate esters, a highly blue-emissive 2D COF was produced.^25^ However, the connection of TPE by imine bonds resulted in complete quenching of the solid-state PL.^28^ This was ascribed to nonradiative energy dissipation caused by bond rotation^29^ or intramolecular electron transfer.^24^ Other strategies toward imine-bonded 2D COFs with high PL intensity, e.g., by restricting bond rotation with hydrogen bonds or controlling the layer stacking mode,^23,30^ have thus far had only limited success. Therefore, an efficient strategy for synthesizing luminescent imine-bonded 2D COFs remains elusive.

To date, aromatic dialdehydes or diamines have predominantly been used as *C*_2_-symmetric linkers for establishing highly crystalline imine-bonded 2D COFs. In sharp contrast, the use of aliphatic linkers has rarely been explored.^31−34^ Herein, we conceived a strategy to enhance the PL of 2D COFs through the introduction of cyclohexane as a linker, instead of the commonly used aromatic linkers, to suppress both intralayer π-conjugation and interlayer π–π interactions.

By polymerizing *trans*-1,4-diaminocyclohexane (CHDA) with different aromatic tri- or tetra-aldehydes, all the well-known lattice topologies of imine-bonded 2D COFs could be achieved. Both experimentally recorded powder X-ray diffraction (PXRD) patterns and theoretical calculations revealed that the interlayer distances of these cyclohexane-linked 2D COFs were unexpectedly large. Notably, these 2D COFs were highly luminescent, showing tunable emission colors, depending on the building blocks employed. In particular, incorporation of the TPE units into the cyclohexane-linked 2D COF led to a record photoluminescence quantum yield (PLQY) of 57% in the solid state. Unlike most imine-bonded COFs, the cyclohexane-linked COFs also allowed the retention of emission under acidic conditions. As a result, we achieved the trace detection of (i) dangerous explosives, (ii) essential metal elements in the metabolic process, and (iii) a biomarker, phenyl glyoxylic acid, demonstrating the promise of such COFs for sensing applications.

## Results and Discussion

### Design and Synthesis of COFs

**Figure 1:**
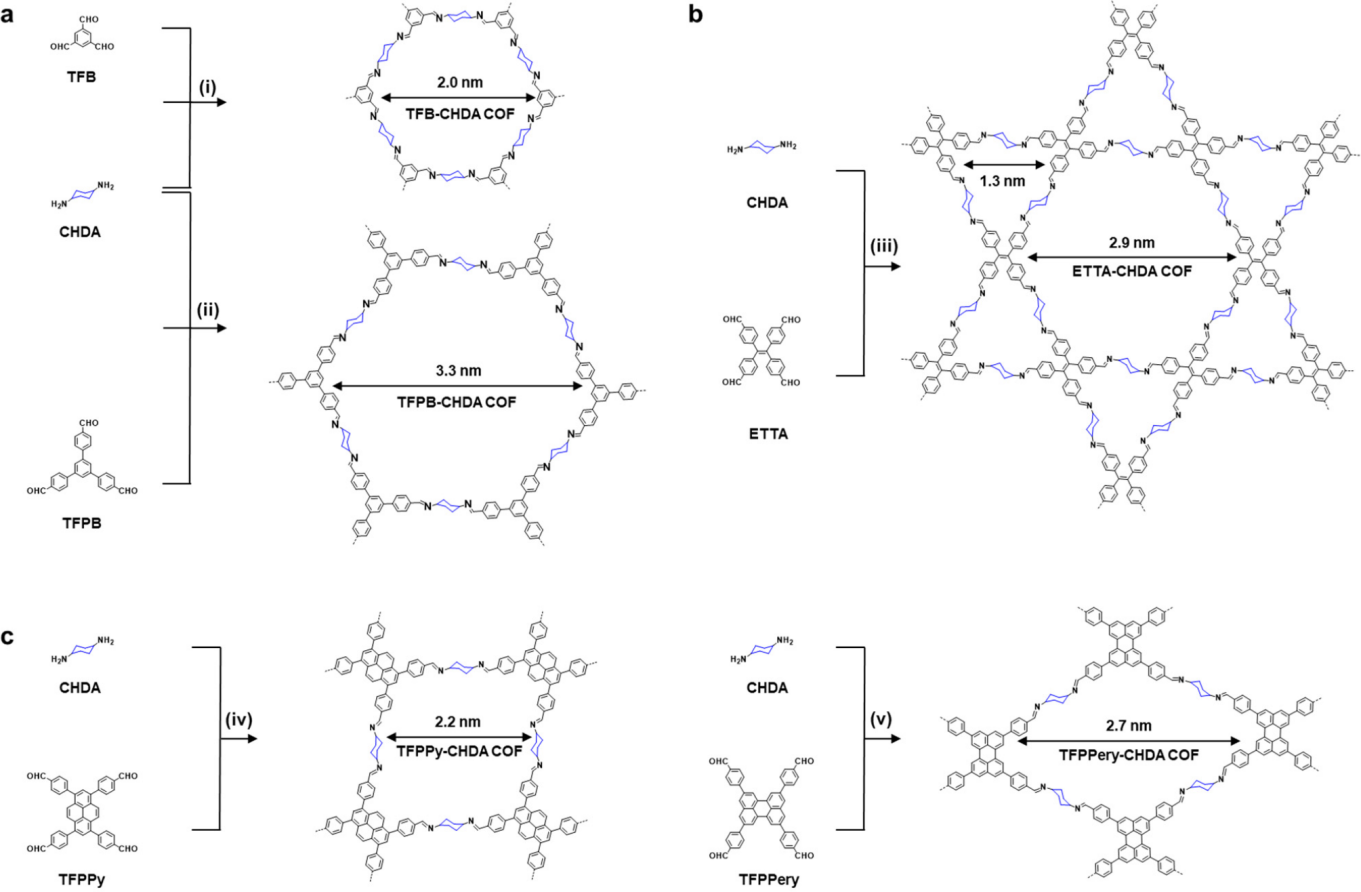
Synthesis of cyclohexane-linked 2D COFs. (a) Class 1: 2D TFB-CHDA COF and TFPB-CHDA COF with hcb topology. (b) Class 2: 2D ETTA-CHDA COF with kgm topology. (c) Class 3: 2D pyrene-based TFPPy-CHDA COF and perylene-based TFPPery-CHDA COF with sql topology. Solvothermal conditions: (i) acetic acid (HAc; 6 M), *N*,*N*-dimethylacetamide (DMAc)/1,4-dioxane (4/1), 120 °C, 72 h, 87%; (ii) HAc (6 M), 1-butanol, 120 °C, 72 h, 91%; (iii) HAc (6 M), DMAc/1,4-dioxane (4/1), 90 °C, 72 h, 82%; (iv) HAc (3 M), 1-butanol/1,4-dioxane (4/1), 120 °C, 72 h, 93%; (v) HAc (6 M), DMAc/1,4-dioxane (4/1), 120 °C, 72 h, 91%.

**Figure 2:**
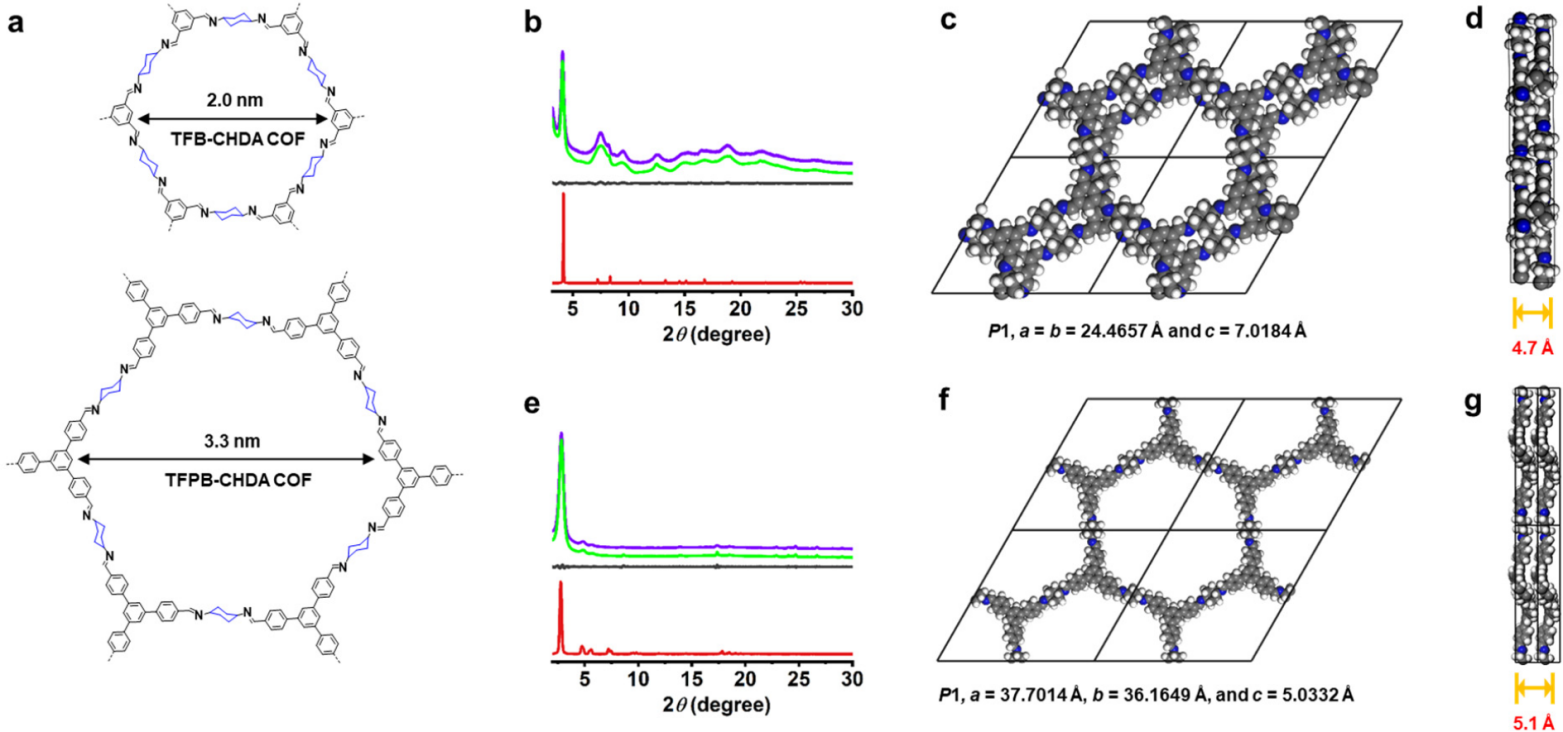
(a) Chemical structures of the TFB-CHDA COF and TFPB-CHDA COF. (b, e) Experimental PXRD (green line) and corresponding Pawley-refined patterns (purple line), simulated pattern from the AA-stacking mode (red line), and their difference (gray line) of (b) TFB-CHDA COF (*R*_p_ = 0.75%, *R*_wp_ = 1.56%) and (e) TFPB-CHDA COF (*R*_p_ = 1.94%, *R*_wp_ = 3.72%). (c, f) DFTB-optimized crystal structure viewed along the pseudoquadratic pore of (c) TFB-CHDA COF and (f) TFPB-CHDA COF. (d, g) DFTB-optimized crystal structure in the side view of (d) TFB-CHDA COF and (g) TFPB-CHDA COF.

**Figure 3:**
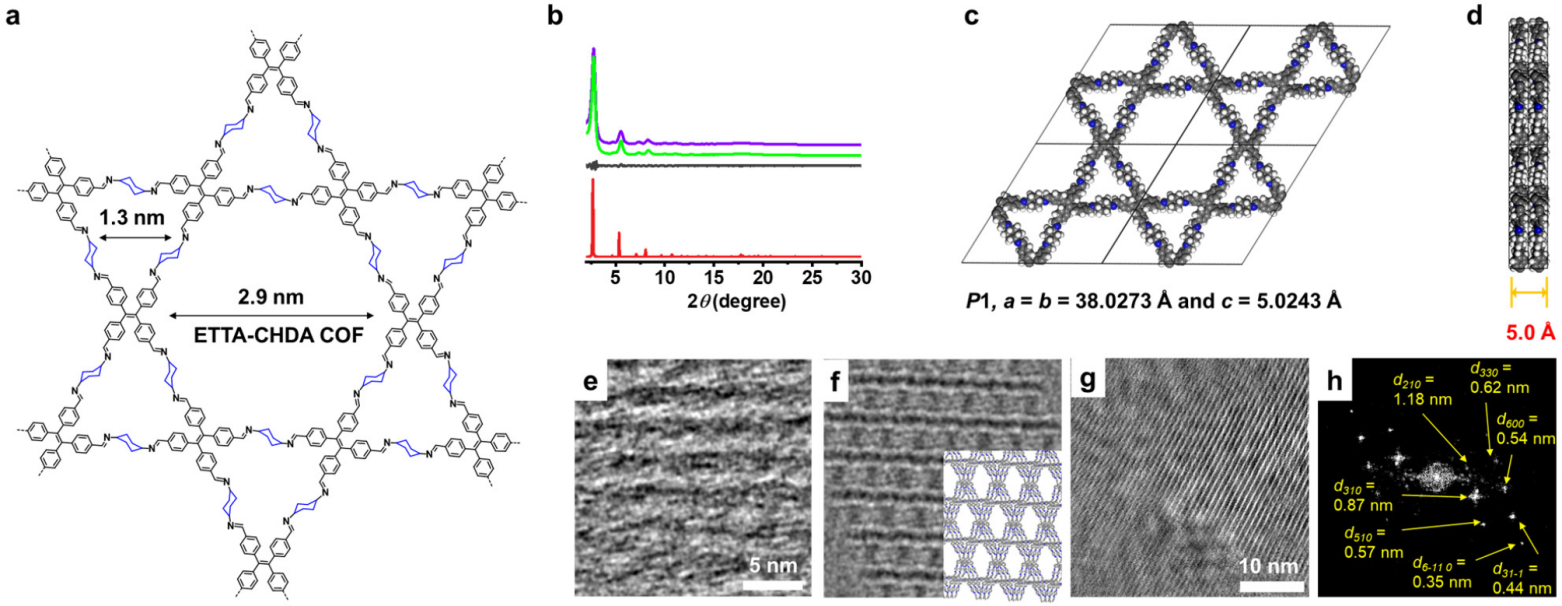
(a) Chemical structure of ETTA-CHDA COF. (b) Experimental PXRD (green line) and corresponding Pawley-refined (purple line) pattern (*R*_p_ = 2.43%, *R*_wp_ = 3.14%), simulated pattern from the eclipsed AA-stacking mode (red line), and their difference (gray line) between experimental PXRD and Pawley-refined pattern of ETTA-CHDA COF. (c) DFTB-optimized crystal structure viewed along the pseudoquadratic pore of ETTA-CHDA COF. (d) DFTB optimized crystal structure from a side view of ETTA-CHDA COF. (e) HR-TEM image of ETTA-CHDA COF showing a periodic structure. (f) A simulated TEM image generated from the molecular model of ETTA-CHDA COF shown in (c) and (d). The molecular model is partially overlaid to show the direction of the ETTA-CHDA COF in the simulated image. (g) HR-TEM image of ETTA-CHDA COF showing a periodic structure. (h) FFT image of (g).

**Figure 4:**
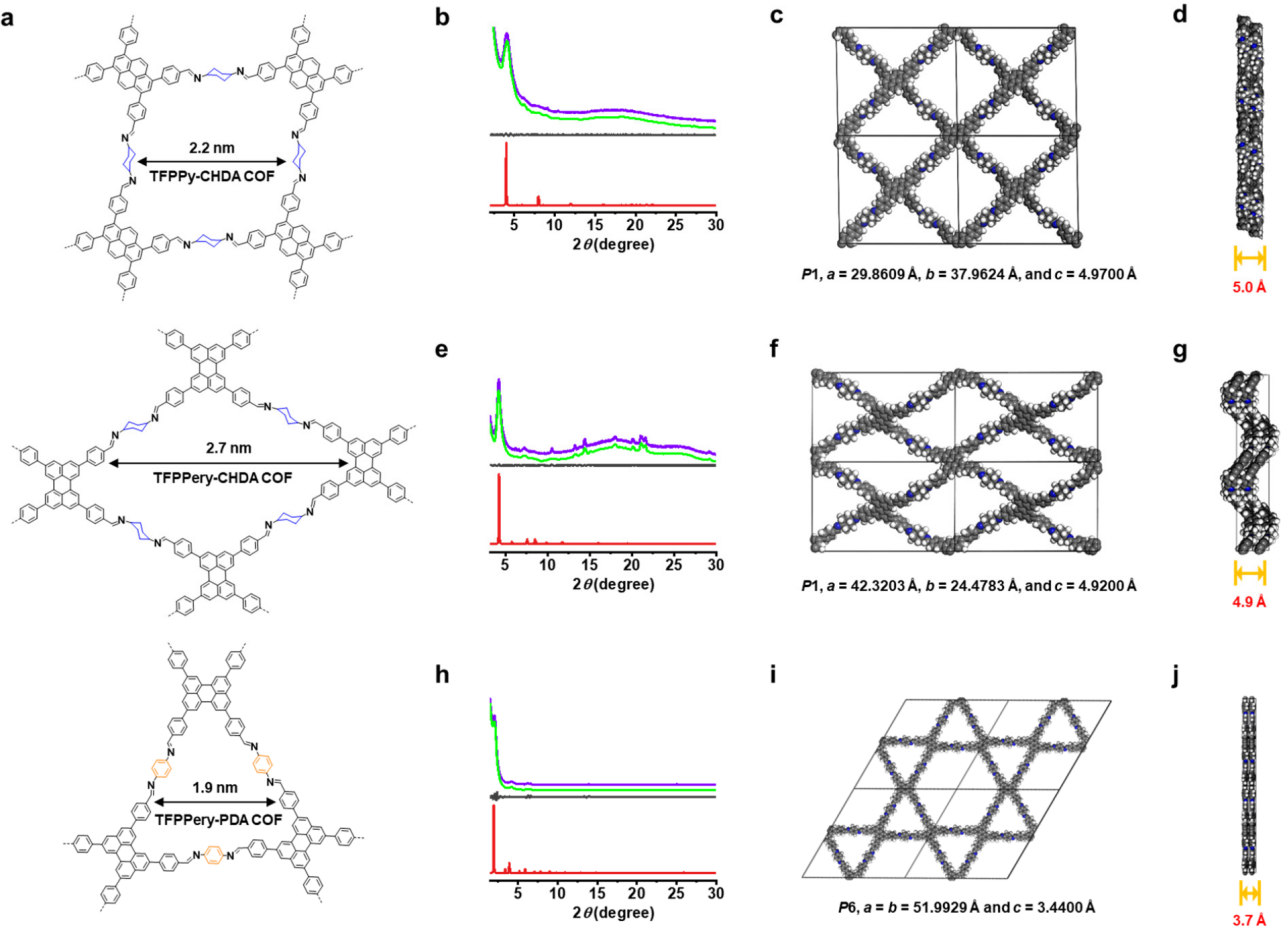
(a) Chemical structures of TFPPy-CHDA COF, TFPPery-CHDA COF, and TFPPery-PDA COF. (b, e, h) Experimental PXRD (green line) and corresponding Pawley-refined (purple line) pattern, simulated pattern from the eclipsed AA-stacking mode (red line), and their difference (gray line) of (b) TFPPy-CHDA COF (*R*_p_ = 0.65%, *R*_wp_ = 0.99%), (e) TFPPery-CHDA COF (*R*_p_ = 0.66%, *R*_wp_ = 1.36%), and (h) TFPPery-PDA COF (*R*_p_ = 1.14%, *R*_wp_ = 2.79%). (c, f, i) DFTB-optimized crystal structure viewed along the pseudoquadratic pore of (c) TFPPy-CHDA COF, (f) TFPPery-CHDA COF, and (i) TFPPery-PDA COF. (d, g, j) DFTB-optimized crystal structure from a side view of (d) TFPPy-CHDA COF, (g) TFPPery-CHDA COF, and (j) TFPPery-PDA COF.

The synthesis of varying cyclohexane-linked COFs was designed through a general reaction strategy based on the copolymerization of CHDA and various aromatic tri- or tetra-aldehydes. The solvothermal conditions were carefully optimized to obtain these cyclohexane-linked COFs with high crystallinity and yields. The most decisive parameter for obtaining highly crystalline COFs with CHDA was the amount of acetic acid (HAc) used as the catalyst. This is presumably because excess HAc can protonate the amino groups of CHDA,^35^ thus hindering the formation of an ordered framework (Table S1). The polycondensation of different aromatic tri- and tetra-aldehydes with CHDA allowed the formation of cyclohexane-linked 2D COFs with three key lattice topologies: honeycomb (hcb), Kagome (kgm), and square lattice (sql) (see below for the characterization details). The resulting COFs were categorized into three classes depending on the lattice topology (Figure 1, Schemes S1–S5):

Class 1: TFB-CHDA COF and TFPB-CHDA COF with hcb topology (Figure 2a), prepared with 1,3,5-triformylbenzene (TFB) and 1,3,5-tris(4′-formylphenyl)benzene (TFPB), respectively;

Class 2: ETTA-CHDA COF with kgm topology (Figure 3a), prepared with 4,4′,4″,4‴-(ethene-1,1,2,2-tetrayl)tetrabenzaldehyde (ETTA);

Class 3: TFPPy-CHDA and TFPPery-CHDA COF with sql topology (Figure 4a), prepared with 1,3,6,8-tetrakis(4-formylphenyl)pyrene (TFPPy) and 2,5,8,11-tetrakis(4-formylphenyl)perylene (TFPPery), respectively. For comparison, the TFPPery-PDA COF was also synthesized through condensation of TFPPery and *p*-phenylenediamine (PDA) (Scheme S6).

### Structural Characterization

The obtained COFs were thoroughly characterized by utilizing elemental analysis (EA), Fourier transform infrared (FT-IR) spectroscopy, powder X-ray diffraction (PXRD), nitrogen sorption isotherms, field emission scanning electron microscopy (FE-SEM), thermogravimetric analysis (TGA), ^13^C cross-polarization magic-angle spinning nuclear magnetic resonance (CP-MS NMR) spectroscopy, and high-resolution transmission electron microscopy (HR-TEM). The FT-IR spectra of all the obtained COF samples revealed the characteristic imine (C=N) stretching bands in the range of 1639–1641 cm^–1^, corroborating the formation of imine linkages, and the greatly attenuated peaks in the range of 2720 cm^–1^ attributed to the aldehyde groups (Figures S1–S5). Moreover, the ^13^C CP-MAS NMR spectra showed a signal at 158 ppm (Figure S6), providing further evidence of the formation of imine bonds.^36^

The PXRD analyses of TFB-CHDA COF (Figure 2b), TFPB-CHDA COF (Figure 2e), ETTA-CHDA COF (Figure 3b), TFPPy-CHDA COF (Figure 4b), TFPPery-CHDA COF (Figure 4e), and TFPPery-PDA COF (Figure 4h) exhibited intense peaks, indicating their high crystallinity (Table S2). This is in contrast to a previous report by Zhou et al., who attempted to synthesize the TFB-CHDA COF but only obtained powders with undesired crystallinity,^37^ demonstrating the importance of optimizing solvothermal conditions. Pawley refinement and lattice modeling (Materials Studio, version 4.4) resulted in optimized parameters. The refined PXRD patterns matched well with the experimental profiles (Tables S3–S8). A comparison of the observed and simulated PXRD patterns suggested slipped AA stacking for the TFB-CHDA COF (Figure 2c) and eclipsed AA stacking for the TFPB-CHDA COF, ETTA-CHDA COF, TFPPy-CHDA COF, TFPPery-CHDA COF, and TFPPery-PDA COF (Figures 2f, 3c, 4c, 4f, and 4i). In contrast, their staggered AB modes (blue curves) did not agree with the experimental results (Figures S7–S12).

Notably, while the reported benzene-based 2D COF-LZU1 (TFB-PDA), constructed from TFB and PDA, exhibited eclipsed stacking with a layer distance of 3.7 Å,^38^ TFB-CHDA COF revealed an increased layer distance of approximately 4.7 Å. The skewing and separation of the COF layers can be ascribed to the steric repulsions at the axial hydrogens of the cyclohexane units. Similarly, TFPB-CHDA COF showed a large layer distance of 5.1 Å compared to the 3.5 Å reported for the corresponding phenylene-linked TFPB-PDA COF (NUS-15).^39^ ETTA-CHDA COF, TFPPy-CHDA COF, and TFPPery-CHDA COF also demonstrated large layer distances of 4.9–5.0 Å.

Nitrogen adsorption–desorption experiments at 77 K were carried out to measure the porosity of the COF samples, and all of them revealed a reversible type-I isotherm with a slight hysteresis, characteristic of a mesoporous structure (Figures S13–S18). The Brunauer–Emmett–Teller (BET) surface areas of TFB-CHDA and TFPB-CHDA COFs were analyzed to be 1177 and 478 m^2^ g^–1^, respectively (Figures S13a and S14a). Quenched solid density functional theory (QSDFT) calculations based on the nitrogen adsorption curve furnished pore sizes centered at 1.8 nm for the TFB-CHDA COF and 3.8 nm for the TFPB-CHDA COF, which agreed with the pore size of every single layer predicted from their crystal structures (Figures S13b and S14b). On the other hand, measurements of the activated ETTA-CHDA COF indicated a larger BET surface area of 1637 m^2^ g^–1^ (Figure S15a). The QSDFT simulation indicated that the pore sizes of ETTA-CHDA COF were 1.5 and 2.8 nm, consistent with the predicted pore sizes of 1.3 nm for micropores and 2.9 nm for mesopores from the theoretical model constructed with Materials Studio (Figure 3 and Figure S15b).^40^ The activated TFPPy-CHDA COF, TFPPery-CHDA COF, and TFPPery-PDA COF displayed BET surface areas of 620, 559, and 176 m^2^ g^–1^, respectively (Figures S16a, S17a, and S18a). Their pore size distributions were derived from nitrogen absorption curves and QSDFT calculations, and the pore sizes of TFPPy-CHDA and TFPPery-CHDA COF were shown to be only approximately 2.2 nm (Figure S16b) and 2.7 nm (Figure S17b), respectively. This is in contrast to the TFPPery-PDA COF, for which two types of pore sizes centered at 1.9 and 3.7 nm were revealed (Figure S18b), in agreement with a previous report by Bein et al.^41^

The BET surface area of the TFPPery-CHDA COF was much larger than that of the TFPPery-PDA COF, which was attributed to the presence of more structural defects in the latter. The limited solubility of large π-conjugated organic chromophores as building blocks can often account for the failure to obtain highly ordered COF structures.^42^ We assumed that the use of CHDA enhanced the solubility and conformational flexibility of intermediate structures during COF formation, facilitating the construction of highly ordered frameworks even with large π-conjugated units based on pyrene and perylene. On the other hand, TFPB-CHDA COF maintained its crystalline lattice after a normal purification process, presumably due to enhanced conformational flexibility. This stands in contrast to some previously reported COFs,^35,36^ including TFPB-PDA COF, which lost their crystallinity due to the collapse of porous structures after washing with tetrahydrofuran (THF)/acetone and drying under vacuum.

As seen by FE-SEM, TFB-CHDA COF and TFPB-CHDA COF appeared as aggregated submicrometer-sized granular crystallites (Figures S19 and S20), while ETTA-CHDA COF, TFPPy-CHDA COF, TFPPery-PDA COF, and TFPPery-CHDA COF all showed a rodlike morphology (Figures S21–S23) consisting of microcrystals. In addition, TGA revealed the exceptional thermal stability of the obtained COF samples, namely, TFB-CHDA COF and TFPB-CHDA COF up to 400 °C, ETTA-CHDA COF up to 460 °C, and TFPPy-CHDA COF and TFPPery-CHDA COF up to 550 °C under a nitrogen atmosphere (>80% retention, Figures S24–S28). Moreover, all of these COFs also showed stability up to 300 °C under air based on the TGA analysis (Figure S29).

Notably, HR-TEM images of the exfoliated film of ETTA-CHDA COF showed a stripe pattern with ∼3.0 nm intervals, which corresponded to the distance between two parallel edges around the mesopore, validating its crystalline Kagome-type lattice structure with AA-type stacking (Figure 3e and 3f). Moreover, low-electron-dose TEM measurements showed lattice fringes, revealing nine different *d*-spacings (Figures 3g, 3h, S30, and S31). While the *d*-spacings of 1.18, 0.87, 0.64, and 0.49 nm matched well with the peaks in the experimental PXRD, the other five values did not correspond to any of the experimentally observed PXRD peaks. However, the simulation from the molecular model after Pawley refinement showed the diffraction peaks corresponding to these five *d*-spacings (Figure S31f), providing additional structural support for ETTA-CHDA COF. We assumed that these five peaks were broadened and nondetectable in the experimental PXRD, presumably due to the small crystal domain sizes of the obtained COFs. On the other hand, HR-TEM images of highly ordered TFPPery-CHDA COF fully supported its successful formation with the tetragonal topology (Figure S32). Additionally, a honeycomb pattern was observed for the TFB-CHDA COF, in agreement with the formation of a hexagonal lattice with slipped AA stacking (Figure S33).

### Influence of Cyclohexane Units on the Network Topology of COFs

The introduction of the cyclohexane units appeared to exert a major influence on the resulting topology. For instance, Bein et al. reported a series of perylene-linked 2D COFs in 2019,^41^ revealing a kgm topology with dual pores, and we reproduced this result with the TFPPery-PDA COF in this work. In contrast, the replacement of the phenylene with cyclohexane linkers led to the sql topology as in TFPPery-CHDA COF. To further elucidate the mechanism of topology control by cyclohexane linkers, their packing energies were calculated by a reported density functional theory (DFT)-based method.^23^ The total crystal stacking energy of the tetragonal TFPPery-CHDA COF was calculated to be 4.8 kcal mol^–1^ higher than that of the Kagome topology. The sql topology is thus energetically favored. In sharp contrast, the total stabilization energy of the Kagome-type TFPPery-PDA COF is 2.1 kcal mol^–1^ higher than that of the tetragonal lattice, supporting the optimized packing structure of the Kagome-type (Table S9). This observation provides guidance for the facile modulation of the topology and pore size of the target 2D COFs for various applications, in particular, sensing of different analytes.

### Optoelectronic Properties

**Figure 5:**
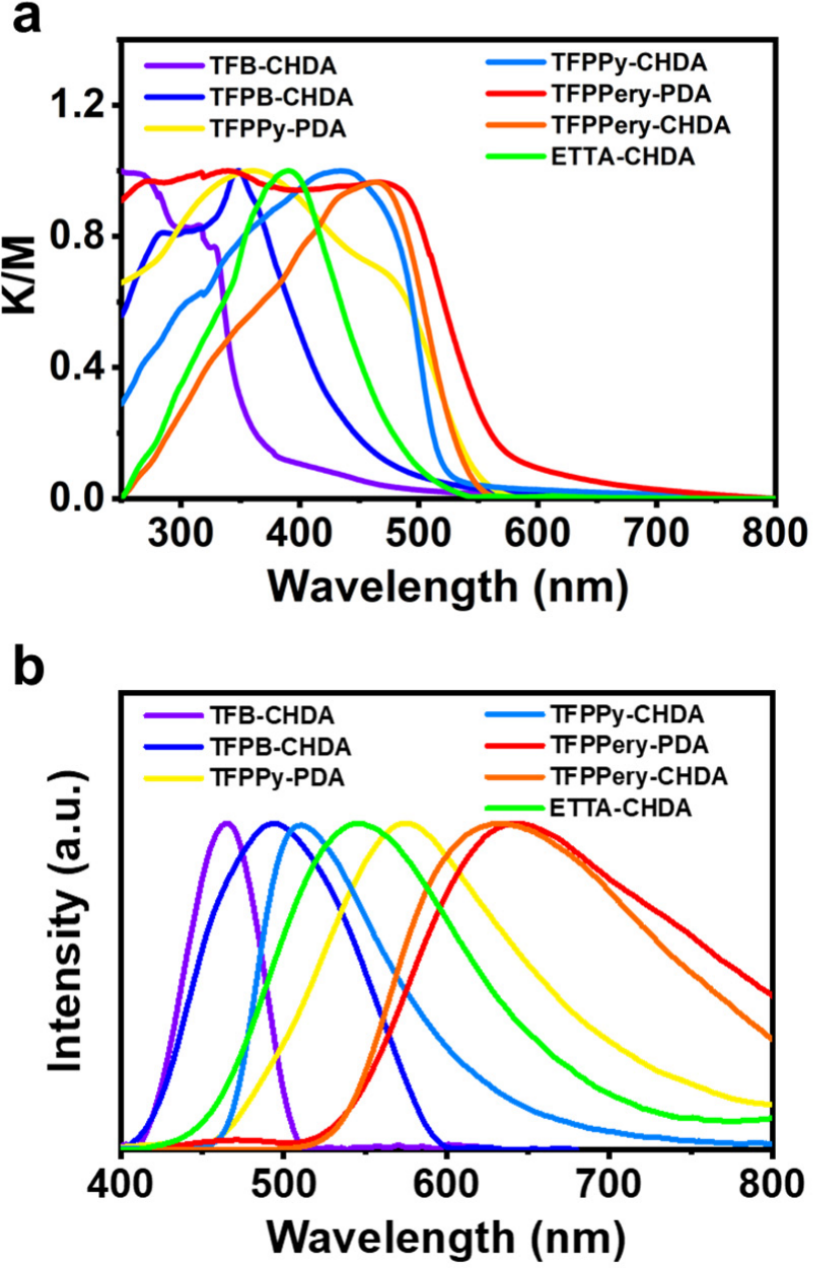
(a) Normalized UV–vis DRS and (b) luminescence spectra of TFB-CHDA COF, TFPB-CHDA COF, TFPPy-PDA COF, TFPPy-CHDA COF, TFPPery-PDA COF, TFPPery-CHDA COF, and ETTA-CHDA COF powders.

UV–vis diffuse reflectance spectroscopy (DRS) was performed on the powder samples of the obtained COFs as well as their monomer precursors to study their optical properties (Figures 5a and S34). The spectra of TFB-CHDA COF, TFPB-CHDA COF, and ETTA-CHDA COF present absorption edges at 358, 442, and 490 nm, respectively, which are close to those of the corresponding TFB, TFPB, and ETTA monomers (Figure S34), signifying the interrupted π-conjugation within the COF layers. The absorption edges of TFPPy-CHDA COF and TFPPery-CHDA COF are red-shifted to 515 and 541 nm, respectively, reflecting the planar π-conjugated structures of the TFPPy and TFPPery units. By comparison, TFPPy-PDA^27^ and TFPPery-PDA COF with phenylene linkers instead of cyclohexane linkers exhibit much broader and red-shifted absorption, especially in the case of the former, presumably reflecting their less ordered structures and the further extension of the π-conjugation as well as interlayer interactions.

The emission spectra of TFB-CHDA COF, TFPB-CHDA COF, ETTA-CHDA COF, TFPPy-CHDA COF, and TFPPery-CHDA COF display maxima at 465, 494, 544, 510, and 633 nm, respectively (Figure 5b), corresponding to colors from blue to orange-red (Figure S35). We also measured the solid-state emission spectra of the building blocks of TFB, TFPB, ETTA, TFPPy, and TFPPery (Figure S36). A comparison of the emission spectra of TFPPy-CHDA COF, TFPPy-PDA COF, and tri/tetra-aldehyde monomers demonstrates that insertion of the saturated cyclohexane units can suppress the red-shift of the emission wavelength of the organic chromophore upon integration into the COF structure. This feature is advantageous for avoiding color impurities for applications as light-emitting materials, especially for blue-color emission.

Remarkably, the cyclohexane-linked 2D COFs demonstrated high PLQYs of 4% (TFB-CHDA COF), 7% (TFPB-CHDA COF), 23% (TFPPy-CHDA COF), 33% (TFPPery-CHDA COF), and 57% (ETTA-CHDA COF) in the solid state (Table 1; see the SI for details). The fluorescence micrographs of ETTA-CHDA COF as a representative case with the highest PLQY revealed a beltlike morphology, exhibiting bright green emission across the whole belt (Figure S37). The PL decay times of these COFs were measured, revealing PL lifetimes in the range of 5.1–5.9 ns (Table 1), indicating that this series of cyclohexane-linked COFs can maintain the photoexcited singlet state for a similar period of time.

**Table 1:** Optoelectronic Properties of Cyclohexane-Based 2D Imine-Linked COFs

topology	COFs	λ_edg_ (nm)	λ_em_ (nm)	Φ	τ (ns)	VBM (eV)^a^	CBM (eV)^a^
hcb	TFB-CHDA COF	358	465	4%	5.8	–5.94	–2.73
	TFPB-CHDA COF	442	494	7%	5.9	–5.84	–2.70
kgm	ETTA-CHDA COF	490	544	57%	5.1	–5.64	–3.11
	TFPPery-PDA COF	579	643	0.1%	3.0	–5.08	–3.52
sql	TFPPy-CHDA COF	515	510	23%	5.6	–5.41	–3.37
	TFPPery-CHDA COF	541	633	33%	5.5	–5.30	–3.43
	TFPPy-PDA COF	549	576	0.2%	2.7	–5.46	–3.86

a DFT calculations were performed by means of AMS-DFTB software.^43^

### Role of Cyclohexane in the Luminescence of 2D COFs

In contrast to the TFPPy-PDA and TFPPery-PDA COF, which show only very small PLQYs below 0.3% (Table 1), the cyclohexane-linked TFPPy-CHDA and TFPPery-CHDA COFs display remarkably higher PLQYs of 23% and 33%, respectively, in the solid state. These results indicate the major role of the cyclohexane linkers in enhancing the solid-state PL performance in TFPPy- and TFPPery-based COFs. The interlayer distances of both TFPPy-CHDA COF and TFPPery-CHDA COF are indeed as large as ∼5 Å, which was ascribed to the significant steric repulsion induced by the cyclohexane units and account for the suppression of the ACQ through the interlayer interactions. On the other hand, the nonradiative energy dissipation caused by the bond rotation is also discussed as a possible reason for the PL quenching of 2D COFs.^29^ Since cyclohexane linkers are considered to be able to still rotate in TFPPy-CHDA COF and TFPPery-CHDA COF,^29^ we concluded that the effect of ACQ in PL quenching predominates over that of bond rotation. For ETTA-CHDA COF with AIE-active TPE units, the increase in the layer distance may be less important for enhancing the PL performance. The record-high PLQY of 57% with this COF thus suggests the suppression of photoinduced charge transfer upon replacement of the phenylene with a cyclohexane linker along with PL enhancement through the AIE effect of TPE units. The sharp contrast to AIE-active TPE-based ETTA-PDA COF with a low PLQY of 0.6% further supports our conclusion and confirms the unique role of interrupted conjugation in achieving outstanding emission.

### Sensing Performance

**Figure 6:**
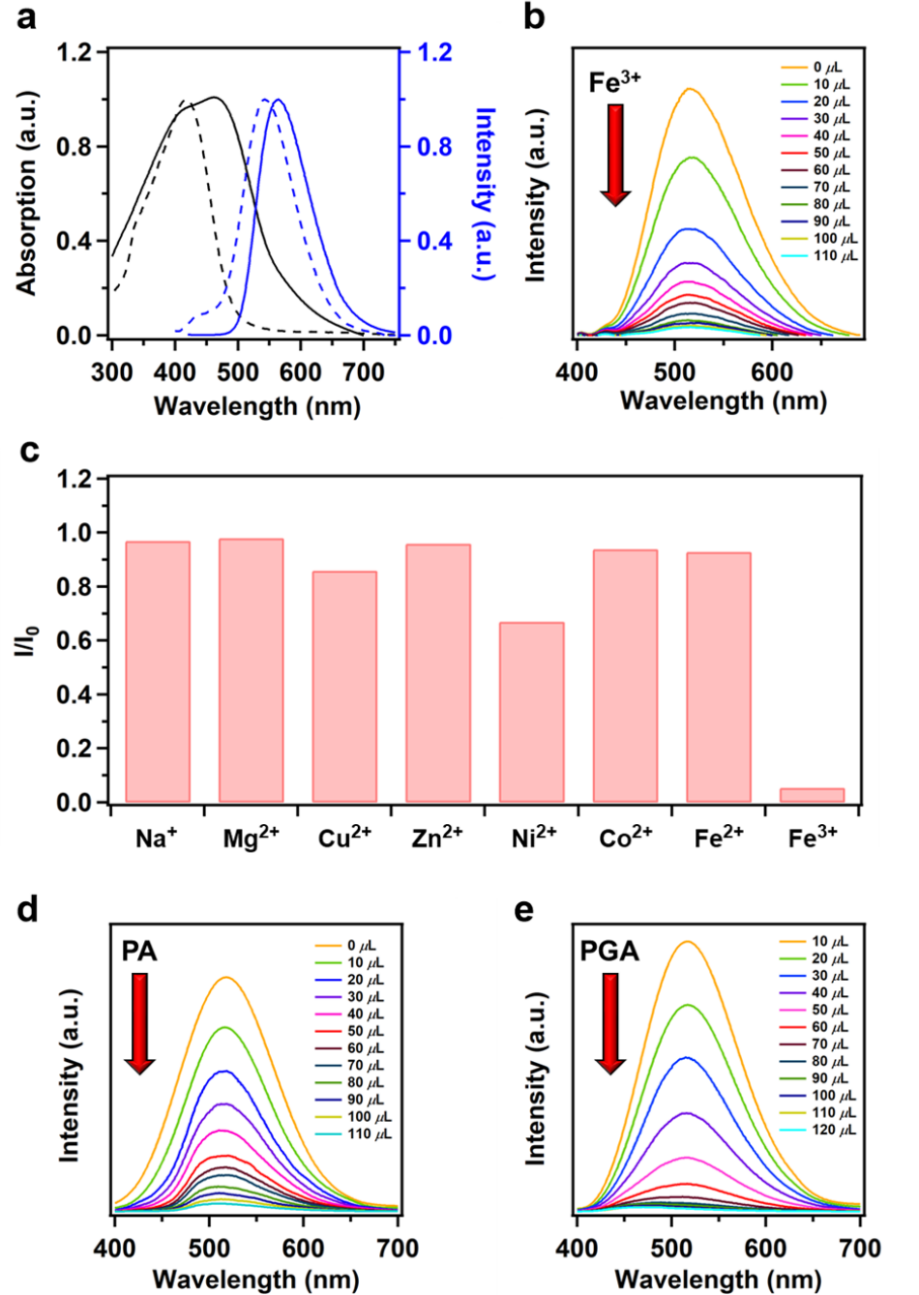
(a) UV–vis DRS (black) and PL (blue) spectra of pristine (dashed line) and acidified (solid line) ETTA-CHDA COF in the solid state. (b) Emission spectra of the ETTA-CHDA COF THF solution (*c* = 50 μg mL^–1^) upon titration with an Fe^3+^ solution (0.03 M in THF) at room temperature (λ_ex_ = 360 nm). (c) Luminescence response of ETTA-CHDA COF in the presence of different metal ions (0.03 M) in THF (λ_ex_ = 360 nm). (d) PL spectra of an ETTA-CHDA COF THF solution (*c* = 50 μg mL^–1^) upon titration with a PA solution (6 × 10^–4^ M in THF) at room temperature (λ_ex_ = 360 nm). (e) PL spectra of an ETTA-CHDA COF THF solution (*c* = 50 μg mL^–1^) upon titration with a PGA solution (5 × 10^–4^ M in THF) at room temperature (λ_ex_ = 360 nm).

The luminescence of imine-bonded COFs can be readily quenched by acids, which greatly limits their applicability for sensing in acidic environments.^44^ To our delight, the PL signal of ETTA-CHDA COF was not quenched upon the addition of trifluoroacetic acid (TFA) but only showed red-shifts of the absorption and emission bands both in the solid state (45 and 23 nm, respectively) and in THF solution (61 and 43 nm, respectively) (Figures 6a and S38). The response to acid was found to be reversible in the cycling test, demonstrating full recovery upon the addition of triethylamine (TEA) (Figure S39). Swift protonation might occur since the nitrogen atoms of the imine bonds in cyclohexane-incorporating COFs are more basic than those in COFs with aromatic linkers and improve the stability under acidic conditions through the formation of hydrogen bonds.^45^ Moreover, the PL from the TPE units as independent emitters is not significantly affected, in sharp contrast to most of the reported imine-bonded COFs,^46^ highlighting the potential of ETTA-CHDA COF for practical applications.

To assess the sensing performance of ETTA-CHDA COF, we examined the detection of Fe^3+^ ions, which are known to be essential in many biological processes. ETTA-CHDA COF gave a brilliant luminescence with a PLQY of 23% in THF dispersion. The luminescence intensity of the ETTA-CHDA COF solution drastically decreased with increasing concentrations of Fe^3+^ ions (Figure 6b). When a THF dispersion of ETTA-CHDA COF was mixed with 70 μL of Fe^3+^, the degree of PL quenching exceeded 90%. The final Stern–Volmer quenching rate constant (*K*_sv_) was calculated to be 2.00 × 10^4^ M^–1^ (Figure S40), with a low limit of detection (LOD) of 680 ppb, indicating the high sensing performance of ETTA-CHDA COF. We further investigated the scope of sensing other ion species (Figures 6c and S41). Compared to Fe^3+^ ions, ETTA-CHDA COF exhibited relatively low sensitivity to other transition metal ions and alkali metal ions. For instance, the PL intensity was quenched by only 33% and 14%, respectively, upon the addition of Ni^2+^ and Cu^2+^. Moreover, the sensitivity of ETTA-CHDA COF to Co^2+^, Fe^2+^, Na^+^, and Mg^2+^ was negligible, showing only less than 5% change in the PL intensity.

These results highlight the high selectivity of ETTA-CHDA COF for Fe^3+^ detection. Notably, ETTA-CHDA COF shows a negligible response to Fe^2+^, allowing one to precisely distinguish Fe^3+^ from Fe^2+^ ions. The pronounced luminescence quenching of ETTA-CHDA COF for Fe^3+^ might be attributable to the energy transfer from ETTA-CHDA COF to Fe^3+^.^47^ In water, ETTA-CHDA COF could be only suspended without showing any color of dispersion but showed the PL quenching of 17% upon addition of 120 μL of aqueous Fe^3+^ solution (Figure S42). ETTA-CHDA COF and possibly other cyclohexane-based COFs with appropriate energy levels are thus promising for real-time biosensing of specific metal ion species.

Remarkably, ETTA-CHDA COF also exhibited prominent PL turn-off responses to explosive and toxic picric acid (PA). For the recognition of PA, the PL intensity of ETTA-CHDA COF in a THF solution was quenched by up to 98% with increasing concentrations of PA (Figure 6d). The overall *K*_sv_ was estimated to be 3.04 × 10^5^ M^–1^ (Figure S43) with a detection limit of 188 ppb. The reversibility of the luminescence response toward PA was also established. To the best of our knowledge, this *K*_sv_ value is among the highest ones reported for porous materials.^48^ The high PA sensitivity of ETTA-CHDA COF is probably attributable to its brilliant luminescence and large BET surface area. In addition, the large 1D open nanochannels may also provide pathways for PA molecules to efficiently interact with the TPE units inside the framework, causing PL quenching through photoinduced energy transfer.^49^

As a human urine metabolite of ethylbenzene and styrene (EB/S), phenyl glyoxylic acid (PGA) can indicate the amount of EB/S absorbed by the human body through daily contact with plastic pollutants,^50^ making it important to develop sensitive sensors for PGA. Remarkably, ETTA-CHDA COF was also capable of detecting traces of PGA, with PL quenching of up to 99% upon addition of PGA to the THF solution (Figure 6e). The ETTA-CHDA COF, when applied to a human physiological urine system, reaches a low limit of detection of 134 ppb^51^ and a calculated *K*_sv_ of 2.83 × 10^5^ M^–1^ (Figure S44). Since the LUMO energy level of ETTA-CHDA COF (−3.11 eV, Table 1) was lower than that of PGA (−2.44 eV), photoinduced electron transfer was not feasible during the PGA recognition process.^52^ Therefore, the formation of nonluminescent complexes was probably the main reason for PL quenching.^53^ Additionally, the PXRD patterns (Figure S45) and SEM images (Figure S46) of ETTA-CHDA COF before and after sensing did not show any noticeable difference, indicating the high stability of the COF under practical conditions. ETTA-CHDA COF can thus provide an effective platform for sensing trace amounts of PGA for analyzing the EB/S content in the human body.

## Conclusion

Efficient design concepts for luminescent imine-bonded 2D COFs have remained challenging despite great research efforts. In this work, we introduced a series of cyclohexane-linked 2D COFs with remarkably high solid-state photoluminescence. The cyclohexane linkers restrict the π-conjugation within the COF layers and enlarge the interlayer distances to approximately 5 Å, thus suppressing PL quenching through intra- and interlayer interactions. In particular, ETTA-CHDA COF with AIE-active TPE units demonstrates a record-high solid-state PLQY of 57%. This cyclohexane-linked COF also exhibits outstanding sensitivity and selectivity for the recognition of Fe^3+^ ions, explosive and toxic PA, and PGA as urine metabolites (Table S10). Distinct from most of the other reported COF systems that require complicated syntheses of building blocks, this approach can readily provide brilliant imine-bonded 2D COFs with controllable topologies and pore sizes via polycondensation of organic chromophores having formyl groups and commercially available CHDA. Many more cyclohexane-linked COFs are now within reach, paving the way toward improved sensing, imaging, and light-emitting devices.

## Supporting Information Available

Experimental procedures, characterization data for all new compound, and materials of COFs (PDF)
