# Supplementary material for: Saturated Linkers in Two-Dimensional Covalent Organic Frameworks Boost Their Luminescence
Source: J Am Chem Soc. 2023 Jun 20;145(26):14417–26. doi: 10.1021/jacs.3c03614 (PMC10326870; doi:10.1021/jacs.3c03614)
Supplement: Supplementary file 1 — ja3c03614_si_001.pdf [file ja3c03614_si_001.pdf]

# Saturated Linkers in Two-Dimensional Covalent Organic Frameworks Boost their Luminescence

Meijia Yang, Hiroki Hanayama, Long Fang, Matthew A. Addicoat, Yunyu Guo, Robert Graf, Koji Harano, Jun Kikkawa, Enquan Jin*, Akimitsu Narita* and Klaus Müllen*

Dr. M. Yang, Dr. R. Graf, Prof. Dr. E. Jin, Prof. Dr. A. Narita and Prof. Dr. K. Müllen
Max Planck Institute for Polymer Research
Ackermannweg 10, 55128, Mainz, Germany.
E-mail: muellen@mpip-mainz.mpg.de

Y. Guo and Prof. Dr. E. Jin
State Key Laboratory of Inorganic Synthesis and Preparative Chemistry,
College of Chemistry and International Center of Future Science, Jilin University
Changchun 130012, P.R. China.
E-mail: enquanjin@jlu.edu.cn;

Dr. H. Hanayama and Prof. Dr. A. Narita
Organic and Carbon Nanomaterials Unit, Okinawa Institute of Science and Technology Graduate University
Kunigami-gun, Okinawa 904-0495, Japan.
E-mail: akimitsu.narita@oist.jp

L. Fang
Key Laboratory for Polymeric Composite and Functional Materials of Ministry of Education, School of Chemistry, Sun Yat–sen University
Guangzhou 510275, Guangdong Province, China.

Prof. Dr. M. A. Addicoat
School of Science and Technology, Nottingham Trent University
Clifton Lane, Nottingham NG11 8NS, UK.

Dr. K. Harano and Dr. J. Kikkawa
Center for Basic Research on Materials, National Institute for Materials Science
1-1 Namiki, Tsukuba, Ibaraki 305-0044, Japan.

# Table of Contents

**Section A:** General Materials and Methods

**Section B:** Synthetic Details

**Section C:** Characteristics

**Section D:** Fluorescence Sensor Experiments

**Section E:** References

## **Section A:** General Materials

*N*,*N*-dimethylacetamide (DMAc), 1,4-dioxane, acetic acid (HAc), *n*-butanol (*n*-BuOH), 1,2-dichlorobenzene (*o*-DCB), *N*,*N*-dimethylformamide (DMF), dichloromethane (DCM) tetrahydrofuran (THF), triethylamine (TEA), meistylene, acetone and methanol were purchased from Sigma‐Aldrich company. 1,3,5-triformylbenzene (TFB), *p*-phenylenediamine (PDA), tetrakis(triphenylphosphine)palladium ($Pd(PPh_3)_4$), 4-bromobenzaldehyde, potassium carbonate ($K_2CO_3$), picric acid (PA) and phenylglyoxylic acid (PGA) were purchased from Tokyo chemical industry Co., Ltd. (TCI). $NaClO_4$, $Mg(ClO_4)_2$, $Cu(ClO_4)_2$, $Zn(ClO_4)_2$, $Ni(ClO_4)_2$, $Co(ClO_4)_2$, $Fe(ClO_4)_2$ and $Fe(ClO_4)_3$ were used for the detection of diverse metal ions. The 1,3,5-Tris(4-formylphenyl)benzene (TFPB),[1] 1,3,6,8-tetrakis(4-formylphenyl)pyrene (TFPPy),[2-7] 2,5,8,11-tetrakis(4,4,5,5-tetramethyl-1,3,2-dioxaborolan-2-yl)perylene[8], 2,5,8,11-tetrakis(4-formylphenyl)perylene (TFPPery),[9] and TFPPy-PDA COF[10] were synthesized according to reported methods. The 4,4',4'',4'''-(ethene-1,1,2,2-tetrayl)tetrabenzaldehyde (ETTA) was purchased from AbaChemScene company. Unless otherwise noted, all reagents were used without further purification. *Trans*-1,4-diaminocyclohexane (CHDA) was obtained from Tokyo chemical industry Co., Ltd. (TCI) and recrystallized by DCM and methanol for several times before it is applied.

## Characterization Methods

All reactions were performed under Ar atmosphere or with Schlenk line techniques. Thin layer chromatography (TLC) was carried out on silica gel-coated aluminum sheets with an F254 indicator, and column chromatography separation was conducted with silica gel (particle size of 0.063–0.200 mm). High-resolution mass spectra (HR-MS) were recorded on a Bruker Reflex II-TOF spectrometer by matrix-assisted laser decomposition/ionization (MALDI) using 7,7,8,8-tetracyanoquinodimethane (TCNQ) as matrix calibrated against poly(ethylene glycol). Nuclear magnetic resonance (NMR) spectra were recorded using Bruker DPX 300 MHz NMR spectrometers, and chemical shifts ($\delta$) were expressed in ppm relative to the residual of solvents ($C_2D_2Cl_4$, 1H: 6.00

ppm). Coupling constants (*J*) were expressed in Hertz. Fourier transform infrared (FT-IR) spectroscopy was detected on a Bruker TENSOR II FTIR spectrometer with a scan number of 64, and the background was subtracted. Each elemental analysis (EA) was obtained on Elementar Vario EL elemental analyzer. Powder X-ray diffraction (PXRD) measurements were performed on a Rigaku Smart Lab X-ray diffractometer by putting powder on the glass substrate, from $2\theta = 1.5°$ up to 30° with 0.02° increment. Nitrogen sorption isotherms were recorded on a Micrometrics TriStar II Plus gas sorption instrument. Before measurement, powder samples were degassed in a vacuum at 120 °C over 6 h. The Pore volume was calculated using the cylindrical quench solid density functional theory (QSDFT) model from the nitrogen gas sorption curve. Field emission scanning electron microscopy (FE SEM) was measured on the scanning electron microscopy (Hitachi S-4800, Japan) with a cold field emission gun working at 10.0 kV acceleration voltage. Thermogravimetric analysis (TGA) measurement was performed under nitrogen (50 mL/min) with a temperature increase from 50 °C to 800 °C at a rate of 10 °C/min. High-resolution transmission electron microscope (HR-TEM) analysis was recorded on ThermoFisher Titan G2 electron microscope, operated at 200 kV. A Fischione 2020 single tilt tomography holder was used for this work. $^{13}C$ cross-polarization magic-angle spinning nuclear magnetic resonance (CP-MAS-NMR) spectra were characted with a Bruker Avance III console operating at 700.25 MHz 1H Larmor frequency, using a double-resonance MAS probe supporting zirconia MAS rotors with 2.5 mm outer diameter spinning at 25 kHz MAS frequency. UV-vis absorption spectra were performed on a Perkin-Elmer Lambda 900 spectrometer. The Ocean optics spectrograph (CDS-600, Labsphere, America) was employed to detect the luminescent spectra under different stimuli. The fluorescence intensity vs. time curve was carried out on a FLS920-Combined Time Resolved & Steady State Fluorescence Spectrometer (Edinburgh Instruments) employing the Xe lamp (Xe 900) as the excitation source and RP928 photomultiplier tube as the detector at room temperature. The time-dependent photoluminescence (PL) decay dynamics of samples were stimulated by picosecond diode laser pulses (EPL-405). The pulse width, wavelength, and repetition rate were 58.8 ps, 405 nm, and 40.0 kHz, respectively.

Sensor experiments were carried out by gradually adding THF solutions of metal ions, PA and PGA ($1.5 \times 10^{-2}$ M for metal ions, $6 \times 10^{-4}$ M for PA, and $1 \times 10^{-3}$ M for PGA) into ETTA-CHDA-COF suspensions in THF (50 $\mu$g $mL^{-1}$, 3.0 mL) at intervals of 5 mins. Finally, fluorescence spectra with different intensity were recorded at room temperature excited by the same wavelength of 360 nm. Besides, a certain amount of extra THF solvent was added to the quartz cell to make the total solution volume constant (3 mL) so that the concentration of COF stays the same in different measurements. All the changes of luminescence spectra in the detection of $Fe^{3+}$, PA and PGA occurred immediately after the addition of each analyte. Each fluorescence spectra of different detection limit was measured at least for three times to get the reliable value.

## Photoluminescence Quantum Yield (PLQY) Measurement

PLQY was recorded with a HORIBA Jobin-Yvon Fluorolog 3-22 Tau-3 with an intergrating sphere (F-3810 from Horiba Jobin Yvon) in the air atmosphere using the method described in the literature.[11] Notably, one sample's spectra were recorded with the same excitation and emission monochromator bandpass (slits opening). Besides, most of the excitation profiles were recorded through a neutral gauze density (ND) filter(s) to avoid exceeding the intensity of the excitation profiles, and the ND value of the filter used was then taken into account in the calculation of the absorbed value, giving this last equation: The PLQY is defined as

$$\Phi_{\mathrm{pL}} = \frac{emittted\ photons}{absorbed\ photons}\ \mathrm{ND_{selected}}$$

## Computational Details

Molecular modeling and Pawley refinement were carried out using Reflex, a software package for crystal determination from XRD pattern as implemented in Materials Studio (MS) modeling version 4.4 (Accelrys Inc.).[12-13] Pawley refinement was utilized to optimize the lattice parameters iteratively until the $R_{\mathrm{P}}$ and $R_{\mathrm{WP}}$ values converge. Geometries of a monolayer, AA and AB stacking for TFB-CHDA COF,

TFPB-CHDA COF, TFPPy-CHDA COF, TFPPery-CHDA COF, TFPPery-PDA COF, and ETTA-CHDA COF were calculated using density functional tight binding (DFTB) implemented in DFTB$^+$ version 20.1.[14] All-atom pairs were calculated via standard parameters from the mio-0-1 parameter set.[15]

## High-Resolution Transmission Electron Microscopy (HR-TEM)

For the sample preparation, ETTA-CHDA COF, TFPPery-CHDA COF, and TFB-CHDA COF was bath-sonicated in dichloromethane or THF (0.01 mg/mL), respectively, for 5 min. Then, 10 μL of the suspension was placed onto a microgrids and the excessive amount of solvent was blotted by a filter paper. This procedure was repeated for 3 times. The microgrid was dried under reduced pressure for 3 hours before observation by the electron microscopy. TEM samples were prepared by using a TEM grid precoated with a lacy microgrid (NS-C15, pore size 1.5–8 μm) was purchased from Okenshoji Co., Ltd. For low electron dose imaging, we used a CVD-grown monolayer graphene-coated quantifoil gold grids, with a hole size of 2 μm and space between the hole of 4 μm, were purchased from Graphenea.

HR-TEM imaging of ETTA-CHDA COF, TFPPery-CHDA COF, and TFB-CHDA COF was carried out on Titan G2 electron microscope (Thermo Fischer Scientific, Inc., operated at 200 kV) equipped with a Schottky XFEG electron source, S-TWIN objective lens, image Cs-corrector (CEOS GmbH), with Gatan 2k×2k UltrascanXP1000 camera (Gatan, Inc.). A Fischione 2020 single tilt tomography holder was used for this work. The raw TEM images were collected as a .dm4 format file on DigitalMicrograph software (Gatan, Inc.) and further processed using ImageJ 1.53c software. The HR-TEM images were filtered using a bandpass filter (filtering structures smaller than 3 pixels and larger than 40 pixels, tolerance of direction: 5%). TEM simulation images were generated by using a multi-slice procedure implemented in the ELBis software.[16]

Low electron dose imaging of ETTA-CHDA COF was carried out on Themis Z

(Thermo Fischer Scientific Inc., operated at 300 kV) equipped with Gatan 4k×4k K2 direct electron detection camera (Gatan, Inc.) operated at a counting mode. A FEI double tilt holder was used for this work. Image collection was conducted with an electron dose rate of 2 $e^{-}$ $Å^{-2}$ $s^{-1}$ to suppress beam-induced deformation of the crystalline samples. Fifty successive images collected at an exposure time of 0.5 s were aligned and superimposed by using a drift correction function implemented in the DigitalMicrograph software. To extract a periodic feature of the crystals, the images were processed by the Local 2D Wiener filter of HREM Filters Pro (HREM Research Inc.) implemented as DigitalMicrograph plugin.

## **Section B:** Synthetic Details

**Scheme S1.** Synthetic route towards TFB-CHDA COF.

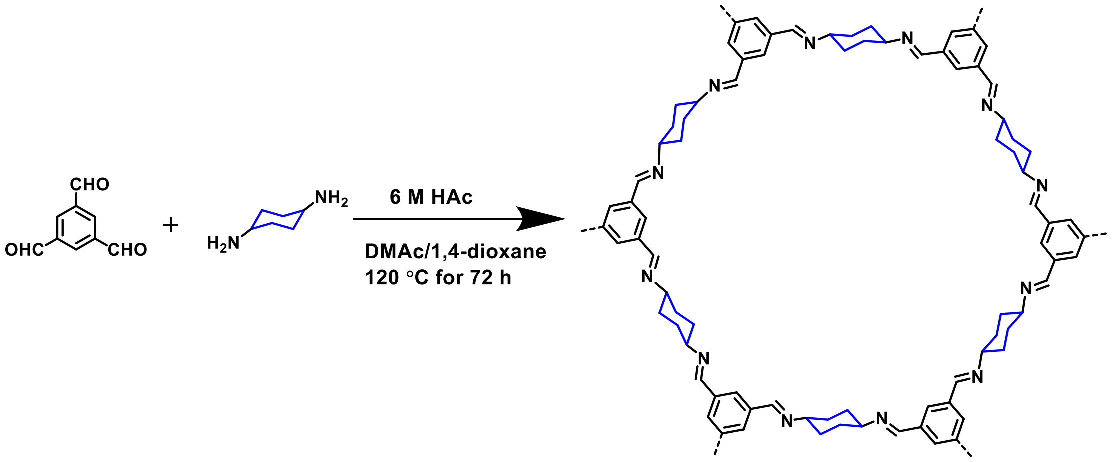


A microwave tube (15 mL) placed with TFB (19.0 mg, 0.117 mmol), CHDA (20.0 mg, 0.175mmol), DMAc (2.4 mL), 1,4-dioxane (0.6 ml), and 6 M HAc (50 $\mu$L) was sonicated for 30 s and degassed through three freeze-pump-thaw cycles before sealing under vacuum. The tube was then sealed at 120 °C for 3 days. After cooling to room temperature, the white-colored precipitate was filtered and washed with degassed THF and acetone several times. The powder was dried under vacuum at 60 °C for 12 h to produce TFB-CHDA COF in 87% yield. Elemental analysis: calculated C (79.61%), H (8.02%), N (12.38%) and observed C (71.74%), H (7.29%), N (13.06%).

**Scheme S2.** Synthetic route towards TFPB-CHDA COF.

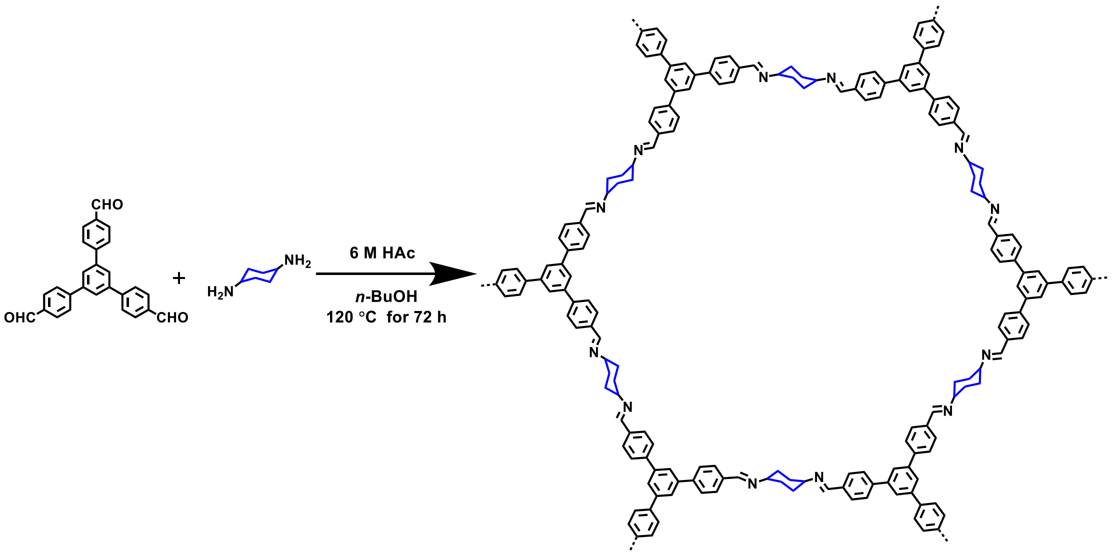


A microwave tube (15 mL) placed with TFPB (20.0 mg, 0.051 mmol), CHDA (17.5mg, 0.0765 mmol), *n*-BuOH (3 mL), and 6 M HAc (50 $\mu$L) was sonicated for 30 s and degassed through three freeze-pump-thaw cycles before sealing under vacuum. The tube was then sealed at 120 °C for 3 days. After cooling to room temperature, the white-colored precipitate was filtered and washed with degassed THF and acetone several times. The powder was dried under vacuum at 60 °C for 12 h to produce TFPB-CHDA COF in 91% yield. Elemental analysis: calculated C (87.19%), H (6.65%), N (6.16%) and observed C (84.17%), H (2.79%), N (7.09%).

**Scheme S3.** Synthetic route towards ETTA-CHDA COF.

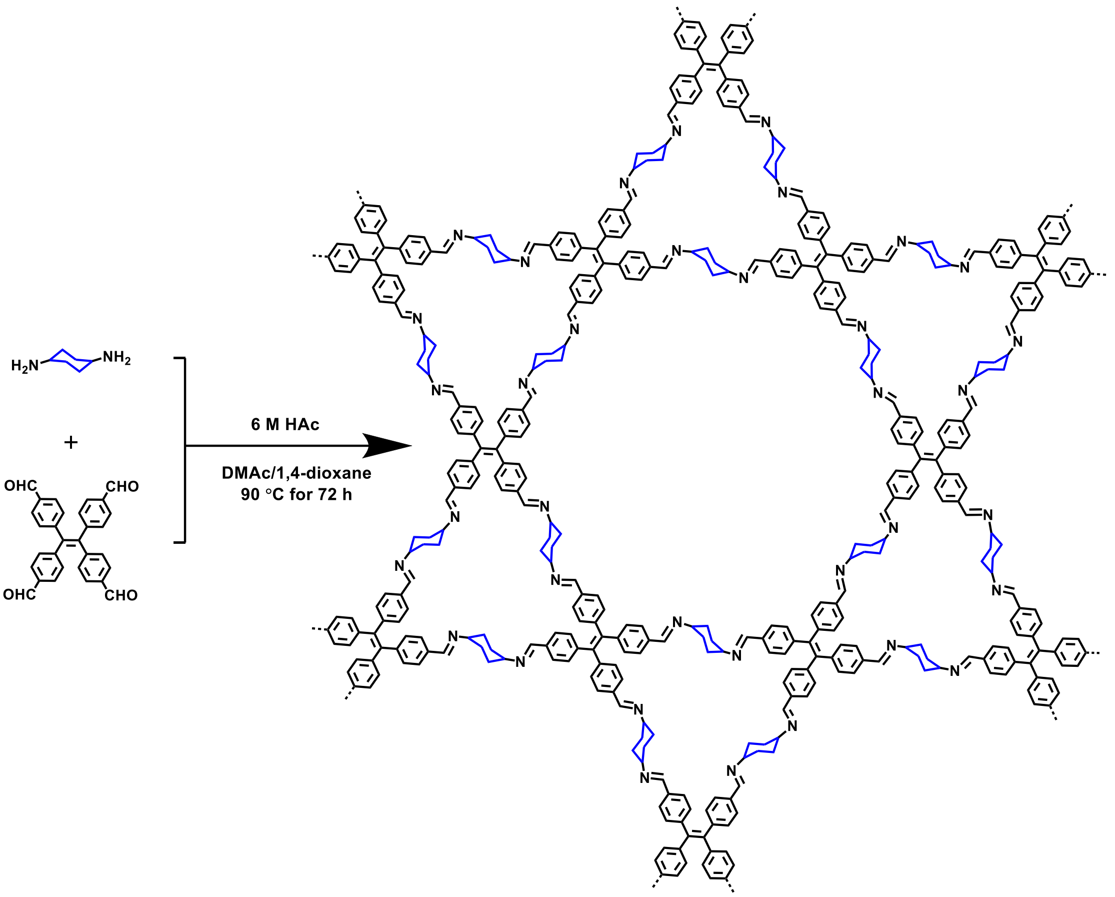


A microwave tube (15 mL) placed with ETTA (19.0 mg, 0.043 mmol), CHDA (10.0 mg, 0.086 mmol), DMAc (0.8 mL), and 1,4-dioxane (0.2 mL) was sonicated for 30 s and degassed through three freeze-pump-thaw cycles before sealing under vacuum. Afterward, 6 M HAc (20 $\mu$L) was added to the tube and then sealed at 90 °C for over 3 days. After cooling to room temperature, the light yellow-colored precipitate was filtered and washed with degassed THF and acetone several times. The powder was dried with nitrogen gas flow to produce ETTA-CHDA COF in 82% yield. Elemental analysis: calculated C (84.53%), H (6.08%), N (9.39%) and observed C (79.52%), H (6.66%), N (8.52%).

**Scheme S4.** Synthetic route towards TFPPy-CHDA COF.

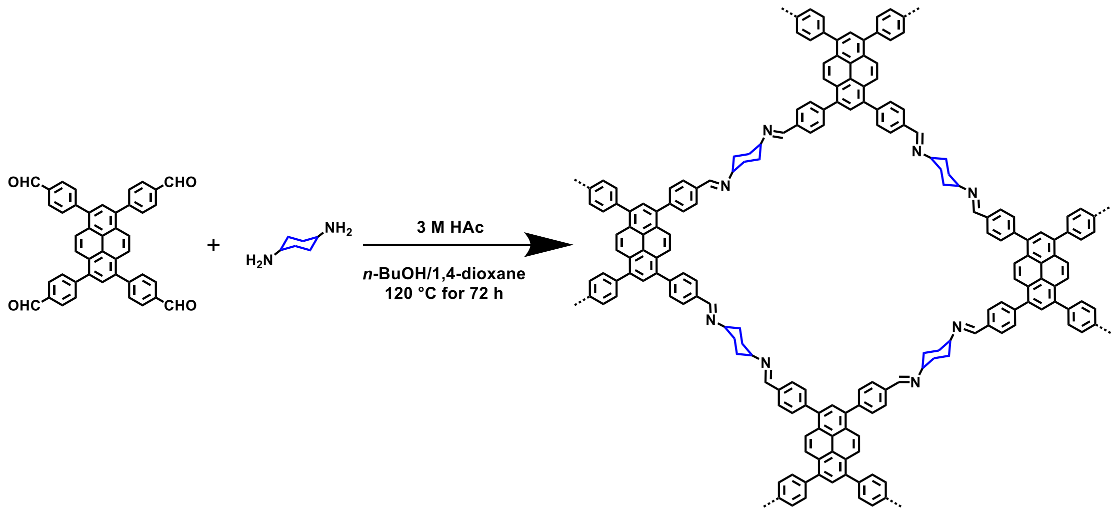


A microwave tube (15 mL) placed with TFPPy (27.1 mg, 0.043 mmol), CHDA (10.0 mg, 0.086 mmol), *n*-BuOH (1.2 mL), and 1,4-dioxane (0.3 mL) was sonicated for 30 s and degassed through three freeze-pump-thaw cycles before sealing under vacuum. Afterward, 3 M HAc (50 $\mu$L) was added to the tube and then sealed at 120 °C for over 3 days. After cooling to room temperature, the yellow-colored precipitate was filtered and washed with degassed THF and acetone several times. The powder was dried under vacuum at 60 °C for 12 h to produce TFPPy-CHDA COF in 93% yield. Elemental analysis: calculated C (87.23%), H (5.49%), N (7.23%) and observed C (82.87%), H (6.23%), N (10.81%).

**Scheme S5.** Synthetic route towards TFPPery-CHDA COF.

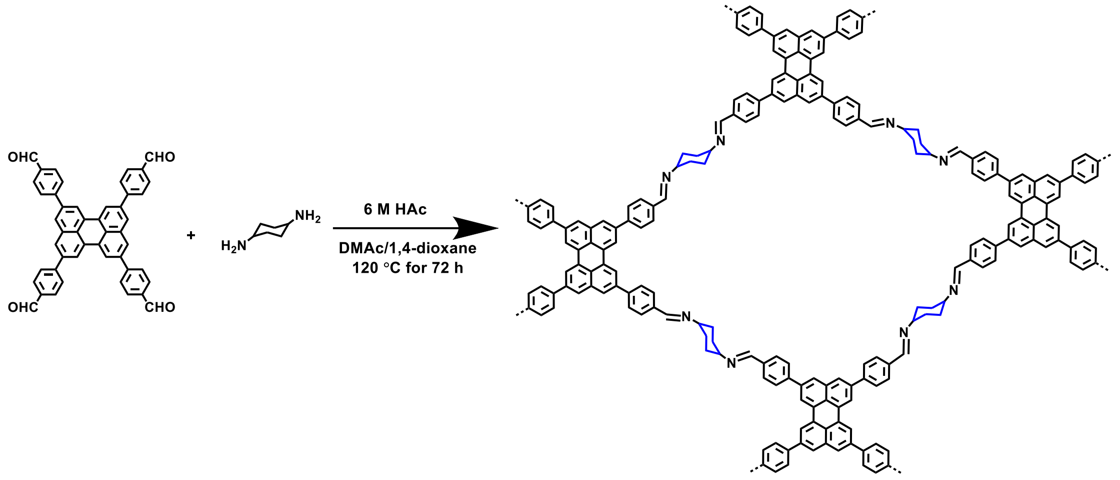


A microwave tube (15 mL) placed with TFPPery (29.3 mg, 0.043 mmol), CHDA (9.4 mg, 0.086 mmol), DMAc (1.2 mL), 1,4-dioxane (0.3 mL), and 6 M HAc (100 μL) was sonicated for 30 s and degassed through three freeze-pump-thaw cycles before sealing under vacuum. Afterward, the tube and the solution were sealed at 120 °C for over 3 days. After cooling to room temperature, the orange-colored precipitate was filtered and washed with degassed THF and acetone several times. The powder was dried under vacuum at 60 °C for 12 h to produce TFPPery-CHDA COF in 91% yield. Elemental analysis: calculated C (87.77%), H (5.40%), N (6.82%) and observed C (82.11%), H (5.92%), N (5.08%).

**Scheme S6.** Synthetic route towards TFPPery-PDA COF.

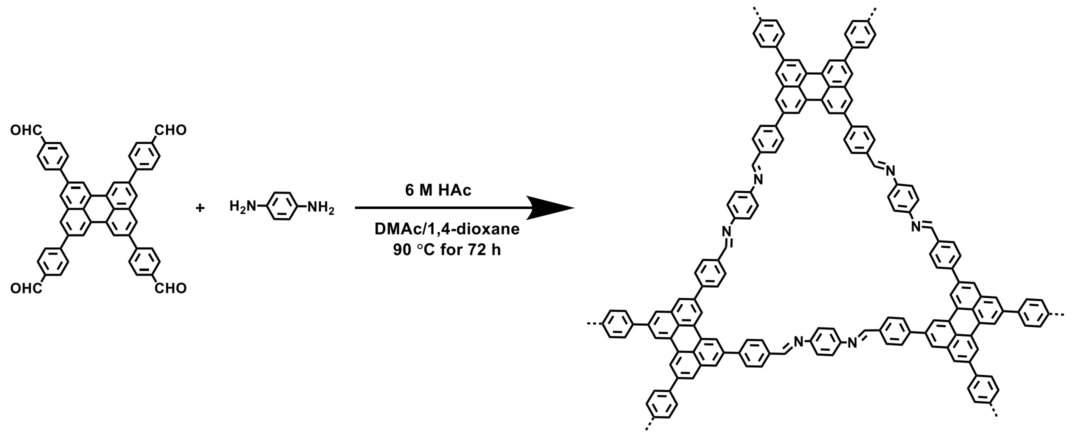


A microwave tube (15 mL) placed with TFPPery (29.3 mg, 0.043 mmol), PDA (10.0 mg, 0.086 mmol), DMAc (1.25 mL), 1,4-dioxane (1.25 mL), and 6 M HAc (100 $\mu$L) was sonicated for 30 s and degassed through three freeze-pump-thaw cycles before sealing under vacuum. After that, the solution was then sealed at 90 °C for over 3 days. After cooling to room temperature, the dark red-colored precipitate was filtered and washed with degassed THF and acetone several times. The powder was dried under vacuum at 60 °C for 12 h to produce TFPPy-PDA COF in 93% yield. Elemental analysis: calculated C (88.64%), H (4.46%), N (6.89%) and observed C (82.91%), H (5.22%), N (5.60%).

## **Section C:** Characteristics

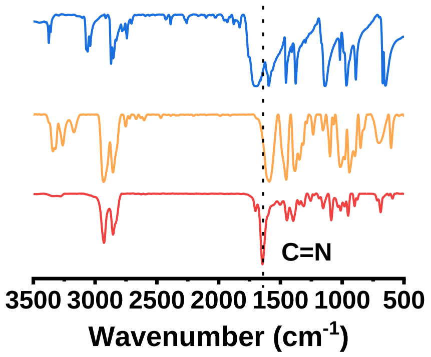


**Figure S1.** FT-IR spectra of TFB (blue), CHDA (yellow), and TFB-CHDA COF (red), respectively.

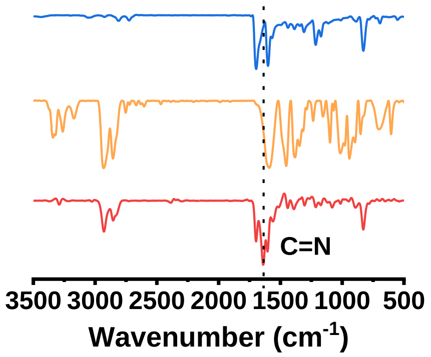


**Figure S2.** FT-IR spectra of TFPB (blue), CHDA (yellow), and TFPB-CHDA COF (red), respectively.

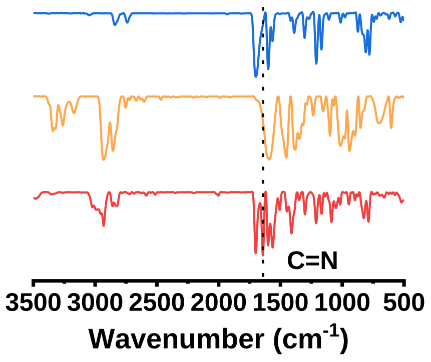


**Figure S3.** FT-IR spectra of ETTA (blue), CHDA (yellow), and ETTA-CHDA COF (red), respectively.

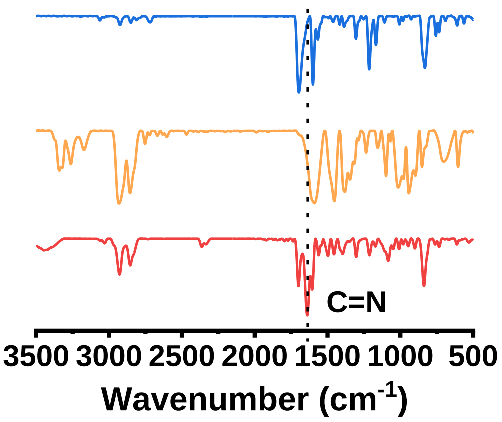


**Figure S4.** FT-IR spectra of TFPPy (blue), CHDA (yellow), and TFPPy-CHDA COF (red), respectively.

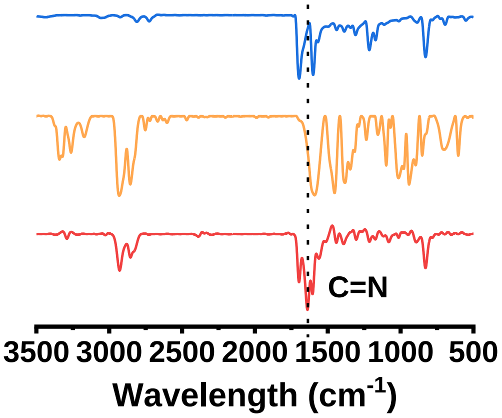


**Figure S5.** FTIR spectra of TFPPery (blue), CHDA (yellow), and TFPPery-CHDA COF (red), respectively.

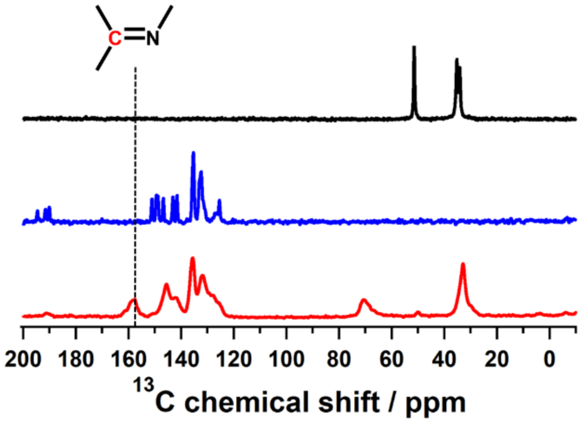


**Figure S6.** $^{13}$C CP-MAS-NMR spectra of CHDA (black), ETTA (blue) and ETTA-CHDA COF (red).

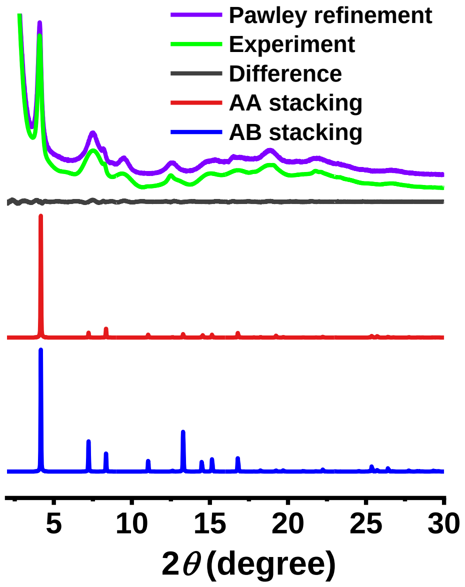


**Figure S7.** PXRD of TFB-CHDA COF analyzed by different simulated patterns: slipped AA stacking mode (red) and AB eclipsed stacking mode (blue) with the experimental pattern (green) combined Pawley refined pattern (purple) and the difference (gray) between experimental and stimulated patterns.

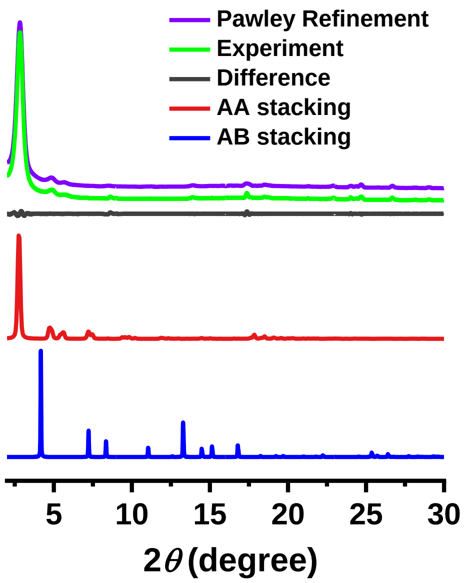


**Figure S8.** PXRD of TFPB-CHDA COF analyzed by different simulated patterns: slipped AA stacking mode (red) and AB eclipsed stacking mode (blue) with the experimental pattern (green) combined Pawley refined pattern (purple) and the difference (gray) between experimental and stimulated patterns.

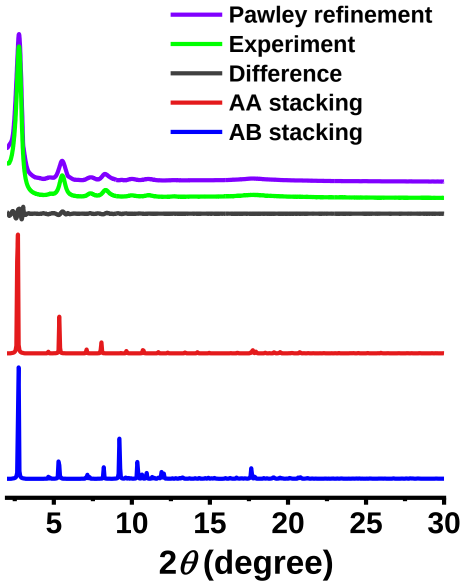


**Figure S9.** PXRD of ETTA-CHDA COF analyzed by different simulated patterns: slipped AA stacking mode (red) and AB eclipsed stacking mode (blue) with the experimental pattern (green) combined Pawley refined pattern (purple) and the difference (gray) between experimental and stimulated patterns.

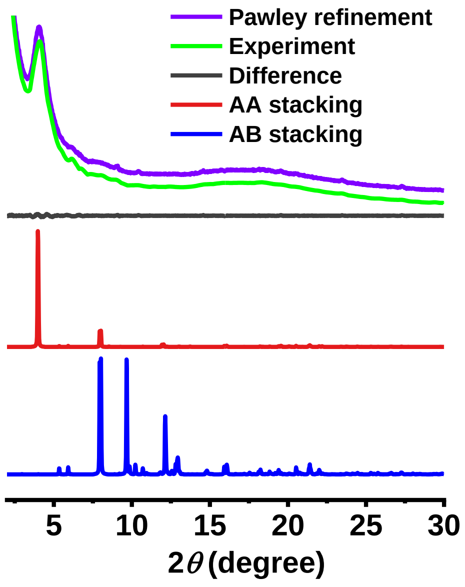


**Figure S10.** PXRD of TFPPy-CHDA COF analyzed by different simulated patterns: slipped AA stacking mode (red) and AB eclipsed stacking mode (blue) with the experimental pattern (green) combined Pawley refined pattern (purple) and the difference (gray) between experimental and stimulated patterns.

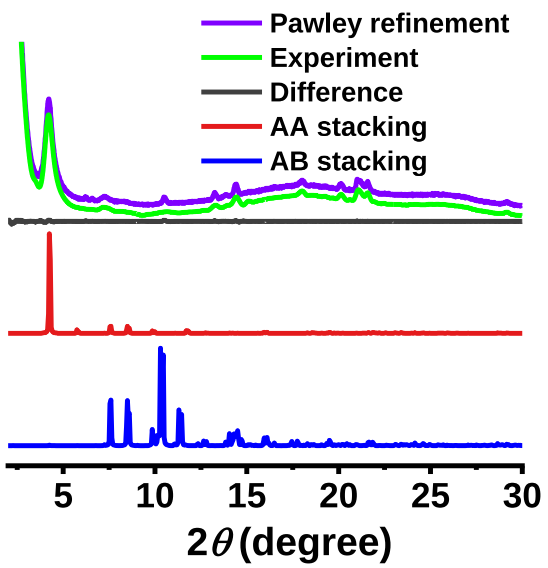


**Figure S11.** PXRD of TFPPery-CHDA COF analyzed by different simulated patterns: slipped AA stacking mode (red) and AB eclipsed stacking mode (blue) with the experimental pattern (green) combined Pawley refined pattern (purple) and the difference (gray) between experimental and stimulated patterns.

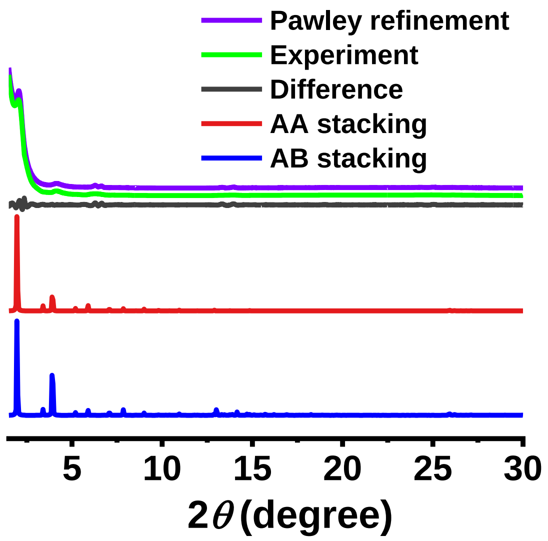


**Figure S12.** PXRD of TFPPery-PDA COF analyzed by different simulated patterns: slipped AA stacking mode (red) and AB eclipsed stacking mode (blue) with the experimental pattern (green) combined Pawley refined pattern (purple) and the difference (gray) between experimental and stimulated patterns.

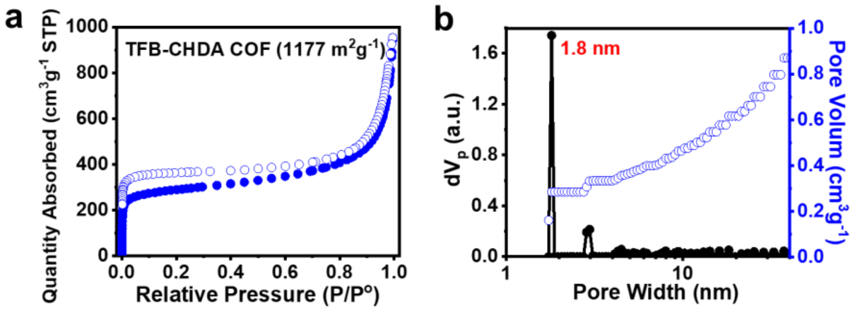


**Figure S13.** (a) Nitrogen adsorption-desorption isotherms curves of TFB-CHDA COF. (b) The pore size distribution and corresponding cumulative pore volume were stimulated by fitting the nitrogen adsorption-desorption isotherms curves at 77 K with the QSDFT model.

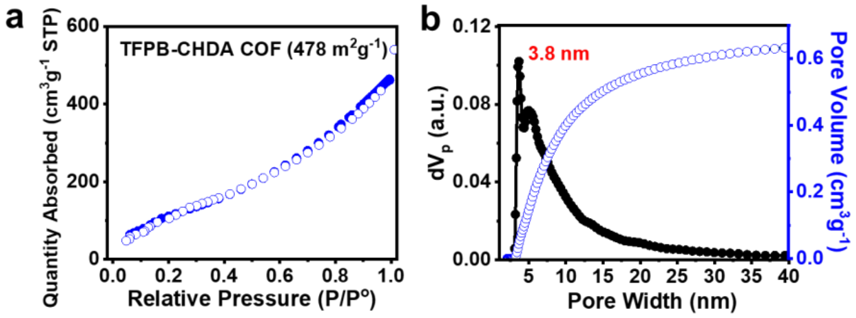


**Figure S14.** (a) Nitrogen adsorption-desorption isotherms curves of TFPB-CHDA COF. (b) The pore size distribution and corresponding cumulative pore volume were stimulated by fitting the nitrogen adsorption-desorption isotherms curves at 77 K with the QSDFT model.

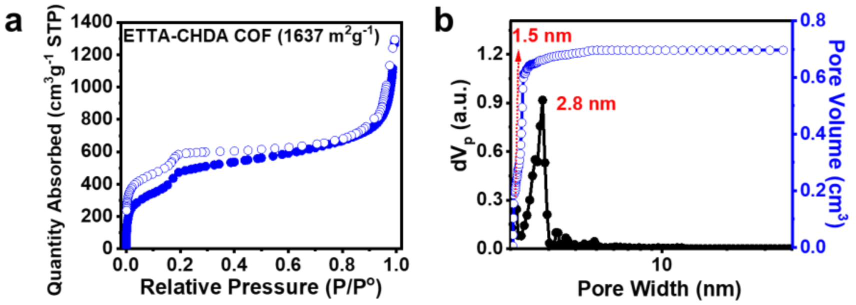


**Figure S15.** (a) Nitrogen adsorption-desorption isotherms curves of ETTA-CHDA COF. (b) The pore size distribution and corresponding cumulative pore volume were stimulated by fitting the nitrogen adsorption-desorption isotherms curves at 77 K with the QSDFT model.

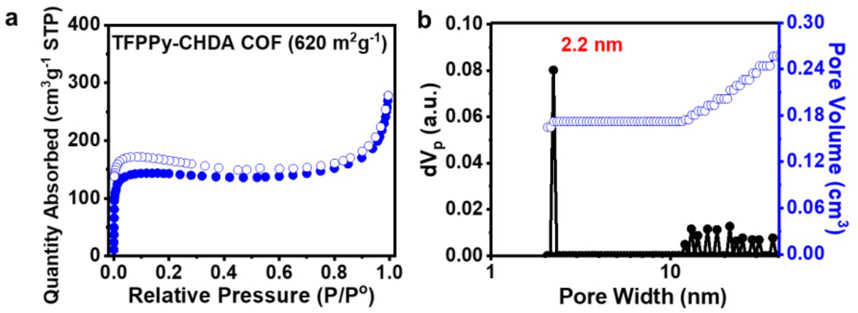


**Figure S16.** (a) Nitrogen adsorption-desorption isotherms curves of TFPPy-CHDA COF. (b) The pore size distribution and corresponding cumulative pore volume were stimulated by fitting the nitrogen adsorption-desorption isotherms curves at 77 K with the QSDFT model.

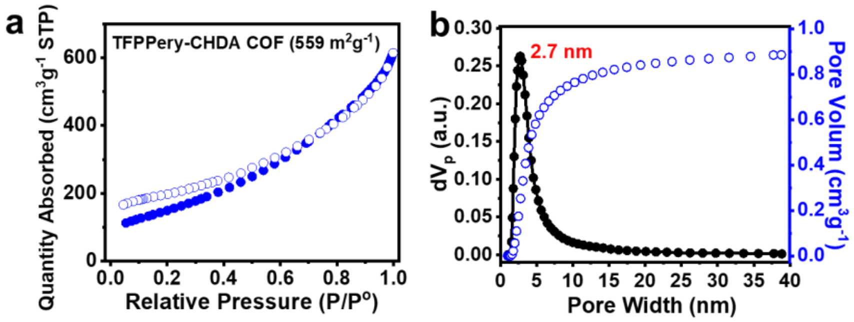


**Figure S17.** (a) Nitrogen adsorption-desorption isotherms curves of TFPPery-CHDA COF. (b) The pore size distribution and corresponding cumulative pore volume were stimulated by fitting the nitrogen adsorption-desorption isotherms curves at 77 K with the QSDFT model.

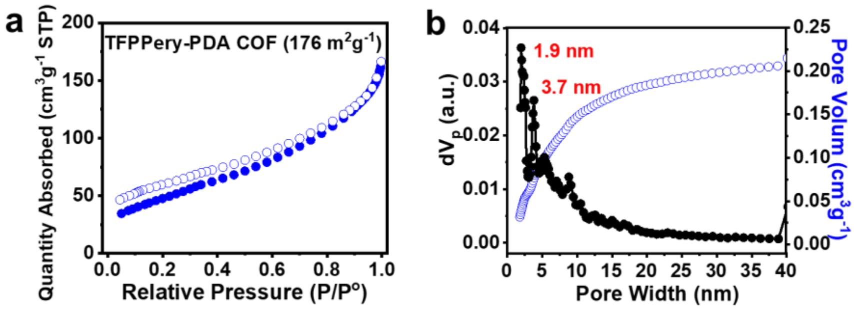


**Figure S18.** (a) Nitrogen adsorption-desorption isotherms curves of TFPPery-PDA COF. (b) The pore size distribution and corresponding cumulative pore volume were stimulated by fitting the nitrogen adsorption-desorption isotherms curves at 77 K with the QSDFT model.

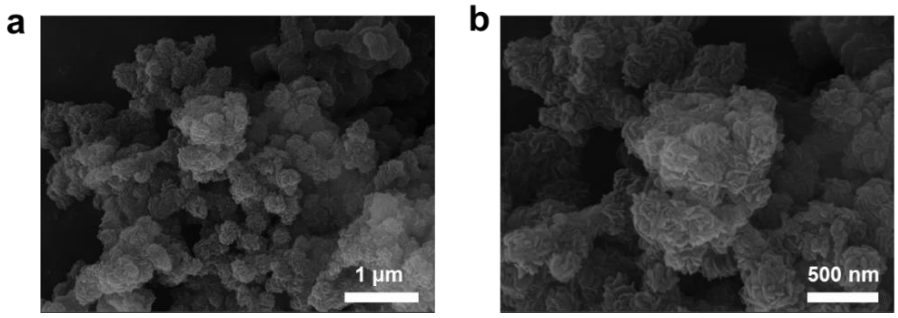


**Figure S19.** SEM images of TFB-CHDA COF.

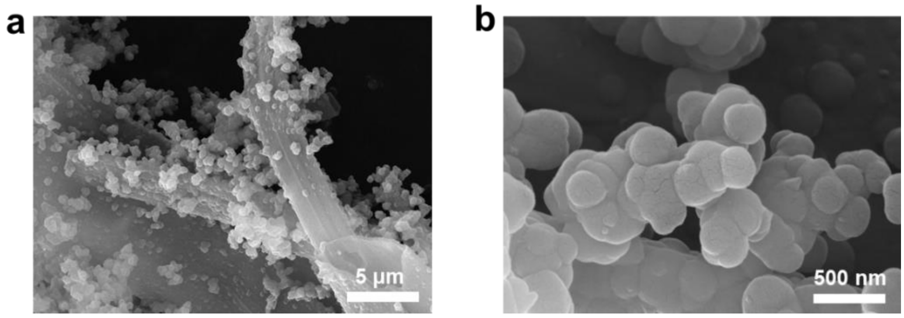


**Figure S20.** SEM images of TFPB-CHDA COF.

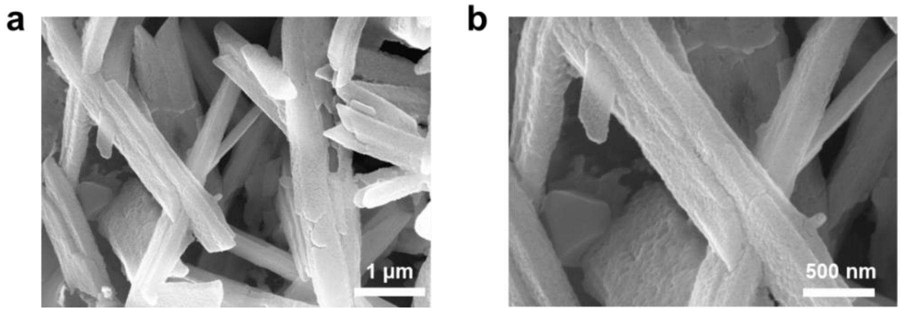


**Figure S21.** SEM images of ETTA-CHDA COF.

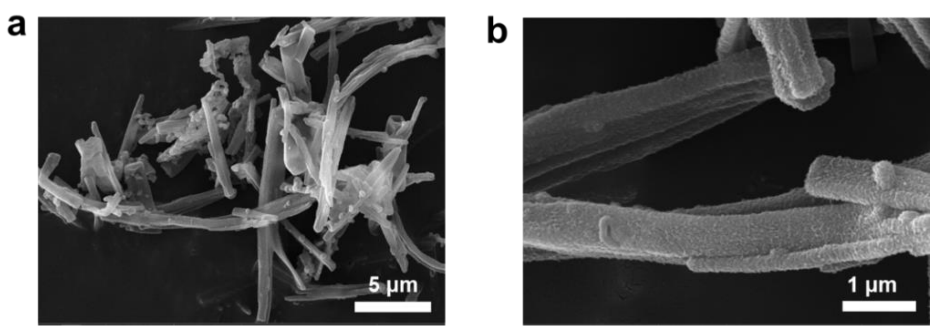


**Figure S22.** SEM images of TFPPy-CHDA COF.

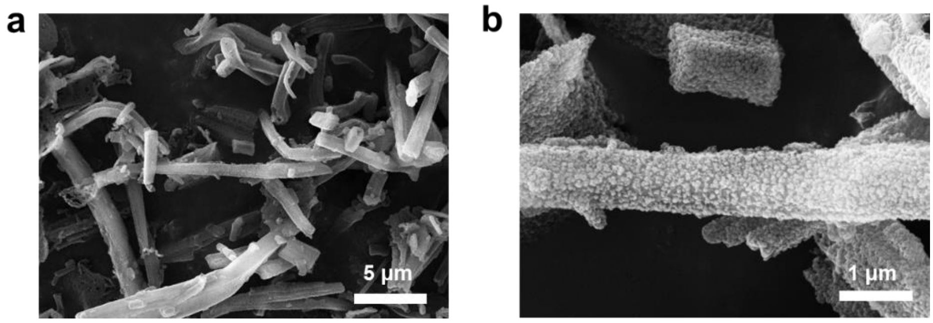


**Figure S23.** SEM images of TFPPery-CHDA COF.

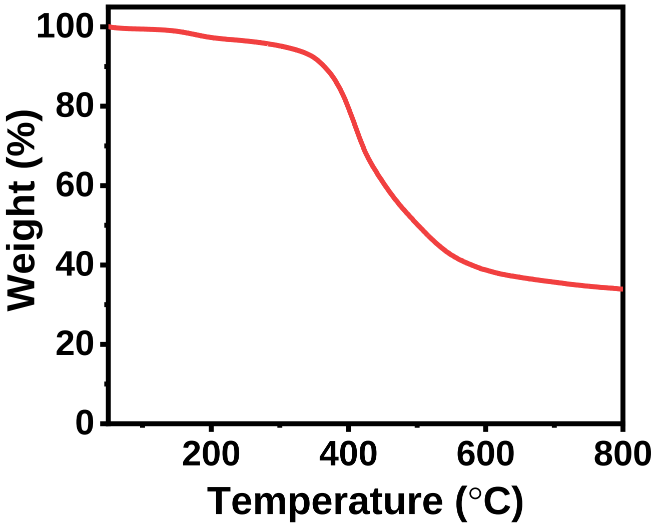


**Figure S24.** TGA curve of TFB-CHDA COF under nitrogen atmosphere.

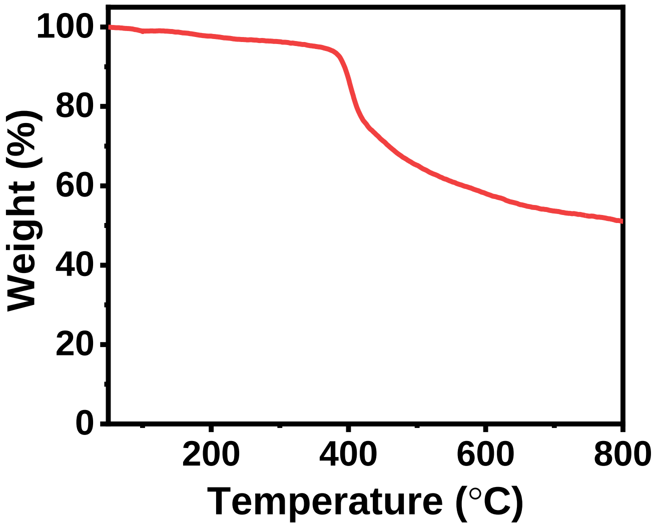


**Figure S25.** TGA curve of TFPB-CHDA COF under nitrogen atmosphere.

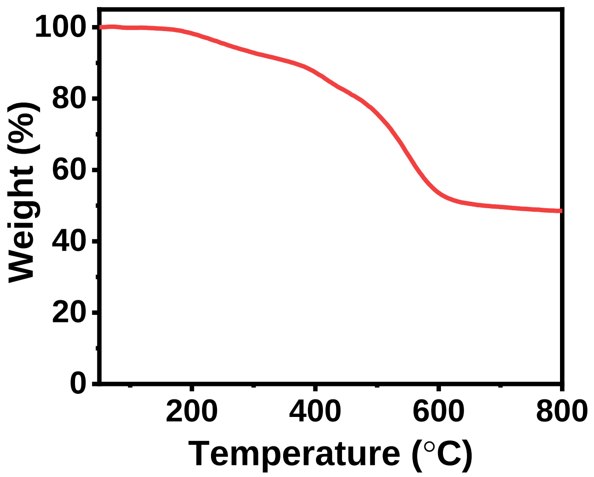


**Figure S26.** TGA curve of ETTA-CHDA COF under nitrogen atmosphere.

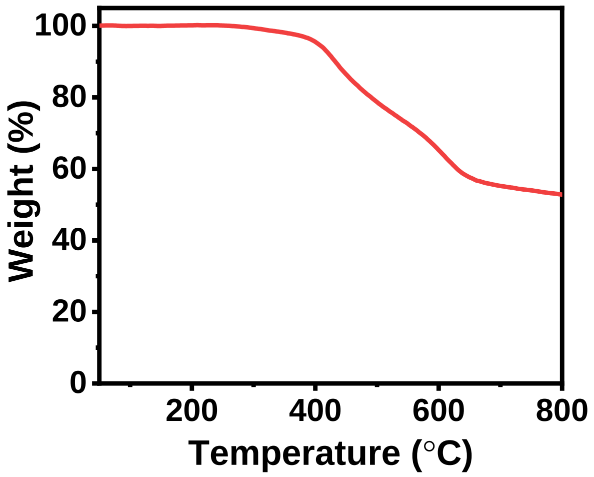


**Figure S27.** TGA curve of TFPPy-CHDA COF under nitrogen atmosphere.

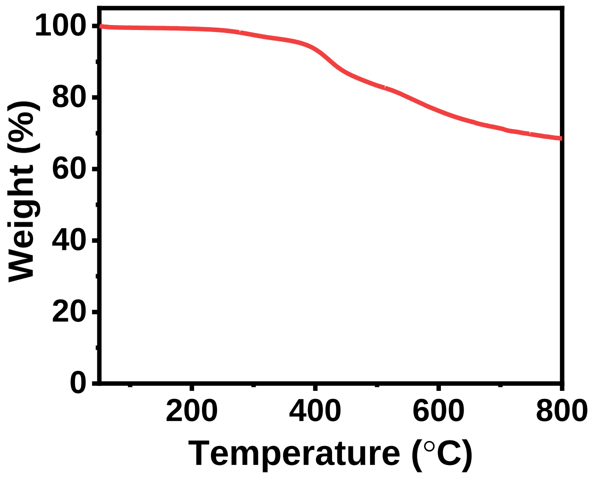


**Figure S28.** TGA curve of TFPPery-CHDA COF under nitrogen atmosphere.

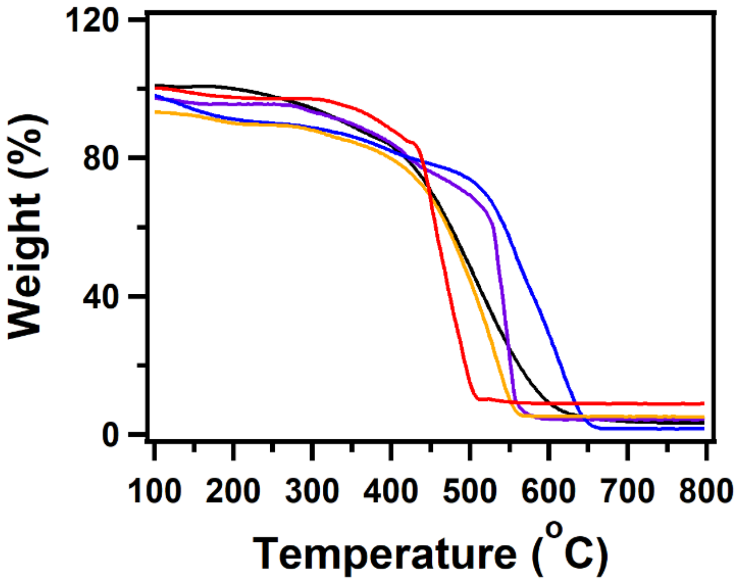


**Figure S29.** TGA curve of TFPPery-CHDA COF under nitrogen atmosphere.

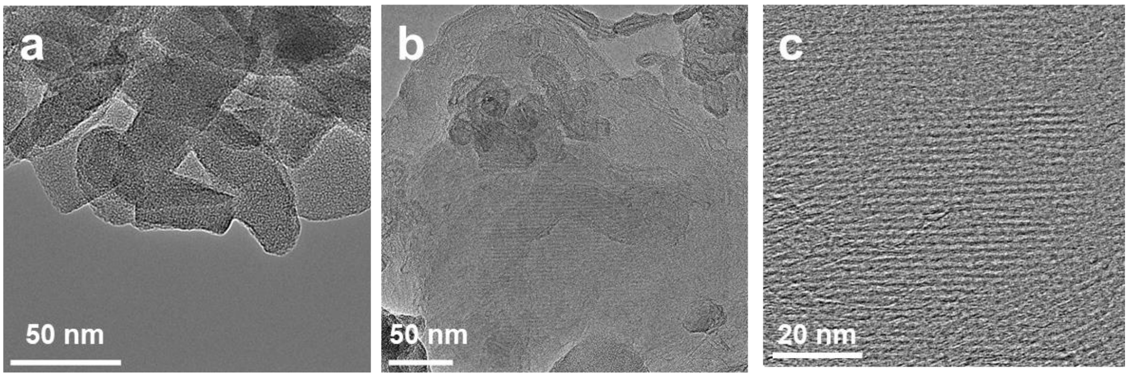


**Figure S30.** HR-TEM images of ETTA-CHDA COF with kgm topology. (a)(b) Low-magnification images of ETTA-CHDA COF, showing its morphology. (c) TEM image showing a periodic structure.

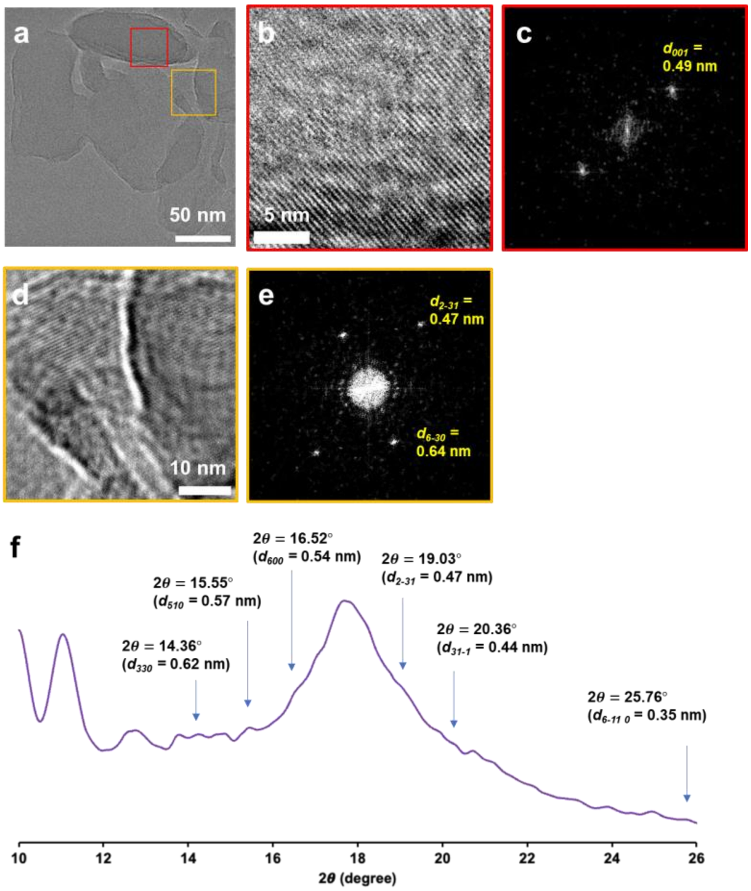


**Figure S31.** HR-TEM and FFT images of ETTA-CHDA COF. (a) TEM images of ETTA-CHDA COF. (b) Magnified TEM image of the area inside a red square in (a). (c) FFT image of (b). (d) Magnified TEM image of the area inside an orange square in (a). (e) FFT image of (d). (f) Magnified PXRD simulation spectra. Blue arrows indicate the diffraction observed in the TEM images, but not in experimental PXRD.

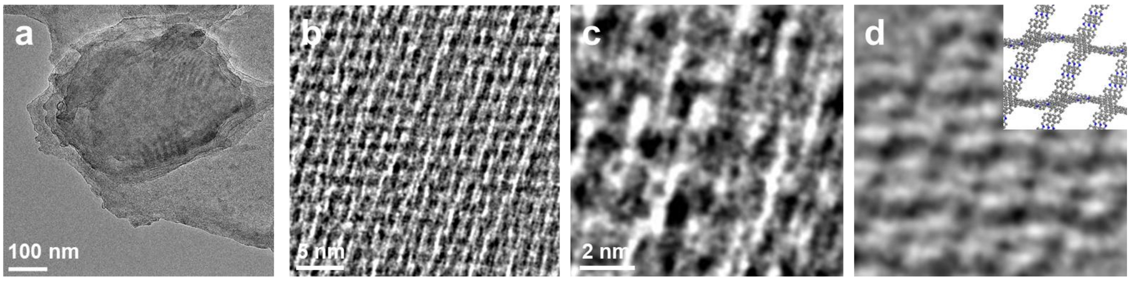


**Figure S32.** HR-TEM images of TFPPery-CHDA COF with sql topology. (a) Low-magnification images of TFPPery-CHDA COF. (b) TEM image showing a periodic structure. (c) Magnified TEM image. (d) An image simulated from molecular model. Molecular model is partially overlaid.

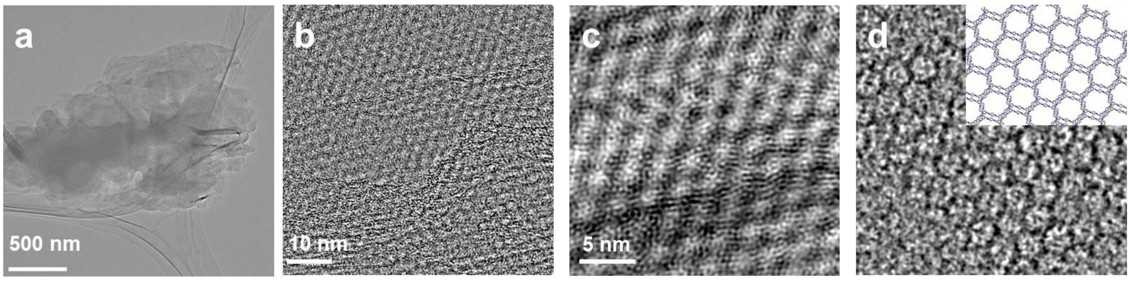


**Figure S33.** HR-TEM images of TFB-CHDA COF with hcb topology. (a) Low-magnification images of TFB-CHDA COF. (b) TEM image showing a periodic structure. (c) Magnified TEM image. (d) An image simulated from molecular model. Molecular model is partially overlaid.

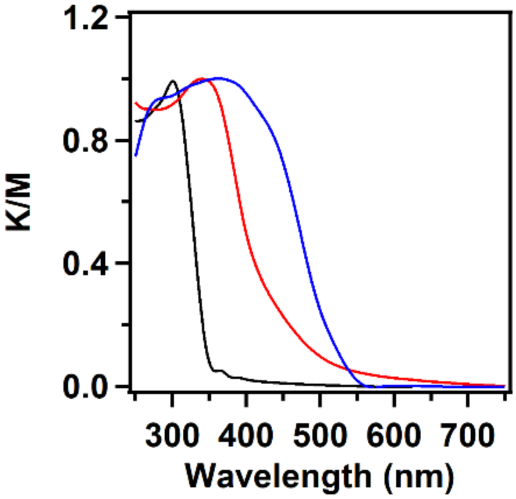


**Figure S34.** The UV–vis diffuse reflectance spectra (DRS) of TFB (black curve), TFPB (blue curve) and ETTA (red curve) monomers.

## Section D: Fluorescent Sensor Experiments

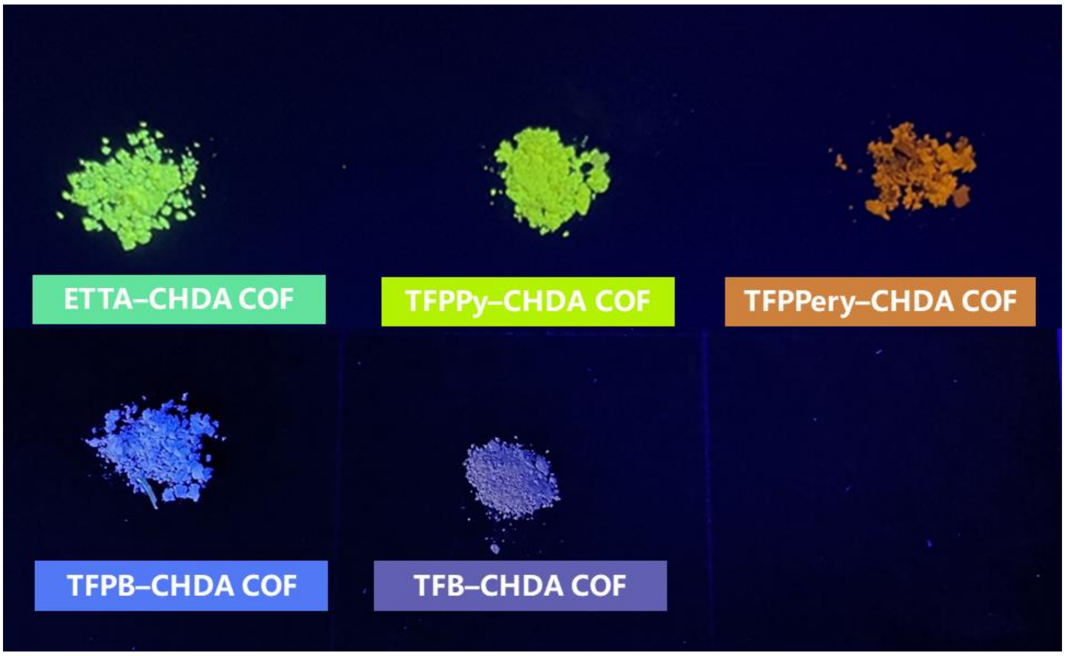


**Figure S35.** The photoluminescent images of CHDA-based COFs powders under UV-illuminant of 365 nm.

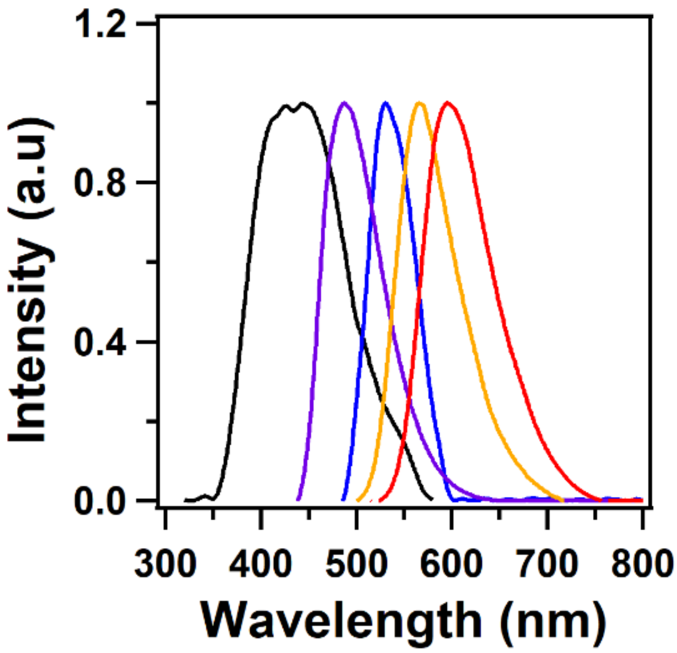


**Figure S36**. PL spectra of TFB (black curve), TFPB (purple curve), ETTTA (blue curve), TFPPy (orange curve) and TFPPery (red curve) in the solid-state.

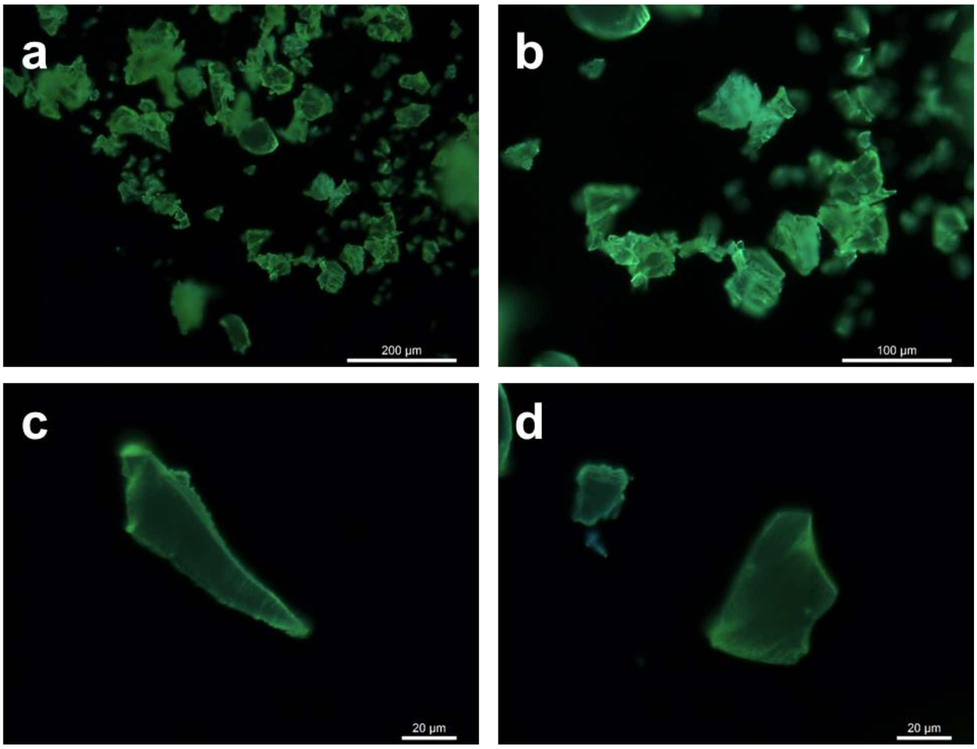


**Figure S37.** The fluorescence microscope images of the representative ETTA-CHDA COF with the highest PLQY and bright green luminescence across the particles.

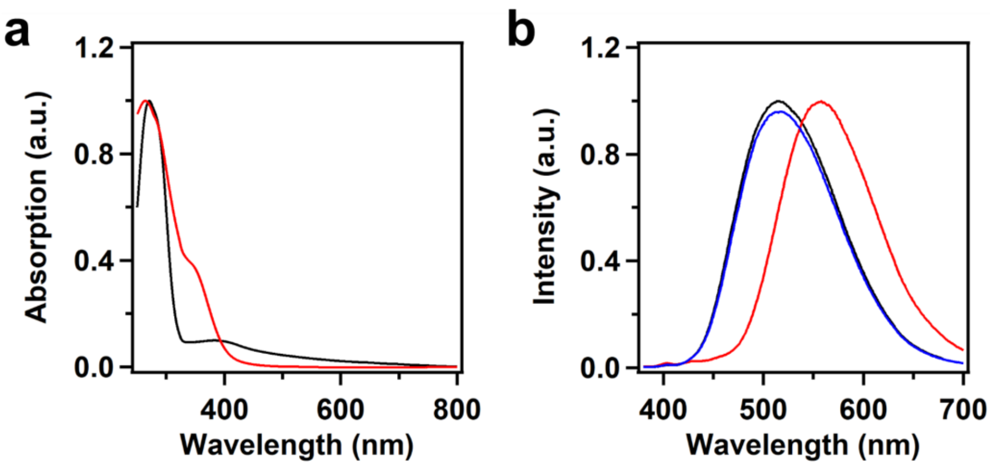


**Figure S38.** (a) Fluorescent emission and (b) UV-vis absorption spectra of pristine (black curve), acidic state (red curve) and neutralized states with TEA (blue curve) of ETTA-CHDA-COF in THF solutions. Black and blue curves were normalized with the same ratio of the initial luminescent intensity.

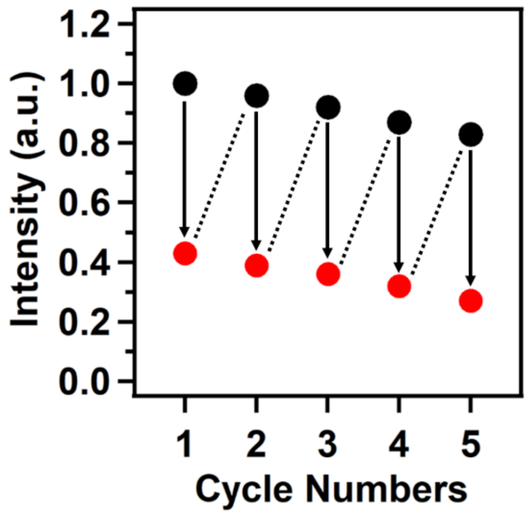


**Figure S39.** Recycling tests of ETTA-CHDA-COF after cycles of acid sensing, neutralization, and collection, based on the PL intensity in THF. From the initial intensity normalized to 1.0, addition of TFA solution (0.06 M in THF, 250 $\mu$L) resulted in the partial PL quenching (red dots). After neutralization with TEA solution (0.03 M in THF, 250 $\mu$L) and collection by filtration, recycled ETTA-CHDA-COF, re-dispersed in THF, displayed recovered PL intensity (black dot). This cycle was repeated to demonstrate the high recyclability, while the gradual decrease in the PL intensity could be due to the loss of COF powders during the filtration and re-dispersion.

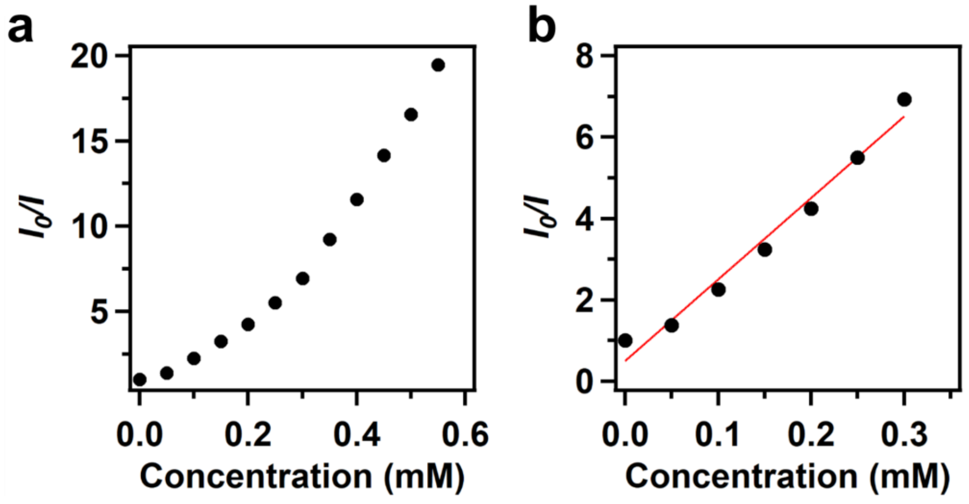


**Figure S40**. (a) Stern-Völmer plot of ETTA-CHDA COF THF solution (c = 50 $\mu$g mL$^{-1}$) upon titration with $Fe^{3+}$ solution (0.015 M in THF) at room temperature ($\lambda_{ex}$ = 360 nm). (b) Stern-Volmer plots and *K*a constant at lower concentration region for the $Fe^{3+}$

detection ($K_{sv} = 2.00 \times 10^4$ $M^{-1}$).

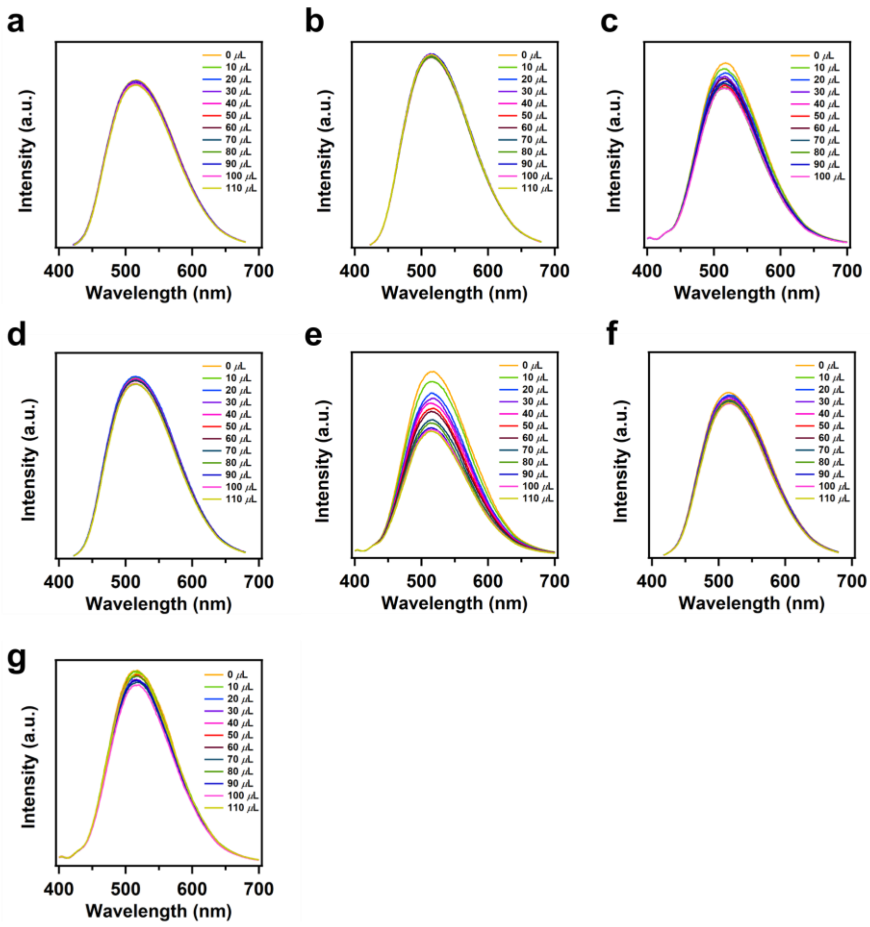


**Figure S41**. (a) Fluorescence emission spectra of ETTA-CHDA COF THF solutions (c = 50 $\mu$g $mL^{-1}$) upon successive addition of (a) $Na^{+}$, (b) $Mg^{2+}$, (c) $Cu^{2+}$, (d) $Zn^{2+}$, (e) $Ni^{2+}$, (f) $Co^{2+}$, (g) $Fe^{2+}$ solutions (0.015 M in THF) at room temperature ($\lambda_{ex}$ = 360 nm).

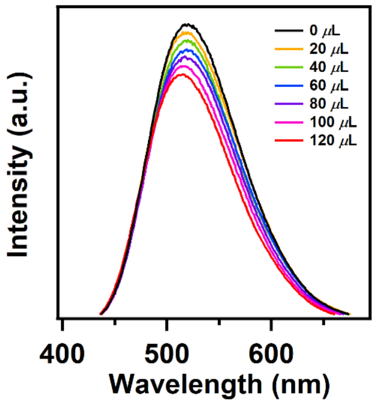


**Figure S42.** Emission spectra of ETTA-CHDA COF aqueous solution (c = 50 $\mu$g $mL^{-1}$) upon titration with $Fe^{3+}$ solution (0.03 M in water) at room temperature ($\lambda_{ex}$ =

360 nm).

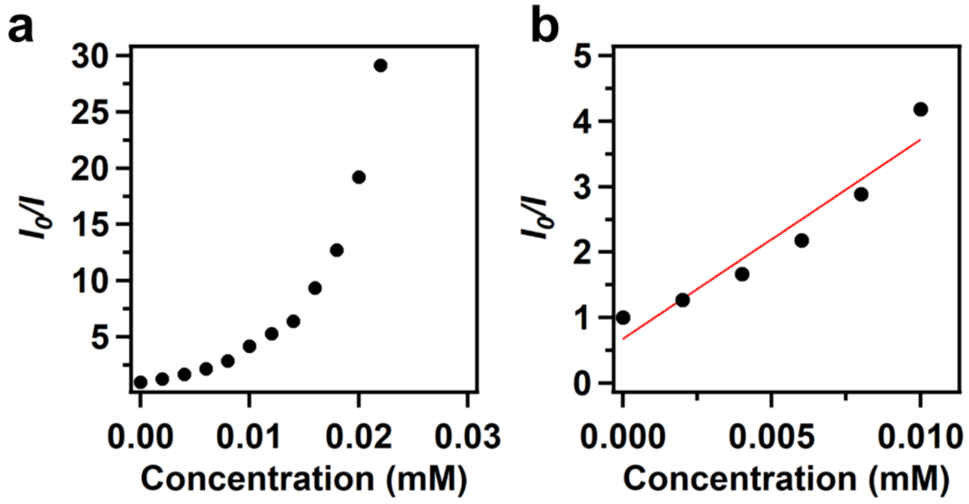


**Figure S43**. (a) Stern-Völmer plot of ETTA-CHDA COF in THF solution (c = 50 $\mu$g mL$^{-1}$) upon successive addition of PA solution ($6 \times 10^{-4}$ M in THF) at room temperature ($\lambda_{ex}$ = 360 nm). (b) Stern-Volmer plots and *K*a constant at lower concentration region for the PA detection ($K_{sv} = 3.04 \times 10^5$ M$^{-1}$).

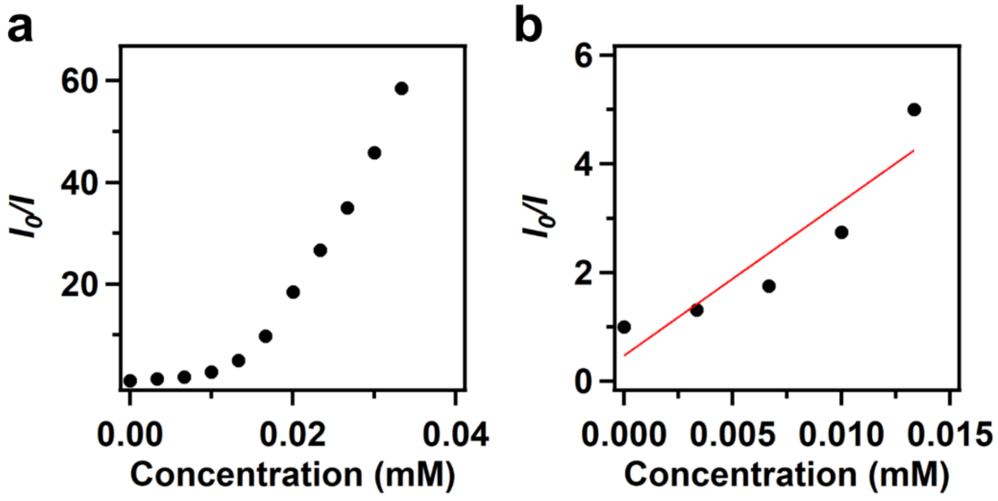


**Figure S44**. (a) Stern-Völmer plot of ETTA-CHDA COF in THF solution (c = 50 $\mu$g mL$^{-1}$) upon successive addition of PGA solution ($1 \times 10^{-3}$ M in THF) at room temperature ($\lambda_{ex}$ = 360 nm). (b) Stern-Volmer plots and *K*a constant at lower concentration region for the PGA sensing ($K_{sv} = 2.83 \times 10^5$ M$^{-1}$).

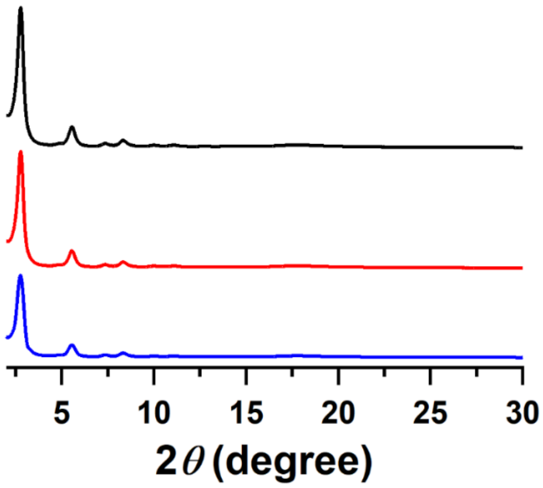


**Figure S45**. PXRD pattern of ETTA-CHDA COF after sensing for $Fe^{3+}$ (blue curve), PA (red curve) and PGA (black curve).

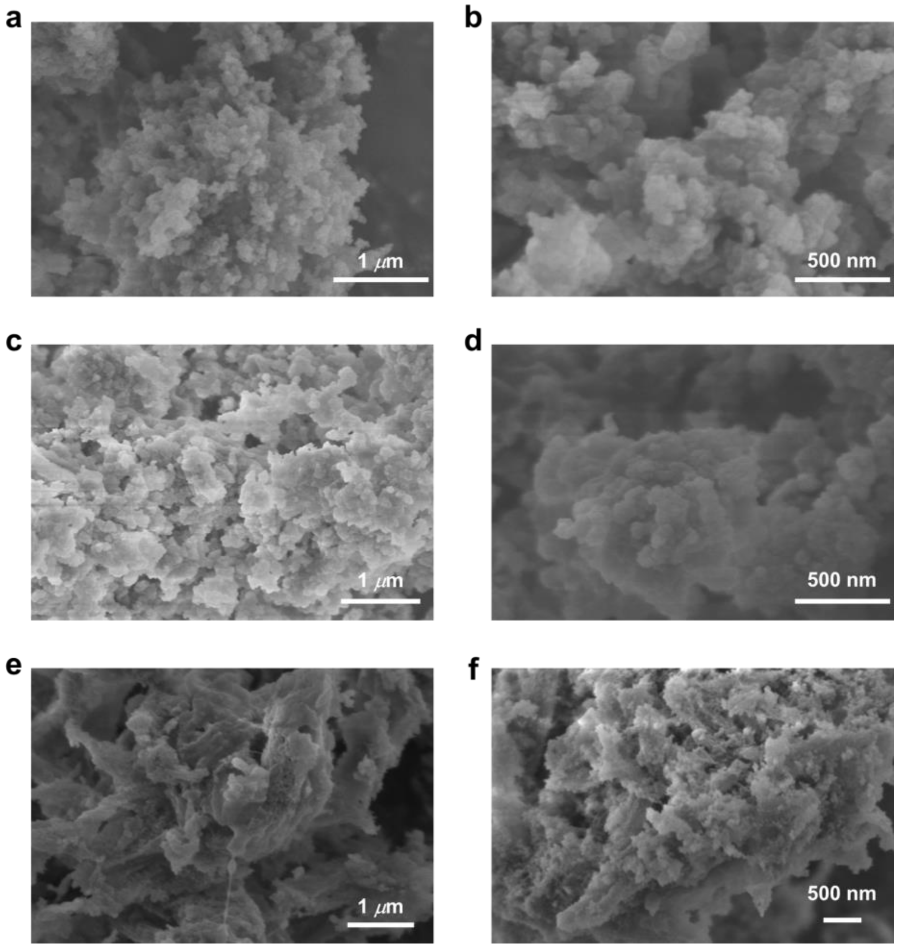


**Figure S46**. SEM images of ETTA-CHDA COF after sensing for $Fe^{3+}$ (a and b), PA (c and d) and PGA (e and f).

**Table S1.** Solvothermal conditions and corresponding results of crystallinity for TFB-CHDA COF, TFPB-CHDA COF, ETTA-CHDA COF, TFPPy-CHDA COF and TFPPery-CHDA COF.

| COFs | Ratio of Solvents ($v/v$, $V_{total}$ = 3 mL) | Catalyst (c/amount) | Temperature (℃) | Time (h) | Degree of Crystallinity |
|---|---|---|---|---|---|
| TFB-CHDA COF | DMAc | HAc (6 M/500 $\mu$L) | 120 | 72 | None |
| | DMAc | HAc (6 M/100 $\mu$L) | 120 | 72 | Weak |
| | *n*-BuOH | HAc (6 M/50 $\mu$L) | 120 | 72 | Weak |
| | DMAc/1,4-Dioxane (1/1) | HAc (6 M/50 $\mu$L) | 120 | 72 | Weak |
| | DMAc/1,4-Dioxane (4/1) | HAc (6 M/50 $\mu$L) | 120 | 72 | Strong |
| | DMAc/1,4-Dioxane (4/1) | HAc (6 M/300 $\mu$L) | 120 | 72 | Weak |
| | DMAc/1,4-Dioxane (8/1) | HAc (6 M/50 $\mu$L) | 120 | 72 | Weak |
| | ODCB/*n*-BuOH (1/1) | HAc (6 M/50 $\mu$L) | 120 | 72 | None |
| TFPB-CHDA COF | *n*-BuOH | HAc (6 M/50 $\mu$L) | 120 | 72 | Strong |
| | DMAc/1,4-Dioxane (4/1) | HAc (6 M/100 $\mu$L) | 120 | 72 | None |
| | DMAc/ODCB | HAc (6 M/50 $\mu$L) | 120 | 72 | None |
| | ODCB/*n*-BuOH (1/1) | HAc (6 M/50 $\mu$L) | 120 | 72 | Weak |
| | ODCB/*n*-BuOH (4/1) | HAc (6 M/50 $\mu$L) | 120 | 72 | Weak |
| ETTA-CHDA COF | *n*-BuOH | None | 80 | 72 | None |
| | *n*-BuOH | HAc (6 M/50 $\mu$L) | 80 | 72 | Weak |
| | *n*-BuOH | HAc (6 M/300 $\mu$L) | 80 | 72 | Weak |
| | Mesitylene/1,4-Dioxane (1/1) | None | 120 | 72 | Weak |
| | ODCB/*n*-BuOH (1/1) | None | 120 | 72 | None |
| | ODCB/*n*-BuOH (1/1) | HAc (6 M/300 $\mu$L) | 120 | 72 | None |

| | | | | | |
|---|---|---|---|---|---|
| | DMAc/Dioxane (1/1) | None | 120 | 72 | None |
| | DMAc/1,4-Dioxane (4/1) | HAc (6 M/100 $\mu$L ) | 90 | 72 | Weak |
| | DMAc/1,4-Dioxane (4/1) | HAc (6 M/60 $\mu$L ) | 90 | 72 | Strong |
| TFPPy-CHDA COF | Dioaxne | HAc (6 M/200 $\mu$L ) | 120 | 72 | None |
| | DMAc | HAc (6 M/200 $\mu$L) | 120 | 72 | Weak |
| | DMAc/1,4-Dioxane (1/1) | HAc (6 M/50 $\mu$L ) | 120 | 96 | Weak |
| | DMAc/1,4-Dioxane (9/1) | HAc (6 M/100 $\mu$L) | 120 | 72 | None |
| | DMAc/1,4-Dioxane (9/1) | HAc (6 M/100 $\mu$L) | 90 | 72 | Weak |
| | DMAc/1,4-Dioxane (9/1) | HAc (6 M/60 $\mu$L) | 120 | 72 | Weak |
| | 1,4-Dioxane /*n*-BuOH (4/1) | HAc (6 M/50 $\mu$L) | 120 | 96 | Weak |
| | 1,4-Dioxane /*n*-BuOH (1/4) | HAc (3 M/50 $\mu$L) | 120 | 72 | Strong |
| | 1,4-Dioxane /*n*-BuOH (1/4) | HAc (6 M/50 $\mu$L) | 120 | 96 | Weak |
| TFPPery-CHDA COF | *n*-BuOH | HAc (6 M/100 $\mu$L) | 120 | 72 | Weak |
| | 1,4-Dioxane | HAc (6 M/200 $\mu$L) | 120 | 72 | None |
| | DMAc/1,4-Dioxane (4/1) | HAc (6 M/400 $\mu$L) | 120 | 72 | Weak |
| | DMAc/1,4-Dioxane (4/1) | HAc (6 M/200 $\mu$L) | 120 | 72 | Strong |
| | DMAc/1,4-Dioxane (4/1) | HAc (6 M/100 $\mu$L) | 90 | 72 | Weak |
| | DMAc/1,4-Dioxane (4/1) | None | 120 | 72 | Weak |
| | DMAc/1,4-Dioxane (9/1) | HAc (6 M/200 $\mu$L) | 120 | 72 | Weak |
| | DMAc/1,4-Dioxane (4/1) | HAc (6 M/50 $\mu$L) | 150 | 72 | Weak |
| | DMAc/1,4-Dioxane (9/1) | HAc (6 M/100 $\mu$L) | 120 | 72 | Strong |
| | Meistylene/1,4-Dioxane (1/1) | HAc (6 M/100 $\mu$L) | 120 | 72 | None |
| | Meistylene/1,4-Dioxane (4/1) | HAc (6 M/100 $\mu$L) | 120 | 72 | Weak |

**Table S2**. Summary of the full-width at half-maximum (FWHM) of the first intense peaks in PXRD patterns of all the cyclohexane and phenylene-linked COFs.

| | First Intense Peak Position (degree) | FWHM (degree) |
|---|---|---|
| TFB-CHDA-COF | 4.09 | 0.25 |
| TFPB-CHDA-COF | 2.84 | 0.41 |
| ETTA-CHDA-COF | 2.77 | 0.45 |
| TFPPy-CHDA-COF | 4.10 | 0.68 |
| TFPPery-CHDA-COF | 4.22 | 0.43 |
| TFPPery-PDA-COF | 2.05 | 0.71 |

**Table S3.** Atomistic coordinates for the refined unit cell parameters for TFB-CHDA COF via Pawley refinement. Space group: $P1$; $a = b = 24.4657$ Å, $c = 7.0184$ Å, $\alpha = 90.0000^\circ$, $\beta = 90.0000^\circ$, and $\gamma = 120.1647^\circ$. Pawley-refined PXRD profile confirmed the correctness of the peak assignment as evident by a small difference with $R_{wp}$ and $R_p$ values of 0.75% and 1.56%, respectively.

| | | | |
|---|---|---|---|
| H1 | 0.4453 | 0.4993 | 0.1717 |
| H2 | 0.6198 | 0.6849 | 0.7664 |
| H3 | 0.4937 | 0.5688 | 0.0318 |
| H4 | 0.6686 | 0.7544 | 0.6295 |
| H5 | 0.3450 | 0.4078 | 0.7413 |
| H6 | 0.5177 | 0.5914 | 0.3528 |
| H7 | 0.3537 | 0.4003 | 0.9914 |
| H8 | 0.5318 | 0.5883 | 0.6002 |
| H9 | 0.3945 | 0.6991 | 0.9443 |
| H10 | 0.5747 | 0.8848 | 0.4688 |
| H11 | 0.2855 | 0.7718 | 0.9053 |
| H12 | 0.4730 | 0.9648 | 0.4217 |
| H13 | 0.3871 | 0.8838 | 0.7828 |
| H14 | 0.5849 | 0.0730 | 0.2611 |
| H15 | 0.3814 | 0.9098 | 0.2090 |
| H16 | 0.5705 | 0.0985 | 0.6782 |
| H17 | 0.3204 | 0.8890 | 0.0363 |
| H18 | 0.5109 | 0.0779 | 0.4986 |
| H19 | 0.4970 | 0.9087 | 0.8461 |
| H20 | 0.6915 | 0.0981 | 0.3518 |
| H21 | 0.4900 | 0.9220 | 0.0896 |
| H22 | 0.6785 | 0.1117 | 0.5937 |
| H23 | 0.2023 | 0.6611 | 0.9660 |
| H24 | 0.3847 | 0.8543 | 0.4647 |
| H25 | 0.1280 | 0.4794 | 0.0115 |
| H26 | 0.3046 | 0.6715 | 0.5252 |
| H27 | 0.9905 | 0.4645 | 0.6914 |
| H28 | 0.1593 | 0.6600 | 0.2396 |
| H29 | 0.9767 | 0.5425 | 0.8829 |
| H30 | 0.1593 | 0.7440 | 0.4650 |
| H31 | 0.6537 | 0.4048 | 0.9400 |
| H32 | 0.8278 | 0.5929 | 0.4935 |
| H33 | 0.4712 | 0.3017 | 0.0033 |
| H34 | 0.6468 | 0.4854 | 0.5572 |
| H35 | 0.4576 | 0.3978 | 0.1068 |

| | | | |
|---|---|---|---|
| H36 | 0.6357 | 0.5824 | 0.7003 |
| H37 | 0.4001 | 0.3457 | 0.8067 |
| H38 | 0.5737 | 0.5304 | 0.4124 |
| H39 | 0.4493 | 0.4159 | 0.6728 |
| H40 | 0.6218 | 0.5993 | 0.2699 |
| H41 | 0.5366 | 0.5119 | 0.8439 |
| H42 | 0.7141 | 0.6989 | 0.4456 |
| H43 | 0.5496 | 0.5075 | 0.0914 |
| H44 | 0.7237 | 0.6923 | 0.6958 |
| H45 | 0.4938 | 0.2150 | 0.9684 |
| H46 | 0.6726 | 0.4001 | 0.4963 |
| H47 | 0.5954 | 0.1347 | 0.0120 |
| H48 | 0.7806 | 0.3270 | 0.5430 |
| H49 | 0.3839 | 0.9909 | 0.8644 |
| H50 | 0.5766 | 0.1813 | 0.3401 |
| H51 | 0.6839 | 0.2454 | 0.9740 |
| H52 | 0.8645 | 0.4376 | 0.4745 |
| H53 | 0.7640 | 0.4285 | 0.9172 |
| H54 | 0.9390 | 0.6196 | 0.4308 |
| H55 | 0.8723 | 0.4462 | 0.7900 |
| H56 | 0.0503 | 0.6415 | 0.3338 |
| H57 | 0.9083 | 0.4435 | 0.2015 |
| H58 | 0.0809 | 0.6380 | 0.7598 |
| H59 | 0.8817 | 0.4945 | 0.1013 |
| H60 | 0.0594 | 0.6907 | 0.6510 |
| H61 | 0.8946 | 0.3590 | 0.7397 |
| H62 | 0.0697 | 0.5486 | 0.3109 |
| H63 | 0.9112 | 0.3586 | 0.9874 |
| H64 | 0.0916 | 0.5584 | 0.5532 |
| C1 | 0.0181 | 0.4848 | 0.9749 |
| C2 | 0.1977 | 0.6818 | 0.5267 |
| H65 | 0.0163 | 0.4587 | 0.1069 |
| H66 | 0.1966 | 0.6529 | 0.6475 |
| H67 | 0.0125 | 0.4130 | 0.8045 |
| H68 | 0.1860 | 0.6079 | 0.3305 |
| H69 | 0.9988 | 0.5526 | 0.1247 |
| H70 | 0.1778 | 0.7422 | 0.7105 |
| C3 | 0.4741 | 0.0282 | 0.0181 |
| C4 | 0.6642 | 0.2169 | 0.5044 |
| C5 | 0.4987 | 0.9984 | 0.9008 |
| C6 | 0.6917 | 0.1881 | 0.3943 |
| H71 | 0.4866 | 0.0255 | 0.1672 |
| H72 | 0.6758 | 0.2144 | 0.6557 |

| | | | |
|---|---|---|---|
| H73 | 0.5510 | 0.0239 | 0.9123 |
| H74 | 0.7437 | 0.2144 | 0.4163 |
| H75 | 0.4858 | 0.0013 | 0.7517 |
| H76 | 0.6835 | 0.1918 | 0.2408 |
| H77 | 0.3797 | 0.0066 | 0.1111 |
| H78 | 0.5685 | 0.1941 | 0.5828 |
| N1 | 0.3640 | 0.5180 | 0.9321 |
| N2 | 0.5404 | 0.7044 | 0.5119 |
| C7 | 0.2563 | 0.5563 | 0.9509 |
| C8 | 0.4335 | 0.7455 | 0.4925 |
| C9 | 0.3733 | 0.5767 | 0.9201 |
| C10 | 0.5499 | 0.7624 | 0.4785 |
| C11 | 0.4114 | 0.4967 | 0.8813 |
| C12 | 0.5875 | 0.6818 | 0.4727 |
| C13 | 0.4659 | 0.5164 | 0.0280 |
| C14 | 0.6411 | 0.7020 | 0.6233 |
| C15 | 0.3801 | 0.4233 | 0.8587 |
| C16 | 0.5553 | 0.6085 | 0.4619 |
| C17 | 0.3233 | 0.5988 | 0.9452 |
| C18 | 0.5009 | 0.7860 | 0.4864 |
| N3 | 0.3798 | 0.8129 | 0.9792 |
| N4 | 0.5690 | 0.0012 | 0.4648 |
| C19 | 0.3444 | 0.6657 | 0.9518 |
| C20 | 0.5242 | 0.8530 | 0.4750 |
| C21 | 0.3226 | 0.7625 | 0.9490 |
| C22 | 0.5094 | 0.9530 | 0.4497 |
| C23 | 0.4009 | 0.8819 | 0.9320 |
| C24 | 0.5935 | 0.0709 | 0.4144 |
| C25 | 0.3724 | 0.9142 | 0.0569 |
| C26 | 0.5625 | 0.1029 | 0.5266 |
| C27 | 0.4740 | 0.9261 | 0.9459 |
| C28 | 0.6663 | 0.1154 | 0.4434 |
| C29 | 0.3019 | 0.6902 | 0.9612 |
| C30 | 0.4841 | 0.8799 | 0.4679 |
| N5 | 0.0877 | 0.5333 | 0.9353 |
| N6 | 0.2671 | 0.7299 | 0.4826 |
| C31 | 0.2355 | 0.6441 | 0.9638 |
| C32 | 0.4166 | 0.8359 | 0.4703 |
| C33 | 0.1379 | 0.5262 | 0.9691 |
| C34 | 0.3161 | 0.7196 | 0.4954 |
| C35 | 0.9872 | 0.4396 | 0.8275 |
| C36 | 0.1608 | 0.6338 | 0.3664 |
| C37 | 0.9771 | 0.5166 | 0.0124 |

| | | | |
|---|---|---|---|
| C38 | 0.1566 | 0.7122 | 0.5824 |
| C39 | 0.2105 | 0.5771 | 0.9573 |
| C40 | 0.3894 | 0.7686 | 0.4837 |
| N7 | 0.5278 | 0.3954 | 0.9280 |
| N8 | 0.7037 | 0.5814 | 0.5083 |
| C41 | 0.6352 | 0.3544 | 0.9478 |
| C42 | 0.8109 | 0.5427 | 0.4892 |
| C43 | 0.5186 | 0.3374 | 0.9612 |
| C44 | 0.6941 | 0.5226 | 0.5204 |
| C45 | 0.4805 | 0.4177 | 0.9671 |
| C46 | 0.6566 | 0.6030 | 0.5595 |
| C47 | 0.4274 | 0.3981 | 0.8150 |
| C48 | 0.6016 | 0.5828 | 0.4144 |
| C49 | 0.5125 | 0.4909 | 0.9809 |
| C50 | 0.6882 | 0.6765 | 0.5794 |
| C51 | 0.5677 | 0.3138 | 0.9528 |
| C52 | 0.7440 | 0.5003 | 0.4949 |
| N9 | 0.4992 | 0.0985 | 0.9729 |
| N10 | 0.6873 | 0.2865 | 0.4575 |
| C53 | 0.5443 | 0.2468 | 0.9629 |
| C54 | 0.7227 | 0.4334 | 0.4884 |
| C55 | 0.5590 | 0.1467 | 0.9869 |
| C56 | 0.7442 | 0.3366 | 0.4935 |
| C57 | 0.4001 | 0.9869 | 0.0091 |
| C58 | 0.5908 | 0.1755 | 0.4844 |
| C59 | 0.5845 | 0.2199 | 0.9696 |
| C60 | 0.7651 | 0.4089 | 0.4792 |
| N11 | 0.8018 | 0.3702 | 0.9577 |
| N12 | 0.9794 | 0.5656 | 0.5032 |
| C61 | 0.6520 | 0.2639 | 0.9684 |
| C62 | 0.8316 | 0.4548 | 0.4765 |
| C63 | 0.7527 | 0.3803 | 0.9458 |
| C64 | 0.9292 | 0.5727 | 0.4714 |
| C65 | 0.8712 | 0.4188 | 0.9145 |
| C66 | 0.0487 | 0.6146 | 0.4633 |
| C67 | 0.9070 | 0.4683 | 0.0714 |
| C68 | 0.0840 | 0.6637 | 0.6257 |
| C69 | 0.9142 | 0.3896 | 0.8673 |
| C70 | 0.0907 | 0.5843 | 0.4242 |
| C71 | 0.6793 | 0.3312 | 0.9563 |
| C72 | 0.8566 | 0.5218 | 0.4827 |
| H79 | 0.2395 | 0.5061 | 0.9465 |
| H80 | 0.4149 | 0.6951 | 0.5015 |

| | | | |
|---|---|---|---|
| H81 | 0.4206 | 0.6140 | 0.8839 |
| H82 | 0.5973 | 0.7980 | 0.4364 |
| H83 | 0.4328 | 0.5179 | 0.7415 |
| H84 | 0.6100 | 0.7010 | 0.3320 |

**Table S4.** Atomistic coordinates for the refined unit cell parameters for TFPB-CHDA COF via Pawley refinement. Space group: $P1$; $a$ = 37.7014 Å, $b$ = 36.1649 Å, $c$ = 5.0332 Å, $\alpha$ = 90.0000°, $\beta$ = 90.0000°, and $\gamma$ = 119.9003°. Pawley-refined PXRD profile confirmed the correctness of the peak assignment as evident by a small difference with $R_{wp}$ and $R_p$ values of 1.94% and 3.72%, respectively.

| | | | |
|---|---|---|---|
| C1 | 0.3587 | 0.6402 | 0.8796 |
| C2 | 0.3758 | 0.6845 | 0.8538 |
| C3 | 0.4650 | 0.5687 | 0.9354 |
| N4 | 0.4572 | 0.5350 | 0.7988 |
| H5 | 0.4105 | 0.7063 | 0.8646 |
| H6 | 0.4936 | 0.5850 | 1.0654 |
| C7 | 0.3509 | 0.7027 | 0.8146 |
| C8 | 0.3082 | 0.6759 | 0.8101 |
| C9 | 0.4272 | 0.8826 | 0.5810 |
| N10 | 0.4625 | 0.9098 | 0.6811 |
| H11 | 0.2875 | 0.6902 | 0.7802 |
| H12 | 0.4112 | 0.8938 | 0.4364 |
| C13 | 0.2901 | 0.6314 | 0.8421 |
| C14 | 0.3159 | 0.6140 | 0.8744 |
| C15 | 0.1141 | 0.5209 | 0.8142 |
| N16 | 0.0896 | 0.5363 | 0.7951 |
| H17 | 0.3022 | 0.5780 | 0.8969 |
| H18 | 0.1011 | 0.4849 | 0.8217 |
| C19 | 0.4836 | 0.5162 | 0.7992 |
| C20 | 0.4611 | 0.4729 | 0.6585 |
| C21 | 0.5241 | 0.5458 | 0.6550 |
| H22 | 0.4522 | 0.4775 | 0.4495 |
| H23 | 0.4322 | 0.4505 | 0.7745 |
| H24 | 0.5413 | 0.5771 | 0.7689 |
| H25 | 0.5172 | 0.5525 | 0.4452 |
| H26 | 0.4904 | 0.5113 | 1.0133 |
| C27 | 0.4629 | 0.9776 | 0.7298 |
| H28 | 0.4602 | 0.9725 | 0.9544 |
| H29 | 0.4310 | 0.9637 | 0.6400 |
| C30 | 0.9791 | 0.5117 | 0.7725 |
| C31 | 0.9680 | 0.4906 | 0.4976 |
| C32 | 0.9875 | 0.4626 | 0.4587 |
| H33 | 0.9653 | 0.4859 | 0.9322 |
| H34 | 0.9661 | 0.5341 | 0.7947 |
| H35 | 0.9803 | 0.4485 | 0.2497 |

| | | | |
|---|---|---|---|
| H36 | 0.9743 | 0.4356 | 0.6106 |
| H37 | 0.9796 | 0.5164 | 0.3372 |
| C38 | 0.5311 | 0.0459 | 0.7763 |
| C39 | 0.4874 | 0.0258 | 0.6701 |
| H40 | 0.4884 | 0.0309 | 0.4458 |
| H41 | 0.4711 | 0.0417 | 0.7679 |
| H42 | 0.5306 | 0.0436 | 1.0026 |
| C43 | 0.0343 | 0.4886 | 0.4958 |
| C44 | 0.0453 | 0.5095 | 0.7710 |
| C45 | 0.0259 | 0.5376 | 0.8093 |
| H46 | 0.0475 | 0.4665 | 0.4735 |
| H47 | 0.0478 | 0.5144 | 0.3359 |
| H48 | 0.0394 | 0.5647 | 0.6579 |
| H49 | 0.0328 | 0.5517 | 1.0186 |
| H50 | 0.0334 | 0.4837 | 0.9309 |
| C51 | 0.4845 | 0.9550 | 0.6102 |
| C52 | 0.5283 | 0.9750 | 0.7174 |
| H53 | 0.5444 | 0.9589 | 0.6194 |
| H54 | 0.5275 | 0.9700 | 0.9418 |
| H55 | 0.4855 | 0.9583 | 0.3844 |
| C56 | 0.6542 | 0.3584 | 0.4231 |
| C57 | 0.6373 | 0.3144 | 0.4605 |
| C58 | 0.5472 | 0.4291 | 0.3619 |
| N59 | 0.5550 | 0.4630 | 0.4963 |
| H60 | 0.6026 | 0.2925 | 0.4570 |
| H61 | 0.5185 | 0.4125 | 0.2335 |
| C62 | 0.6625 | 0.2966 | 0.5027 |
| C63 | 0.7052 | 0.3235 | 0.4979 |
| C64 | 0.5882 | 0.1186 | 0.7852 |
| N65 | 0.5523 | 0.0905 | 0.6958 |
| H66 | 0.7262 | 0.3095 | 0.5296 |
| H67 | 0.6051 | 0.1084 | 0.9293 |
| C68 | 0.7230 | 0.3677 | 0.4544 |
| C69 | 0.6969 | 0.3848 | 0.4194 |
| C70 | 0.8989 | 0.4788 | 0.4570 |
| N71 | 0.9236 | 0.4637 | 0.4755 |
| H72 | 0.7105 | 0.4205 | 0.3873 |
| H73 | 0.9117 | 0.5148 | 0.4465 |
| C74 | 0.5286 | 0.4818 | 0.4954 |
| C75 | 0.5511 | 0.5251 | 0.6359 |
| C76 | 0.4882 | 0.4522 | 0.6395 |
| H77 | 0.5801 | 0.5472 | 0.5202 |
| H78 | 0.5598 | 0.5206 | 0.8458 |

| | | | |
|---|---|---|---|
| H79 | 0.4951 | 0.4453 | 0.8486 |
| H80 | 0.4707 | 0.4209 | 0.5254 |
| H81 | 0.5218 | 0.4866 | 0.2812 |
| C82 | 0.5528 | 0.0232 | 0.6594 |
| H83 | 0.5846 | 0.0373 | 0.7507 |
| H84 | 0.5557 | 0.0281 | 0.4347 |
| C85 | 0.3854 | 0.6212 | 0.9007 |
| C86 | 0.4229 | 0.6426 | 1.0371 |
| C87 | 0.4486 | 0.6251 | 1.0498 |
| C88 | 0.4374 | 0.5862 | 0.9246 |
| C89 | 0.3999 | 0.5647 | 0.7887 |
| C90 | 0.3742 | 0.5820 | 0.7774 |
| C91 | 0.1587 | 0.5495 | 0.8265 |
| C92 | 0.1760 | 0.5939 | 0.8148 |
| C93 | 0.2186 | 0.6204 | 0.8207 |
| C94 | 0.2446 | 0.6032 | 0.8381 |
| C95 | 0.2268 | 0.5585 | 0.8506 |
| C96 | 0.1843 | 0.5320 | 0.8446 |
| C97 | 0.3697 | 0.7492 | 0.7662 |
| C98 | 0.3503 | 0.7650 | 0.6007 |
| C99 | 0.3690 | 0.8086 | 0.5436 |
| C100 | 0.4073 | 0.8371 | 0.6518 |
| C101 | 0.4267 | 0.8217 | 0.8196 |
| C102 | 0.4081 | 0.7781 | 0.8758 |
| C103 | 0.5750 | 0.4117 | 0.3736 |
| C104 | 0.6128 | 0.4339 | 0.5046 |
| C105 | 0.6387 | 0.4168 | 0.5172 |
| C106 | 0.6272 | 0.3772 | 0.4001 |
| C107 | 0.5894 | 0.3551 | 0.2686 |
| C108 | 0.5636 | 0.3724 | 0.2546 |
| C109 | 0.7685 | 0.3961 | 0.4504 |
| C110 | 0.7861 | 0.4407 | 0.4331 |
| C111 | 0.8285 | 0.4674 | 0.4329 |
| C112 | 0.8544 | 0.4501 | 0.4495 |
| C113 | 0.8372 | 0.4057 | 0.4655 |
| C114 | 0.7947 | 0.3790 | 0.4658 |
| C115 | 0.6440 | 0.2504 | 0.5641 |
| C116 | 0.6645 | 0.2360 | 0.7309 |
| C117 | 0.6463 | 0.1929 | 0.7999 |
| C118 | 0.6074 | 0.1635 | 0.7028 |
| C119 | 0.5870 | 0.1776 | 0.5340 |
| C120 | 0.6052 | 0.2207 | 0.4658 |
| H121 | 0.4323 | 0.6744 | 1.1379 |

| | | | |
|---|---|---|---|
| H122 | 0.4788 | 0.6426 | 1.1624 |
| H123 | 0.3905 | 0.5329 | 0.6869 |
| H124 | 0.3437 | 0.5643 | 0.6674 |
| H125 | 0.1553 | 0.6084 | 0.8004 |
| H126 | 0.2325 | 0.6566 | 0.8114 |
| H127 | 0.2471 | 0.5435 | 0.8658 |
| H128 | 0.1704 | 0.4958 | 0.8546 |
| H129 | 0.3190 | 0.7419 | 0.5126 |
| H130 | 0.3529 | 0.8210 | 0.4084 |
| H131 | 0.4577 | 0.8447 | 0.9108 |
| H132 | 0.4240 | 0.7657 | 1.0116 |
| H133 | 0.6223 | 0.4661 | 0.6012 |
| H134 | 0.6695 | 0.4351 | 0.6232 |
| H135 | 0.5796 | 0.3228 | 0.1724 |
| H136 | 0.5330 | 0.3543 | 0.1458 |
| H137 | 0.7651 | 0.4549 | 0.4191 |
| H138 | 0.8423 | 0.5035 | 0.4191 |
| H139 | 0.8580 | 0.3913 | 0.4785 |
| H140 | 0.7809 | 0.3430 | 0.4787 |
| H141 | 0.6962 | 0.2598 | 0.8101 |
| H142 | 0.6631 | 0.1816 | 0.9360 |
| H143 | 0.5555 | 0.1539 | 0.4518 |
| H144 | 0.5884 | 0.2321 | 0.3292 |

**Table S5.** Atomistic coordinates for the refined unit cell parameters for ETTA-CHDA COF via Pawley refinement. Space group: *P*1; $a = b = 38.0273$ Å, $c = 5.0243$ Å, $\alpha = 89.9399^\circ$, $\beta = 89.9455^\circ$, and $\gamma = 121.6771^\circ$. Pawley-refined PXRD profile confirmed the correctness of the peak assignment as evident by a small difference with $R_{wp}$ and $R_p$ values of 2.43% and 3.14%, respectively.

| | | | |
|---|---|---|---|
| C1 | 0.4800 | 0.4848 | 0.4365 |
| C2 | 0.4450 | 0.4927 | 0.4316 |
| C3 | 0.4118 | 0.4694 | 0.2631 |
| C4 | 0.3792 | 0.4765 | 0.2496 |
| C5 | 0.3789 | 0.5065 | 0.4095 |
| C6 | 0.4111 | 0.5289 | 0.5845 |
| C7 | 0.4435 | 0.5216 | 0.5974 |
| H8 | 0.4690 | 0.5396 | 0.7454 |
| C9 | 0.4682 | 0.4411 | 0.4425 |
| C10 | 0.4419 | 0.4158 | 0.6423 |
| C11 | 0.4335 | 0.3759 | 0.6678 |
| C12 | 0.4507 | 0.3604 | 0.4900 |
| C13 | 0.4745 | 0.3845 | 0.2768 |
| C14 | 0.4830 | 0.4246 | 0.2542 |
| C15 | 0.3448 | 0.5149 | 0.3910 |
| C16 | 0.4449 | 0.3190 | 0.5380 |
| H17 | 0.4111 | 0.4444 | 0.1352 |
| H18 | 0.3529 | 0.4579 | 0.1082 |
| H19 | 0.4107 | 0.5531 | 0.7166 |
| H20 | 0.4272 | 0.4279 | 0.7857 |
| H21 | 0.4126 | 0.3558 | 0.8335 |
| H22 | 0.4867 | 0.3713 | 0.1230 |
| H23 | 0.5022 | 0.4439 | 0.0804 |
| C24 | 0.7185 | 0.1968 | 0.6037 |
| C25 | 0.7553 | 0.1987 | 0.4572 |
| C26 | 0.7942 | 0.2387 | 0.5152 |
| C27 | 0.7898 | 0.2751 | 0.4335 |
| C28 | 0.7535 | 0.2737 | 0.5804 |
| C29 | 0.7144 | 0.2337 | 0.5264 |
| H30 | 0.7241 | 0.1979 | 0.8272 |
| N31 | 0.8271 | 0.3120 | 0.4967 |
| N32 | 0.6803 | 0.1601 | 0.5434 |
| H33 | 0.7493 | 0.1960 | 0.2337 |
| H34 | 0.7596 | 0.1726 | 0.5263 |
| H35 | 0.8204 | 0.2396 | 0.3999 |

| | | | |
|---|---|---|---|
| H36 | 0.8011 | 0.2410 | 0.7373 |
| H37 | 0.7842 | 0.2739 | 0.2098 |
| H38 | 0.7496 | 0.3001 | 0.5100 |
| H39 | 0.7596 | 0.2766 | 0.8037 |
| H40 | 0.6886 | 0.2331 | 0.6456 |
| H41 | 0.7071 | 0.2322 | 0.3051 |
| H42 | 0.3498 | 0.5442 | 0.2946 |
| H43 | 0.4130 | 0.2919 | 0.5644 |
| C44 | 0.5152 | 0.9952 | 0.4365 |
| C45 | 0.5073 | 0.9524 | 0.4316 |
| C46 | 0.5306 | 0.9423 | 0.2631 |
| C47 | 0.5235 | 0.9027 | 0.2496 |
| C48 | 0.4935 | 0.8724 | 0.4095 |
| C49 | 0.4711 | 0.8822 | 0.5845 |
| C50 | 0.4784 | 0.9219 | 0.5974 |
| H51 | 0.4604 | 0.9294 | 0.7454 |
| C52 | 0.5589 | 0.0271 | 0.4425 |
| C53 | 0.5842 | 0.0261 | 0.6423 |
| C54 | 0.6242 | 0.0577 | 0.6678 |
| C55 | 0.6396 | 0.0903 | 0.4900 |
| C56 | 0.6155 | 0.0899 | 0.2768 |
| C57 | 0.5754 | 0.0584 | 0.2542 |
| C58 | 0.4851 | 0.8299 | 0.3910 |
| C59 | 0.6810 | 0.1260 | 0.5380 |
| H60 | 0.5556 | 0.9668 | 0.1352 |
| H61 | 0.5421 | 0.8950 | 0.1082 |
| H62 | 0.4469 | 0.8576 | 0.7166 |
| H63 | 0.5721 | 0.9993 | 0.7857 |
| H64 | 0.6442 | 0.0569 | 0.8335 |
| H65 | 0.6287 | 0.1154 | 0.1230 |
| H66 | 0.5561 | 0.0583 | 0.0804 |
| C67 | 0.8032 | 0.5217 | 0.6037 |
| C68 | 0.8013 | 0.5565 | 0.4572 |
| C69 | 0.7613 | 0.5555 | 0.5152 |
| C70 | 0.7249 | 0.5146 | 0.4335 |
| C71 | 0.7263 | 0.4798 | 0.5804 |
| C72 | 0.7663 | 0.4807 | 0.5264 |
| H73 | 0.8021 | 0.5262 | 0.8272 |
| N74 | 0.6880 | 0.5151 | 0.4967 |
| N75 | 0.8399 | 0.5202 | 0.5434 |
| H76 | 0.8041 | 0.5533 | 0.2337 |
| H77 | 0.8275 | 0.5870 | 0.5263 |
| H78 | 0.7604 | 0.5809 | 0.3999 |

| | | | |
|---|---|---|---|
| H79 | 0.7590 | 0.5601 | 0.7373 |
| H80 | 0.7261 | 0.5104 | 0.2098 |
| H81 | 0.7000 | 0.4495 | 0.5100 |
| H82 | 0.7234 | 0.4829 | 0.8037 |
| H83 | 0.7669 | 0.4555 | 0.6456 |
| H84 | 0.7678 | 0.4749 | 0.3051 |
| H85 | 0.4558 | 0.8056 | 0.2946 |
| H86 | 0.7081 | 0.1212 | 0.5644 |
| C87 | 0.0048 | 0.5200 | 0.4365 |
| C88 | 0.0476 | 0.5550 | 0.4316 |
| C89 | 0.0577 | 0.5883 | 0.2631 |
| C90 | 0.0973 | 0.6208 | 0.2496 |
| C91 | 0.1276 | 0.6211 | 0.4095 |
| C92 | 0.1178 | 0.5890 | 0.5845 |
| C93 | 0.0781 | 0.5565 | 0.5974 |
| H94 | 0.0706 | 0.5310 | 0.7454 |
| C95 | 0.9729 | 0.5318 | 0.4425 |
| C96 | 0.9739 | 0.5581 | 0.6423 |
| C97 | 0.9423 | 0.5665 | 0.6678 |
| C98 | 0.9097 | 0.5493 | 0.4900 |
| C99 | 0.9101 | 0.5255 | 0.2768 |
| C100 | 0.9416 | 0.5170 | 0.2542 |
| C101 | 0.1701 | 0.6552 | 0.3910 |
| C102 | 0.8740 | 0.5551 | 0.5380 |
| H103 | 0.0332 | 0.5889 | 0.1352 |
| H104 | 0.1050 | 0.6471 | 0.1082 |
| H105 | 0.1424 | 0.5893 | 0.7166 |
| H106 | 0.0007 | 0.5728 | 0.7857 |
| H107 | 0.9431 | 0.5874 | 0.8335 |
| H108 | 0.8846 | 0.5133 | 0.1230 |
| H109 | 0.9417 | 0.4978 | 0.0804 |
| C110 | 0.4783 | 0.2815 | 0.6037 |
| C111 | 0.4435 | 0.2447 | 0.4572 |
| C112 | 0.4446 | 0.2058 | 0.5152 |
| C113 | 0.4854 | 0.2102 | 0.4335 |
| C114 | 0.5202 | 0.2465 | 0.5804 |
| C115 | 0.5193 | 0.2856 | 0.5264 |
| H116 | 0.4738 | 0.2759 | 0.8272 |
| N117 | 0.4850 | 0.1729 | 0.4967 |
| N118 | 0.4798 | 0.3197 | 0.5434 |
| H119 | 0.4467 | 0.2508 | 0.2337 |
| H120 | 0.4130 | 0.2405 | 0.5263 |
| H121 | 0.4192 | 0.1796 | 0.3999 |

| | | | |
|---|---|---|---|
| H122 | 0.4399 | 0.1989 | 0.7373 |
| H123 | 0.4896 | 0.2158 | 0.2098 |
| H124 | 0.5505 | 0.2505 | 0.5100 |
| H125 | 0.5171 | 0.2404 | 0.8037 |
| H126 | 0.5445 | 0.3114 | 0.6456 |
| H127 | 0.5251 | 0.2929 | 0.3051 |
| H128 | 0.1944 | 0.6502 | 0.2946 |
| H129 | 0.8789 | 0.5870 | 0.5644 |
| C130 | 0.5200 | 0.5152 | 0.4365 |
| C131 | 0.5550 | 0.5073 | 0.4316 |
| C132 | 0.5883 | 0.5306 | 0.2631 |
| C133 | 0.6208 | 0.5235 | 0.2496 |
| C134 | 0.6211 | 0.4935 | 0.4095 |
| C135 | 0.5890 | 0.4711 | 0.5845 |
| C136 | 0.5565 | 0.4784 | 0.5974 |
| H137 | 0.5310 | 0.4604 | 0.7454 |
| C138 | 0.5318 | 0.5589 | 0.4425 |
| C139 | 0.5581 | 0.5842 | 0.6423 |
| C140 | 0.5665 | 0.6242 | 0.6678 |
| C141 | 0.5493 | 0.6396 | 0.4900 |
| C142 | 0.5255 | 0.6155 | 0.2768 |
| C143 | 0.5170 | 0.5754 | 0.2542 |
| C144 | 0.6552 | 0.4851 | 0.3910 |
| C145 | 0.5551 | 0.6810 | 0.5380 |
| H146 | 0.5889 | 0.5556 | 0.1352 |
| H147 | 0.6471 | 0.5421 | 0.1082 |
| H148 | 0.5893 | 0.4469 | 0.7166 |
| H149 | 0.5728 | 0.5721 | 0.7857 |
| H150 | 0.5874 | 0.6442 | 0.8335 |
| H151 | 0.5133 | 0.6287 | 0.1230 |
| H152 | 0.4978 | 0.5561 | 0.0804 |
| C153 | 0.2815 | 0.8032 | 0.6037 |
| C154 | 0.2447 | 0.8013 | 0.4572 |
| C155 | 0.2058 | 0.7613 | 0.5152 |
| C156 | 0.2102 | 0.7249 | 0.4335 |
| C157 | 0.2465 | 0.7263 | 0.5804 |
| C158 | 0.2856 | 0.7663 | 0.5264 |
| H159 | 0.2759 | 0.8021 | 0.8272 |
| N160 | 0.1729 | 0.6880 | 0.4967 |
| N161 | 0.3197 | 0.8399 | 0.5434 |
| H162 | 0.2508 | 0.8041 | 0.2337 |
| H163 | 0.2405 | 0.8275 | 0.5263 |
| H164 | 0.1796 | 0.7604 | 0.3999 |

| | | | |
|---|---|---|---|
| H165 | 0.1989 | 0.7590 | 0.7373 |
| H166 | 0.2158 | 0.7261 | 0.2098 |
| H167 | 0.2505 | 0.7000 | 0.5100 |
| H168 | 0.2404 | 0.7234 | 0.8037 |
| H169 | 0.3114 | 0.7669 | 0.6456 |
| H170 | 0.2929 | 0.7678 | 0.3051 |
| H171 | 0.6502 | 0.4558 | 0.2946 |
| H172 | 0.5870 | 0.7081 | 0.5644 |
| C173 | 0.4848 | 0.0048 | 0.4365 |
| C174 | 0.4927 | 0.0476 | 0.4316 |
| C175 | 0.4694 | 0.0577 | 0.2631 |
| C176 | 0.4765 | 0.0973 | 0.2496 |
| C177 | 0.5065 | 0.1276 | 0.4095 |
| C178 | 0.5289 | 0.1178 | 0.5845 |
| C179 | 0.5216 | 0.0781 | 0.5974 |
| H180 | 0.5396 | 0.0706 | 0.7454 |
| C181 | 0.4411 | 0.9729 | 0.4425 |
| C182 | 0.4158 | 0.9739 | 0.6423 |
| C183 | 0.3759 | 0.9423 | 0.6678 |
| C184 | 0.3604 | 0.9097 | 0.4900 |
| C185 | 0.3845 | 0.9101 | 0.2768 |
| C186 | 0.4246 | 0.9416 | 0.2542 |
| C187 | 0.5149 | 0.1701 | 0.3910 |
| C188 | 0.3190 | 0.8740 | 0.5380 |
| H189 | 0.4444 | 0.0332 | 0.1352 |
| H190 | 0.4579 | 0.1050 | 0.1082 |
| H191 | 0.5531 | 0.1424 | 0.7166 |
| H192 | 0.4279 | 0.0007 | 0.7857 |
| H193 | 0.3558 | 0.9431 | 0.8335 |
| H194 | 0.3713 | 0.8846 | 0.1230 |
| H195 | 0.4439 | 0.9417 | 0.0804 |
| C196 | 0.1968 | 0.4783 | 0.6037 |
| C197 | 0.1987 | 0.4435 | 0.4572 |
| C198 | 0.2387 | 0.4446 | 0.5152 |
| C199 | 0.2751 | 0.4854 | 0.4335 |
| C200 | 0.2737 | 0.5202 | 0.5804 |
| C201 | 0.2337 | 0.5193 | 0.5264 |
| H202 | 0.1979 | 0.4738 | 0.8272 |
| N203 | 0.3120 | 0.4850 | 0.4967 |
| N204 | 0.1601 | 0.4798 | 0.5434 |
| H205 | 0.1960 | 0.4467 | 0.2337 |
| H206 | 0.1726 | 0.4130 | 0.5263 |
| H207 | 0.2396 | 0.4192 | 0.3999 |

| | | | |
|---|---|---|---|
| H208 | 0.2410 | 0.4399 | 0.7373 |
| H209 | 0.2739 | 0.4896 | 0.2098 |
| H210 | 0.3001 | 0.5505 | 0.5100 |
| H211 | 0.2766 | 0.5171 | 0.8037 |
| H212 | 0.2331 | 0.5445 | 0.6456 |
| H213 | 0.2322 | 0.5251 | 0.3051 |
| H214 | 0.5442 | 0.1944 | 0.2946 |
| H215 | 0.2919 | 0.8789 | 0.5644 |
| C216 | 0.9952 | 0.4800 | 0.4365 |
| C217 | 0.9524 | 0.4450 | 0.4316 |
| C218 | 0.9423 | 0.4118 | 0.2631 |
| C219 | 0.9027 | 0.3792 | 0.2496 |
| C220 | 0.8724 | 0.3789 | 0.4095 |
| C221 | 0.8822 | 0.4111 | 0.5845 |
| C222 | 0.9219 | 0.4435 | 0.5974 |
| H223 | 0.9294 | 0.4690 | 0.7454 |
| C224 | 0.0271 | 0.4682 | 0.4425 |
| C225 | 0.0261 | 0.4419 | 0.6423 |
| C226 | 0.0577 | 0.4335 | 0.6678 |
| C227 | 0.0903 | 0.4507 | 0.4900 |
| C228 | 0.0899 | 0.4745 | 0.2768 |
| C229 | 0.0584 | 0.4830 | 0.2542 |
| C230 | 0.8299 | 0.3448 | 0.3910 |
| C231 | 0.1260 | 0.4449 | 0.5380 |
| H232 | 0.9668 | 0.4111 | 0.1352 |
| H233 | 0.8950 | 0.3529 | 0.1082 |
| H234 | 0.8576 | 0.4107 | 0.7166 |
| H235 | 0.9993 | 0.4272 | 0.7857 |
| H236 | 0.0569 | 0.4126 | 0.8335 |
| H237 | 0.1154 | 0.4867 | 0.1230 |
| H238 | 0.0583 | 0.5022 | 0.0804 |
| C239 | 0.5217 | 0.7185 | 0.6037 |
| C240 | 0.5565 | 0.7553 | 0.4572 |
| C241 | 0.5555 | 0.7942 | 0.5152 |
| C242 | 0.5146 | 0.7898 | 0.4335 |
| C243 | 0.4798 | 0.7535 | 0.5804 |
| C244 | 0.4807 | 0.7144 | 0.5264 |
| H245 | 0.5262 | 0.7241 | 0.8272 |
| N246 | 0.5151 | 0.8271 | 0.4967 |
| N247 | 0.5202 | 0.6803 | 0.5434 |
| H248 | 0.5533 | 0.7493 | 0.2337 |
| H249 | 0.5870 | 0.7596 | 0.5263 |
| H250 | 0.5809 | 0.8204 | 0.3999 |

| | | | |
|---|---|---|---|
| H251 | 0.5601 | 0.8011 | 0.7373 |
| H252 | 0.5104 | 0.7842 | 0.2098 |
| H253 | 0.4495 | 0.7496 | 0.5100 |
| H254 | 0.4829 | 0.7596 | 0.8037 |
| H255 | 0.4555 | 0.6886 | 0.6456 |
| H256 | 0.4749 | 0.7071 | 0.3051 |
| H257 | 0.8056 | 0.3498 | 0.2946 |
| H258 | 0.1212 | 0.4130 | 0.5644 |

**Table S6.** Atomistic coordinates for the refined unit cell parameters for TFPPy-CHDA COF via Pawley refinement. Space group: *P*1; $a$ = 29.8609 Å, $b$ = 37.9624 Å, $c$ = 4.9700 Å, $\alpha$ = 60.2677°, $\beta$ = 90.0467°, and $\gamma$ = 91.3290°. Pawley-refined PXRD profile confirmed the correctness of the peak assignment as evident by a small difference with $R_{wp}$ and $R_p$ values of 0.65% and 0.99%, respectively.

| | | | |
|---|---|---|---|
| C1 | 0.0349 | 0.9300 | 0.4609 |
| C2 | 0.0342 | 0.9692 | 0.4319 |
| C3 | 0.0686 | 0.9975 | 0.2790 |
| C4 | 0.9157 | 0.8948 | 0.7132 |
| C5 | 0.9159 | 0.8523 | 0.8757 |
| C6 | 0.8783 | 0.8303 | 0.8746 |
| C7 | 0.8395 | 0.8505 | 0.7150 |
| C8 | 0.8382 | 0.8928 | 0.5689 |
| C9 | 0.8760 | 0.9148 | 0.5649 |
| C10 | 0.1930 | 0.8493 | 0.3607 |
| C11 | 0.7720 | 0.7699 | 0.6773 |
| C12 | 0.7543 | 0.7914 | 0.3449 |
| C13 | 0.7168 | 0.7666 | 0.3086 |
| N14 | 0.6985 | 0.7029 | 0.3596 |
| H15 | 0.6739 | 0.3289 | 0.0773 |
| H16 | 0.4028 | 0.5134 | 0.8813 |
| H17 | 0.5582 | 0.6620 | 0.0401 |
| H18 | 0.6276 | 0.7002 | 0.0494 |
| H19 | 0.6958 | 0.5849 | 0.6309 |
| H20 | 0.6258 | 0.5466 | 0.6400 |
| H21 | 0.7828 | 0.7955 | 0.1791 |
| H22 | 0.7048 | 0.7827 | 0.0587 |
| C23 | 0.5323 | 0.4308 | 0.3932 |
| C24 | 0.5349 | 0.4690 | 0.3803 |
| C25 | 0.5716 | 0.4953 | 0.2451 |
| C26 | 0.4127 | 0.3992 | 0.6546 |
| C27 | 0.4117 | 0.3567 | 0.8286 |
| C28 | 0.3728 | 0.3354 | 0.8397 |
| C29 | 0.3343 | 0.3562 | 0.6800 |
| C30 | 0.3345 | 0.3986 | 0.5210 |
| C31 | 0.3735 | 0.4199 | 0.5050 |
| C32 | 0.6731 | 0.3349 | 0.2808 |
| C33 | 0.2643 | 0.2758 | 0.6593 |
| C34 | 0.2467 | 0.2971 | 0.3266 |
| C35 | 0.2090 | 0.2723 | 0.2922 |

| | | | |
|---|---|---|---|
| N36 | 0.1906 | 0.2084 | 0.3479 |
| H37 | 0.2275 | 0.8636 | 0.3382 |
| H38 | 0.8997 | 0.0080 | 0.9509 |
| H39 | 0.0487 | 0.1629 | 0.0822 |
| H40 | 0.1174 | 0.2033 | 0.0665 |
| H41 | 0.1909 | 0.0903 | 0.6256 |
| H42 | 0.1216 | 0.0499 | 0.6606 |
| H43 | 0.2752 | 0.3008 | 0.1616 |
| H44 | 0.1970 | 0.2882 | 0.0422 |
| C45 | 0.9579 | 0.9184 | 0.6615 |
| C46 | 0.9600 | 0.9545 | 0.6785 |
| C47 | 0.9260 | 0.9648 | 0.8221 |
| C48 | 0.0762 | 0.9118 | 0.4096 |
| C49 | 0.0735 | 0.8864 | 0.2782 |
| C50 | 0.1111 | 0.8653 | 0.2677 |
| C51 | 0.1527 | 0.8706 | 0.3753 |
| C52 | 0.1559 | 0.8966 | 0.4986 |
| C53 | 0.1179 | 0.9163 | 0.5221 |
| C54 | 0.8010 | 0.8282 | 0.6879 |
| C55 | 0.2263 | 0.7912 | 0.3676 |
| C56 | 0.2561 | 0.7786 | 0.6524 |
| C57 | 0.2950 | 0.7524 | 0.6569 |
| N58 | 0.3145 | 0.6925 | 0.6493 |
| H59 | 0.2602 | 0.3483 | 0.6272 |
| H60 | 0.6050 | 0.5524 | 0.1434 |
| H61 | 0.4624 | 0.6193 | 0.8596 |
| H62 | 0.3959 | 0.6602 | 0.8583 |
| H63 | 0.3129 | 0.6038 | 0.4406 |
| H64 | 0.3804 | 0.5681 | 0.3944 |
| H65 | 0.2350 | 0.7607 | 0.8705 |
| H66 | 0.3157 | 0.7429 | 0.8745 |
| C67 | 0.4558 | 0.4217 | 0.5971 |
| C68 | 0.4601 | 0.4572 | 0.6183 |
| C69 | 0.4269 | 0.4685 | 0.7602 |
| C70 | 0.5709 | 0.4113 | 0.3255 |
| C71 | 0.5634 | 0.3885 | 0.1759 |
| C72 | 0.5967 | 0.3637 | 0.1638 |
| C73 | 0.6388 | 0.3617 | 0.2941 |
| C74 | 0.6473 | 0.3854 | 0.4338 |
| C75 | 0.6137 | 0.4099 | 0.4494 |
| C76 | 0.2948 | 0.3342 | 0.6628 |
| C77 | 0.7361 | 0.2909 | 0.5066 |
| C78 | 0.7680 | 0.2791 | 0.7785 |

| | | | |
|---|---|---|---|
| C79 | 0.8040 | 0.2501 | 0.7881 |
| N80 | 0.8178 | 0.1886 | 0.7881 |
| H81 | 0.7661 | 0.8417 | 0.6479 |
| H82 | 0.0985 | 0.0563 | 0.1547 |
| H83 | 0.9621 | 0.1126 | 0.9604 |
| H84 | 0.8979 | 0.1537 | 0.9859 |
| H85 | 0.8083 | 0.0994 | 0.5857 |
| H86 | 0.8736 | 0.0636 | 0.5108 |
| H87 | 0.7480 | 0.2638 | 1.0035 |
| H88 | 0.8258 | 0.2406 | 1.0010 |
| C89 | 0.9623 | 0.0681 | 0.6571 |
| C90 | 0.9621 | 0.0294 | 0.6767 |
| C91 | 0.9274 | 0.0012 | 0.8252 |
| C92 | 0.0805 | 0.1040 | 0.3820 |
| C93 | 0.0801 | 0.1465 | 0.2121 |
| C94 | 0.1181 | 0.1688 | 0.2016 |
| C95 | 0.1573 | 0.1489 | 0.3570 |
| C96 | 0.1586 | 0.1066 | 0.5120 |
| C97 | 0.1204 | 0.0843 | 0.5279 |
| C98 | 0.8114 | 0.1520 | 0.8087 |
| C99 | 0.2254 | 0.2301 | 0.3790 |
| C100 | 0.2428 | 0.2089 | 0.7121 |
| C101 | 0.2806 | 0.2335 | 0.7466 |
| N102 | 0.2995 | 0.2973 | 0.6890 |
| H103 | 0.2762 | 0.6416 | 0.6880 |
| H104 | 0.5994 | 0.4871 | 0.1296 |
| H105 | 0.4426 | 0.3396 | 0.9616 |
| H106 | 0.3722 | 0.3009 | 0.9788 |
| H107 | 0.3026 | 0.4157 | 0.4042 |
| H108 | 0.3736 | 0.4544 | 0.3692 |
| H109 | 0.2558 | 0.1777 | 0.7731 |
| H110 | 0.2925 | 0.2176 | 0.9967 |
| C111 | 0.4665 | 0.5711 | 0.5842 |
| C112 | 0.4655 | 0.5320 | 0.6139 |
| C113 | 0.4298 | 0.5050 | 0.7607 |
| C114 | 0.5872 | 0.6020 | 0.3510 |
| C115 | 0.5885 | 0.6445 | 0.1813 |
| C116 | 0.6269 | 0.6657 | 0.1845 |
| C117 | 0.6647 | 0.6445 | 0.3535 |
| C118 | 0.6644 | 0.6022 | 0.5071 |
| C119 | 0.6258 | 0.5811 | 0.5089 |
| C120 | 0.3107 | 0.6560 | 0.6646 |
| C121 | 0.7334 | 0.7245 | 0.3922 |

| | | | |
|---|---|---|---|
| C122 | 0.7511 | 0.7030 | 0.7245 |
| C123 | 0.7886 | 0.7278 | 0.7605 |
| N124 | 0.8067 | 0.7916 | 0.7101 |
| H125 | 0.7762 | 0.1385 | 0.8442 |
| H126 | 0.0962 | 0.9906 | 0.1540 |
| H127 | 0.9470 | 0.8355 | 1.0095 |
| H128 | 0.8789 | 0.7958 | 1.0035 |
| H129 | 0.8060 | 0.9093 | 0.4526 |
| H130 | 0.8747 | 0.9492 | 0.4400 |
| H131 | 0.7644 | 0.6720 | 0.7821 |
| H132 | 0.8007 | 0.7117 | 1.0102 |
| C133 | 0.0384 | 0.0802 | 0.4414 |
| C134 | 0.0359 | 0.0443 | 0.4234 |
| C135 | 0.0697 | 0.0342 | 0.2773 |
| C136 | 0.9224 | 0.0860 | 0.7282 |
| C137 | 0.9281 | 0.1106 | 0.8651 |
| C138 | 0.8923 | 0.1327 | 0.8863 |
| C139 | 0.8495 | 0.1291 | 0.7856 |
| C140 | 0.8431 | 0.1036 | 0.6600 |
| C141 | 0.8793 | 0.0830 | 0.6248 |
| C142 | 0.1961 | 0.1716 | 0.3736 |
| C143 | 0.7836 | 0.2121 | 0.8002 |
| C144 | 0.7511 | 0.2246 | 0.5287 |
| C145 | 0.7148 | 0.2523 | 0.5337 |
| N146 | 0.7040 | 0.3169 | 0.5071 |
| H147 | 0.7382 | 0.6520 | 0.4259 |
| H148 | 0.3970 | 0.4476 | 0.8775 |
| H149 | 0.5297 | 0.3901 | 0.0625 |
| H150 | 0.5897 | 0.3449 | 0.0469 |
| H151 | 0.6818 | 0.3848 | 0.5355 |
| H152 | 0.6214 | 0.4289 | 0.5640 |
| H153 | 0.7347 | 0.1964 | 0.5457 |
| H154 | 0.6910 | 0.2609 | 0.3317 |
| C155 | 0.5441 | 0.5797 | 0.3986 |
| C156 | 0.5406 | 0.5436 | 0.3830 |
| C157 | 0.5746 | 0.5317 | 0.2494 |
| C158 | 0.4259 | 0.5909 | 0.6267 |
| C159 | 0.4293 | 0.6164 | 0.7572 |
| C160 | 0.3923 | 0.6385 | 0.7628 |
| C161 | 0.3506 | 0.6342 | 0.6515 |
| C162 | 0.3467 | 0.6083 | 0.5272 |
| C163 | 0.3841 | 0.5877 | 0.5080 |
| C164 | 0.7036 | 0.6662 | 0.3831 |

| | | | |
|---|---|---|---|
| C165 | 0.2781 | 0.7146 | 0.6531 |
| C166 | 0.2485 | 0.7273 | 0.3675 |
| C167 | 0.2095 | 0.7534 | 0.3630 |
| N168 | 0.1897 | 0.8129 | 0.3737 |
| H169 | 0.2310 | 0.1581 | 0.4083 |
| H170 | 0.8966 | 0.9431 | 0.9387 |
| H171 | 0.0404 | 0.8828 | 0.1791 |
| H172 | 0.1080 | 0.8437 | 0.1712 |
| H173 | 0.1896 | 0.9020 | 0.5806 |
| H174 | 0.1211 | 0.9361 | 0.6344 |
| H175 | 0.2347 | 0.6990 | 0.3702 |
| H176 | 0.1889 | 0.7629 | 0.1450 |
| C177 | 0.4990 | 0.4817 | 0.4999 |
| C178 | 0.4922 | 0.4085 | 0.4940 |
| H179 | 0.5092 | 0.6242 | 0.4779 |
| C180 | -0.0025 | 0.9806 | 0.5540 |
| C181 | -0.0041 | 0.9061 | 0.5650 |
| H182 | 0.0018 | 0.1223 | 0.5454 |
| C183 | 0.5017 | 0.5191 | 0.4988 |
| C184 | 0.5065 | 0.5936 | 0.4869 |
| H185 | 0.4885 | 0.3786 | 0.4935 |
| C186 | -0.0016 | 0.0182 | 0.5503 |
| C187 | 0.0012 | 0.0922 | 0.5470 |
| H188 | -0.0055 | 0.8755 | 0.5730 |
| H189 | 0.7436 | 0.7667 | 0.8417 |
| H190 | 0.7408 | 0.8223 | 0.2868 |
| H191 | 0.6877 | 0.7634 | 0.4680 |
| H192 | 0.2362 | 0.2733 | 0.8231 |
| H193 | 0.2335 | 0.3282 | 0.2661 |
| H194 | 0.1800 | 0.2695 | 0.4508 |
| H195 | 0.2468 | 0.8107 | 0.1494 |
| H196 | 0.2703 | 0.8067 | 0.6487 |
| H197 | 0.3172 | 0.7706 | 0.4449 |
| H198 | 0.7561 | 0.3070 | 0.2816 |
| H199 | 0.7851 | 0.3074 | 0.7547 |
| H200 | 0.8259 | 0.2660 | 0.5721 |
| H201 | 0.2538 | 0.2328 | 0.2161 |
| H202 | 0.2144 | 0.2054 | 0.8766 |
| H203 | 0.3095 | 0.2360 | 0.5886 |
| H204 | 0.7616 | 0.7276 | 0.2275 |
| H205 | 0.7227 | 0.6990 | 0.8910 |
| H206 | 0.8176 | 0.7308 | 0.6008 |
| H207 | 0.7644 | 0.1941 | 1.0269 |

| | | | |
|---|---|---|---|
| H208 | 0.7704 | 0.2414 | 0.3013 |
| H209 | 0.6952 | 0.2357 | 0.7602 |
| H210 | 0.2574 | 0.6953 | 0.8706 |
| H211 | 0.2694 | 0.7454 | 0.1491 |
| H212 | 0.1875 | 0.7346 | 0.5746 |

**Table S7.** Atomistic coordinates for the refined unit cell parameters for TFPPery-CHDA COF via Pawley refinement. Space group: *P*1; $a$ = 42.2975 Å, $b$ = 24.4438 Å, $c$ = 4.9193 Å, $\alpha$ = 90.8229°, $\beta$ = 112.3108°, and $\gamma$ = 90.3355°. Pawley-refined PXRD profile confirmed the correctness of the peak assignment as evident by a small difference with $R_{wp}$ and $R_p$ values of 0.66% and 1.36%, respectively.

| | | | |
|---|---|---|---|
| C1 | 0.9040 | 0.9343 | 0.6869 |
| C2 | 0.9031 | 0.9000 | 0.9117 |
| C3 | 0.8721 | 0.8882 | 0.9401 |
| C4 | 0.8416 | 0.9112 | 0.7483 |
| C5 | 0.8425 | 0.9470 | 0.5327 |
| C6 | 0.8732 | 0.9583 | 0.5013 |
| C7 | 0.1711 | 0.7563 | 1.1334 |
| C8 | 0.7718 | 0.8341 | 0.8855 |
| C9 | 0.7448 | 0.8772 | 0.8760 |
| C10 | 0.7104 | 0.8500 | 0.8332 |
| N11 | 0.6817 | 0.7801 | 0.9954 |
| H12 | 0.7069 | 0.4031 | 0.1083 |
| H13 | 0.5660 | 0.7056 | 0.4966 |
| H14 | 0.6190 | 0.7600 | 0.6649 |
| H15 | 0.6577 | 0.6674 | 1.4635 |
| H16 | 0.6051 | 0.6124 | 1.2900 |
| H17 | 0.7404 | 0.9056 | 0.6916 |
| H18 | 0.6989 | 0.8300 | 0.6148 |
| C19 | 0.4130 | 0.3506 | -0.2109 |
| C20 | 0.4092 | 0.3085 | -0.0358 |
| C21 | 0.3792 | 0.2774 | -0.1297 |
| C22 | 0.3526 | 0.2882 | -0.3998 |
| C23 | 0.3568 | 0.3295 | -0.5775 |
| C24 | 0.3867 | 0.3608 | -0.4822 |
| C25 | 0.6849 | 0.3897 | -0.0794 |
| C26 | 0.2803 | 0.1982 | -0.3928 |
| C27 | 0.2524 | 0.2403 | -0.4124 |
| C28 | 0.2181 | 0.2129 | -0.4557 |
| N29 | 0.1894 | 0.1469 | -0.2617 |
| H30 | 0.1894 | 0.7682 | 1.3491 |
| H31 | 0.0667 | 0.1180 | -0.4556 |
| H32 | 0.1221 | 0.1424 | -0.4728 |
| H33 | 0.1730 | 0.0423 | 0.2799 |
| H34 | 0.1190 | 0.0206 | 0.3123 |
| H35 | 0.2487 | 0.2681 | -0.5984 |

| | | | |
|---|---|---|---|
| H36 | 0.2068 | 0.1919 | -0.6719 |
| C37 | 0.0766 | 0.8435 | 0.8054 |
| C38 | 0.0821 | 0.8006 | 0.6373 |
| C39 | 0.1126 | 0.7709 | 0.7447 |
| C40 | 0.1378 | 0.7840 | 1.0211 |
| C41 | 0.1318 | 0.8263 | 1.1902 |
| C42 | 0.1015 | 0.8561 | 1.0817 |
| C43 | 0.8080 | 0.8948 | 0.7458 |
| C44 | 0.2133 | 0.6951 | 1.0837 |
| C45 | 0.2396 | 0.7368 | 1.0653 |
| C46 | 0.2752 | 0.7113 | 1.1398 |
| N47 | 0.3069 | 0.6407 | 0.9974 |
| H48 | 0.3003 | 0.2674 | -0.7076 |
| H49 | 0.4156 | 0.6598 | 0.8546 |
| H50 | 0.3644 | 0.6719 | 0.9631 |
| H51 | 0.3367 | 0.5038 | 0.7301 |
| H52 | 0.3891 | 0.4894 | 0.6469 |
| H53 | 0.2417 | 0.7716 | 1.2193 |
| H54 | 0.2865 | 0.6990 | 1.3742 |
| C55 | 0.5877 | 0.4359 | -0.0610 |
| C56 | 0.5890 | 0.4020 | -0.2909 |
| C57 | 0.6205 | 0.3875 | -0.3043 |
| C58 | 0.6511 | 0.4077 | -0.0928 |
| C59 | 0.6498 | 0.4429 | 0.1284 |
| C60 | 0.6187 | 0.4566 | 0.1453 |
| C61 | 0.3197 | 0.2590 | -0.4935 |
| C62 | 0.7220 | 0.3306 | -0.2212 |
| C63 | 0.7488 | 0.3744 | -0.2054 |
| C64 | 0.7834 | 0.3484 | -0.1630 |
| N65 | 0.8131 | 0.2789 | -0.3257 |
| H66 | 0.7857 | 0.9090 | 0.5665 |
| H67 | 0.9289 | 0.2064 | 0.1823 |
| H68 | 0.8759 | 0.2606 | 0.0109 |
| H69 | 0.8388 | 0.1663 | -0.7973 |
| H70 | 0.8913 | 0.1118 | -0.6215 |
| H71 | 0.7527 | 0.4030 | -0.0186 |
| H72 | 0.7951 | 0.3291 | 0.0545 |
| C73 | 0.0879 | 0.0674 | -0.0692 |
| C74 | 0.0896 | 0.1014 | -0.2922 |
| C75 | 0.1212 | 0.1149 | -0.3048 |
| C76 | 0.1516 | 0.0938 | -0.0987 |
| C77 | 0.1499 | 0.0582 | 0.1150 |
| C78 | 0.1186 | 0.0453 | 0.1305 |

| | | | |
|---|---|---|---|
| C79 | 0.8203 | 0.2457 | -0.5022 |
| C80 | 0.2225 | 0.1718 | -0.2147 |
| C81 | 0.2495 | 0.1287 | -0.2045 |
| C82 | 0.2840 | 0.1557 | -0.1594 |
| N83 | 0.3131 | 0.2257 | -0.3184 |
| H84 | 0.2918 | 0.5744 | 0.7107 |
| H85 | 0.4290 | 0.3012 | 0.1777 |
| H86 | 0.3762 | 0.2460 | 0.0119 |
| H87 | 0.3367 | 0.3385 | -0.7867 |
| H88 | 0.3889 | 0.3941 | -0.6156 |
| H89 | 0.2402 | 0.1041 | -0.4106 |
| H90 | 0.3023 | 0.1239 | -0.1697 |
| C91 | 0.5816 | 0.6559 | 0.8844 |
| C92 | 0.5857 | 0.6979 | 0.7104 |
| C93 | 0.6158 | 0.7287 | 0.8058 |
| C94 | 0.6423 | 0.7176 | 1.0765 |
| C95 | 0.6377 | 0.6765 | 1.2536 |
| C96 | 0.6076 | 0.6455 | 1.1568 |
| C97 | 0.3127 | 0.5988 | 0.8584 |
| C98 | 0.7144 | 0.8078 | 1.0683 |
| C99 | 0.7424 | 0.7658 | 1.0889 |
| C100 | 0.7766 | 0.7934 | 1.1292 |
| N101 | 0.8047 | 0.8590 | 0.9262 |
| H102 | 0.8014 | 0.2366 | -0.7190 |
| H103 | 0.9262 | 0.8819 | 1.0652 |
| H104 | 0.8718 | 0.8605 | 1.1090 |
| H105 | 0.8191 | 0.9643 | 0.3779 |
| H106 | 0.8720 | 0.9832 | 0.3182 |
| H107 | 0.7336 | 0.7399 | 0.8864 |
| H108 | 0.7953 | 0.7618 | 1.1238 |
| C109 | 0.9140 | 0.1560 | -0.2104 |
| C110 | 0.9096 | 0.1984 | -0.0343 |
| C111 | 0.8794 | 0.2290 | -0.1314 |
| C112 | 0.8534 | 0.2173 | -0.4057 |
| C113 | 0.8584 | 0.1758 | -0.5846 |
| C114 | 0.8884 | 0.1451 | -0.4863 |
| C115 | 0.1855 | 0.1115 | -0.0823 |
| C116 | 0.7801 | 0.3056 | -0.4000 |
| C117 | 0.7525 | 0.2627 | -0.4245 |
| C118 | 0.7179 | 0.2892 | -0.4663 |
| N119 | 0.6888 | 0.3549 | -0.2657 |
| H120 | 0.6946 | 0.7381 | 1.3842 |
| H121 | 0.5659 | 0.3864 | -0.4598 |

| | | | |
|---|---|---|---|
| H122 | 0.6211 | 0.3603 | -0.4780 |
| H123 | 0.6732 | 0.4581 | 0.2978 |
| H124 | 0.6195 | 0.4810 | 0.3329 |
| H125 | 0.7613 | 0.2378 | -0.2227 |
| H126 | 0.6995 | 0.2571 | -0.4645 |
| C127 | 0.4068 | 0.5736 | 0.7378 |
| C128 | 0.3995 | 0.6246 | 0.8366 |
| C129 | 0.3701 | 0.6318 | 0.8986 |
| C130 | 0.3469 | 0.5887 | 0.8554 |
| C131 | 0.3543 | 0.5376 | 0.7651 |
| C132 | 0.3841 | 0.5298 | 0.7113 |
| C133 | 0.6752 | 0.7466 | 1.1704 |
| C134 | 0.2727 | 0.6615 | 0.9423 |
| C135 | 0.2486 | 0.6187 | 0.9871 |
| C136 | 0.2125 | 0.6431 | 0.9044 |
| N137 | 0.1794 | 0.7199 | 0.9774 |
| H138 | 0.2075 | 0.0981 | 0.1027 |
| H139 | 0.0634 | 0.7916 | 0.4195 |
| H140 | 0.1170 | 0.7387 | 0.6097 |
| H141 | 0.1509 | 0.8372 | 1.4038 |
| H142 | 0.0980 | 0.8901 | 1.2094 |
| H143 | 0.2589 | 0.6056 | 1.2199 |
| H144 | 0.1954 | 0.6127 | 0.9453 |
| H145 | 0.7629 | 0.8103 | 0.6778 |
| H146 | 0.7541 | 0.9020 | 1.0814 |
| H147 | 0.6920 | 0.8818 | 0.8431 |
| H148 | 0.2736 | 0.1756 | -0.6031 |
| H149 | 0.2611 | 0.2657 | -0.2083 |
| H150 | 0.1994 | 0.2446 | -0.4499 |
| H151 | 0.2199 | 0.6829 | 1.3137 |
| H152 | 0.2303 | 0.7536 | 0.8394 |
| H153 | 0.2926 | 0.7419 | 1.1052 |
| H154 | 0.7308 | 0.3075 | -0.0147 |
| H155 | 0.7395 | 0.3988 | -0.4085 |
| H156 | 0.8015 | 0.3807 | -0.1701 |
| H157 | 0.2312 | 0.1953 | -0.0057 |
| H158 | 0.2536 | 0.1003 | -0.0210 |
| H159 | 0.2955 | 0.1755 | 0.0596 |
| H160 | 0.7210 | 0.8306 | 1.2776 |
| H161 | 0.7464 | 0.7383 | 1.2765 |
| H162 | 0.7878 | 0.8147 | 1.3443 |
| H163 | 0.7734 | 0.3276 | -0.6083 |
| H164 | 0.7490 | 0.2346 | -0.6127 |

| | | | |
|---|---|---|---|
| H165 | 0.7067 | 0.3098 | -0.6802 |
| H166 | 0.2621 | 0.6751 | 0.7125 |
| H167 | 0.2467 | 0.5820 | 0.8463 |
| H168 | 0.2011 | 0.6527 | 0.6666 |
| H169 | 0.9597 | 0.8664 | 0.8193 |
| H170 | 0.9222 | 1.0218 | 0.4564 |
| H171 | 0.0312 | 0.1335 | -0.2212 |
| H172 | 0.0692 | -0.0212 | 0.1457 |
| H173 | 0.9167 | 0.0689 | 0.0950 |
| H174 | 0.9743 | 0.1611 | -0.2800 |
| H175 | 0.0161 | 0.8378 | 0.8692 |
| H176 | 0.0739 | 0.9305 | 0.5002 |
| C177 | 0.0290 | 0.0969 | -0.1089 |
| C178 | 0.0551 | 0.0580 | -0.0265 |
| C179 | 0.0511 | 0.0111 | 0.1179 |
| C180 | 0.0240 | 0.0053 | 0.2155 |
| C181 | 0.9410 | 0.0749 | 0.0762 |
| C182 | 0.9683 | 0.0382 | 0.1987 |
| C183 | 0.9975 | 0.0444 | 0.1258 |
| C184 | 0.9998 | 0.0895 | -0.0441 |
| C185 | 0.9728 | 0.1269 | -0.1478 |
| C186 | 0.9433 | 0.1190 | -0.0912 |
| C187 | -0.0069 | 0.9549 | 0.4663 |
| C188 | -0.0093 | 0.9097 | 0.6358 |
| C189 | 0.0175 | 0.8719 | 0.7362 |
| C190 | 0.0470 | 0.8796 | 0.6791 |
| C191 | 0.0495 | 0.9242 | 0.5151 |
| C192 | 0.0222 | 0.9611 | 0.3935 |
| C193 | 0.4373 | 0.5259 | 0.4509 |
| C194 | 0.4664 | 0.5147 | 0.3849 |
| C195 | 0.4952 | 0.5495 | 0.5083 |
| C196 | 0.4950 | 0.5917 | 0.7085 |
| C197 | 0.4667 | 0.5985 | 0.7874 |
| C198 | 0.4374 | 0.5661 | 0.6580 |
| C199 | 0.5233 | 0.6264 | 0.8330 |
| C200 | 0.5520 | 0.6190 | 0.7644 |
| C201 | 0.5529 | 0.5766 | 0.5756 |
| C202 | 0.5244 | 0.5421 | 0.4367 |
| C203 | 0.4677 | 0.4699 | 0.2011 |
| C204 | 0.4970 | 0.4626 | 0.1295 |
| C205 | 0.4984 | 0.4179 | -0.0535 |
| C206 | 0.4709 | 0.3815 | -0.1662 |
| C207 | 0.4423 | 0.3882 | -0.0945 |

| | | | |
|---|---|---|---|
| C208 | 0.4410 | 0.4312 | 0.0895 |
| C209 | 0.5276 | 0.4096 | -0.1185 |
| C210 | 0.5549 | 0.4467 | -0.0192 |
| C211 | 0.5520 | 0.4929 | 0.1416 |
| C212 | 0.5247 | 0.4996 | 0.2375 |
| H213 | 0.4136 | 0.5050 | 0.3363 |
| H214 | 0.4680 | 0.6286 | 0.9545 |
| H215 | 0.5231 | 0.6589 | 0.9842 |
| H216 | 0.5768 | 0.5707 | 0.5501 |
| H217 | 0.4716 | 0.3478 | -0.3092 |
| H218 | 0.4185 | 0.4332 | 0.1399 |
| H219 | 0.5290 | 0.3734 | -0.2425 |
| H220 | 0.5711 | 0.5241 | 0.1834 |
| C221 | 0.9667 | 0.9942 | 0.3788 |
| C222 | 0.9619 | 0.9029 | 0.7059 |
| C223 | 0.9361 | 0.9425 | 0.6296 |
| C224 | 0.9399 | 0.9890 | 0.4814 |

**Table S8** Atomistic coordinates for the refined unit cell parameters for TFPPery-PDA COF via Pawley refinement. Space group: *P*6; $a = b = 51.9929$ Å, $c = 3.4400$ Å, $\alpha = 90.0000^{\circ}$, $\beta = 90.0000^{\circ}$, and $\gamma = 120.0000^{\circ}$. Pawley-refined PXRD profile confirmed the correctness of the peak assignment as evident by a small difference with $R_{wp}$ and $R_p$ values of 1.14% and 2.79%, respectively.

| | | | |
|---|---|---|---|
| C1 | 0.4730 | 0.0006 | 0.5707 |
| C2 | 0.4728 | 0.5007 | 0.5584 |
| C3 | 0.5006 | 0.5278 | 0.5829 |
| C4 | 0.5027 | 0.5561 | 0.6231 |
| C5 | 0.5299 | 0.5835 | 0.6019 |
| C6 | 0.5560 | 0.5816 | 0.5729 |
| C7 | 0.5553 | 0.5541 | 0.5704 |
| C8 | 0.5823 | 0.5536 | 0.5669 |
| C9 | 0.5829 | 0.5268 | 0.5363 |
| C10 | 0.5549 | 0.5003 | 0.5170 |
| C11 | 0.5307 | 0.6114 | 0.6054 |
| C12 | 0.3898 | 0.4736 | 0.5278 |
| C13 | 0.6121 | 0.5016 | 0.6610 |
| C14 | 0.6389 | 0.5010 | 0.6536 |
| C15 | 0.6654 | 0.5257 | 0.5240 |
| C16 | 0.6641 | 0.5507 | 0.3923 |
| C17 | 0.6371 | 0.5508 | 0.3900 |
| C18 | 0.5557 | 0.6379 | 0.7398 |
| C19 | 0.5568 | 0.6656 | 0.7300 |
| C20 | 0.5325 | 0.6678 | 0.5965 |
| C21 | 0.5072 | 0.6417 | 0.4682 |
| C22 | 0.5065 | 0.6143 | 0.4684 |
| N23 | 0.6903 | 0.5234 | 0.5346 |
| C24 | 0.7192 | 0.5441 | 0.4846 |
| C25 | 0.7418 | 0.5365 | 0.5004 |
| N26 | 0.5353 | 0.6953 | 0.5951 |
| C27 | 0.5148 | 0.7037 | 0.5392 |
| C28 | 0.7721 | 0.5593 | 0.5049 |
| C29 | 0.7951 | 0.5524 | 0.5204 |
| C30 | 0.7886 | 0.5225 | 0.5292 |
| C31 | 0.7584 | 0.4997 | 0.5241 |
| C32 | 0.7354 | 0.5066 | 0.5110 |
| H33 | 0.4840 | 0.5571 | 0.6755 |
| H34 | 0.5763 | 0.6007 | 0.5504 |
| H35 | 0.6018 | 0.5734 | 0.5890 |

| | | | |
|---|---|---|---|
| H36 | 0.5550 | 0.4811 | 0.4644 |
| H37 | 0.5940 | 0.4835 | 0.7718 |
| H38 | 0.6391 | 0.4825 | 0.7492 |
| H39 | 0.6828 | 0.5689 | 0.2922 |
| H40 | 0.6374 | 0.5691 | 0.2807 |
| H41 | 0.5736 | 0.6375 | 0.8509 |
| H42 | 0.5755 | 0.6841 | 0.8250 |
| H43 | 0.4891 | 0.6424 | 0.3666 |
| H44 | 0.4880 | 0.5965 | 0.3592 |
| H47 | 0.7777 | 0.5812 | 0.4976 |
| H48 | 0.8166 | 0.5696 | 0.5233 |
| H49 | 0.7527 | 0.4778 | 0.5302 |
| H50 | 0.7139 | 0.4894 | 0.5079 |

**Table S9**. Cohesive energy values (*E*c) for the TFPPery-CHDA-COF and TFPPery-PDA-COF in the eclipsed stacking structures according to the DFT calculation.

| | Interlayer Distance (Å) | LJ Energy (a.u.) | Per Layer Stabilization (kcal/mol) | $E_c$ (kcal/mol) |
|---|---|---|---|---|
| TFPPery-CHDA-COF (kgm) | 3.44 | 3.1061 | -193.11 | -64.37 |
| TFPPery-CHDA-COF (sql) | 3.41 | 2.0491 | -138.33 | -69.16 |
| TFPPery-PDA-COF (kgm) | 3.59 | 2.6737 | -229.16 | -76.39 |
| TFPPery-PDA-COF (sql) | 3.59 | 1.7973 | -148.66 | -74.33 |

**Table S10.** Comparison with some previously reported limits of detection for $Fe^{3+}$, picric acid and phenyl glyoxylic acid.

| Sample name | Wavelength | Solvent | Stern-Volmer Quenching Rate Constant ($K_{sv}$) | Limit of Detection (LOD) | Reference |
|---|---|---|---|---|---|
| Detection for $Fe^{3+}$ | | | | | |
| **ETTA-CHDA COF** | **544 nm** | **THF** | **$2.00 \times 10^4$ M$^{-1}$** | **680 ppb** | **Our Work** |
| HMQC-MG | 470 nm | water | n/a | 0.199 $\mu$M | *ACS Appl. Polym. Mater.* **2020,** *2* (8), 3621–3631 |
| JUC-557 nanosheet | 514 | ethanol /water = 1 : 9 | $1.98 \times 10^4$ M$^{-1}$ | 706 ppb | CCS Chem. **2022**, DOI:10.31635/ccschem.022.202 |
| COP-1 | 570 nm | *N,N*-dimethylacetamide | n/a | 0.42 $\mu$M | *Chem. Commun.* **2019**, *55* (82), 12328–12331 |
| Detection for Picric Acid | | | | | |
| **ETTA-CHDA COF** | **544 nm** | **THF** | **$3.04 \times 10^5$ M$^{-1}$** | **188 ppb** | **Our Work** |
| USTC-7 | 370 nm | $CHCl_3$ | $4.90 \times 10^4$ M$^{-1}$ | $2.78 \times 10^{-4}$ mM | *Chem. Commun.* **2016,** *52* (33), 5734–5737 |
| Py-azine COF | 522 nm | $CH_3CN$ | $7.8 \times 10^4$ M$^{-1}$ | n/a | *J. Am. Chem. Soc.* **2013,** *135* (46), 17310−17313 |

| | | | | | |
|---|---|---|---|---|---|
| PI-COF | 500 nm | ethanol | $1 \times 10^{7}$ $M^{-1}$ | 0.25 $\mu$M | *ACS Appl. Mater. Interfaces* **2017,** *9* (15), 13415−13421 |
| [Cd(NDC)0.5(PCA)]·Gx | 384 nm | $CH_3CN$ | $3.5 \times 10^{4}$ $M^{-1}$ | n/a | *Angew. Chem. Int. Ed.* **2013,** *52* (10), 2881–2885 |
| Detection for Phenyl Glyoxylic Acid | | | | | |
| **ETTA-CHDA COF** | **544 nm** | **THF** | **$2.83 \times 10^{5}$ $M^{-1}$** | **134 ppb** | **Our Work** |
| Tb-MOF 1 | 546 nm | water | $74.38 \times 10^{-3}$ $M^{-1}$ | $1.05 \times 10^{-4}$ mg/mL | *ACS Appl. Mater. Interfaces* **2021,** *13* (28), 33546–33556. |
| Zn-PBA | 422 nm | water | $1.26 \times 10^{3}$ $M^{-1}$ | $1.67 \times 10^{-3}$ M | *Appl. Organomet. Chem.* **2022,** *36* (1), e6468. |
| Eu@MOFs | 322 nm | $CH_3CN$ | n/a | 1.012 ppm | *Biosens. Bioelectron.* **2017,** *97*, 299–304. |

## Section E: References

1. Jiang, S.; Bacsa, J.; Wu, X.; Jones, J. T. A.; Dawson, R.; Trewin, A.; Adams, D. J.; Cooper, A. I. Selective Gas Sorption in a [2+3] 'Propeller' Cage Crystal. *Chem. Commun.* **2011,** *47*, 8919–8921.

2. Jin, E.; Asada, M.; Xu, Q.; Dalapati, S.; Addicoat Matthew, A.; Brady Michael, A.; Xu, H.; Nakamura, T.; Heine, T.; Chen, Q.; Jiang, D. Two-Dimensional $sp^2$ Carbon-Conjugated Covalent Organic Frameworks. *Science* **2017,** *357*, 673–676.

3. Jin, E.; Li, J.; Geng, K.; Jiang, Q.; Xu, H.; Xu, Q.; Jiang, D. Designed Synthesis of Stable Light-emitting Two-Dimensional $sp^2$ Carbon-Conjugated Covalent Organic Frameworks. *Nat. Commun.* **2018,** *9*, 4143.

4. Jin, E.; Lan, Z.; Jiang, Q.; Geng, K.; Li, G.; Wang, X.; Jiang, D. 2D $sp^2$ Carbon-Conjugated Covalent Organic Frameworks for Photocatalytic Hydrogen Production from Water. *Chem* **2019,** *5*, 1632–1647.

5. Zhuang, X.; Zhao, W.; Zhang, F.; Cao, Y.; Liu, F.; Bi, S.; Feng, X. A Two-Dimensional Conjugated Polymer Framework with Fully $sp^2$-Bonded Carbon Skeleton. *Polym. Chem.* **2016,** *7*, 4176–4181.

6. Chen, R.; Shi, J.-L.; Ma, Y.; Lin, G.; Lang, X.; Wang, C. Designed Synthesis of a 2D Porphyrin-Based $sp^2$ Carbon-Conjugated Covalent Organic Framework for Heterogeneous Photocatalysis. *Angew. Chem. Int. Ed.* **2019,** *58*, 6430–6434.

7. Xu, S.; Wang, G.; Biswal, B. P.; Addicoat, M.; Paasch, S.; Sheng, W.; Zhuang, X.; Brunner, E.; Heine, T.; Berger, R.; Feng, X. A Nitrogen-Rich 2D $sp^2$-Carbon-Linked Conjugated Polymer Framework as a High-Performance Cathode for Lithium-Ion Batteries. *Angew. Chem. Int. Ed.* **2019,** *58*, 849–863.

8. Ascherl, L.; Evans, E. W.; Gorman, J.; Orsborne, S.; Bessinger, D.; Bein, T.; Friend, R. H.; Auras, F. Perylene-Based Covalent Organic Frameworks for Acid Vapor Sensing. *J. Am. Chem. Soc.* **2019,** *141*, 15693–15699.

9. Xu, X.; Zhang, S.; Xu, K.; Chen, H.; Fan, X.; Huang, N. Janus Dione-Based Conjugated Covalent Organic Frameworks with High Conductivity as Superior Cathode Materials. *J. Am. Chem. Soc.* **2022,** *145*, 1022–1030.

10. Jin, E.; Geng, K.; Lee, K. H.; Jiang, W.; Li, J.; Jiang, Q.; Irle, S.; Jiang, D. Topology-Templated Synthesis of Crystalline Porous Covalent Organic Frameworks. *Angew. Chem. Int. Ed.* **2020,** *59*, 12162–12169.

11. Porrès, L.; Holland, A.; Pålsson, L.-O.; Monkman, A. P.; Kemp, C.; Beeby, A. Absolute Measurements of Photoluminescence Quantum Yields of Solutions using an Integrating Sphere. *J. Fluoresc.* **2006,** *16*, 267–273.

12. Lee, C.; Yang, W.; Parr, R. G. Development of the Colle-Salvetti Correlation-Energy Formula into a Functional of the Electron Density. *Phys. Rev. B* **1988,** *37*, 785.

13. Becke, A. D. Density-Functional Thermochemistry. IV. A New Dynamical Correlation Functional and Implications for Exact-Exchange Mixing. *J. Phys. Chem. C* **1996,** *104*, 1040–1046.

14. Hourahine, B.; Sanna, S.; Aradi, B.; Köhler, C.; Niehaus, T.; Frauenheim, T. Self-Interaction and Strong Correlation in DFTB. *J. Phys. Chem. A* **2007,** *111*, 5671–5677.

15. Elstner, M.; Porezag, D.; Jungnickel, G.; Elsner, J.; Haugk, M.; Frauenheim, T.; Suhai, S.; Seifert, G. Self-Consistent-Charge Density-Functional Tight-Binding Method for Simulations of Complex Materials Properties. *Phys. Rev. B* **1998,** *58*, 7260.

16. Hosokawa, F.; Shinkawa, T.; Arai, Y.; Sannomiya, T. Benchmark Test of Accelerated Multi-Slice Simulation by GPGPU. *Ultramicroscopy* **2015**, *158*, 56–64.
